# Supplementary material for: Characterization of Lipid Profiles after Dietary Intake of Polyunsaturated Fatty Acids Using Integrated Untargeted and Targeted Lipidomics
Source: Metabolites. 2019 Oct 21;9(10):241. doi: 10.3390/metabo9100241 (PMC6836067; doi:10.3390/metabo9100241)
Supplement: Supplementary file 1 [file metabolites-09-00241-s001.zip › Supplementary Files/Supplementary Figure 1.pdf]

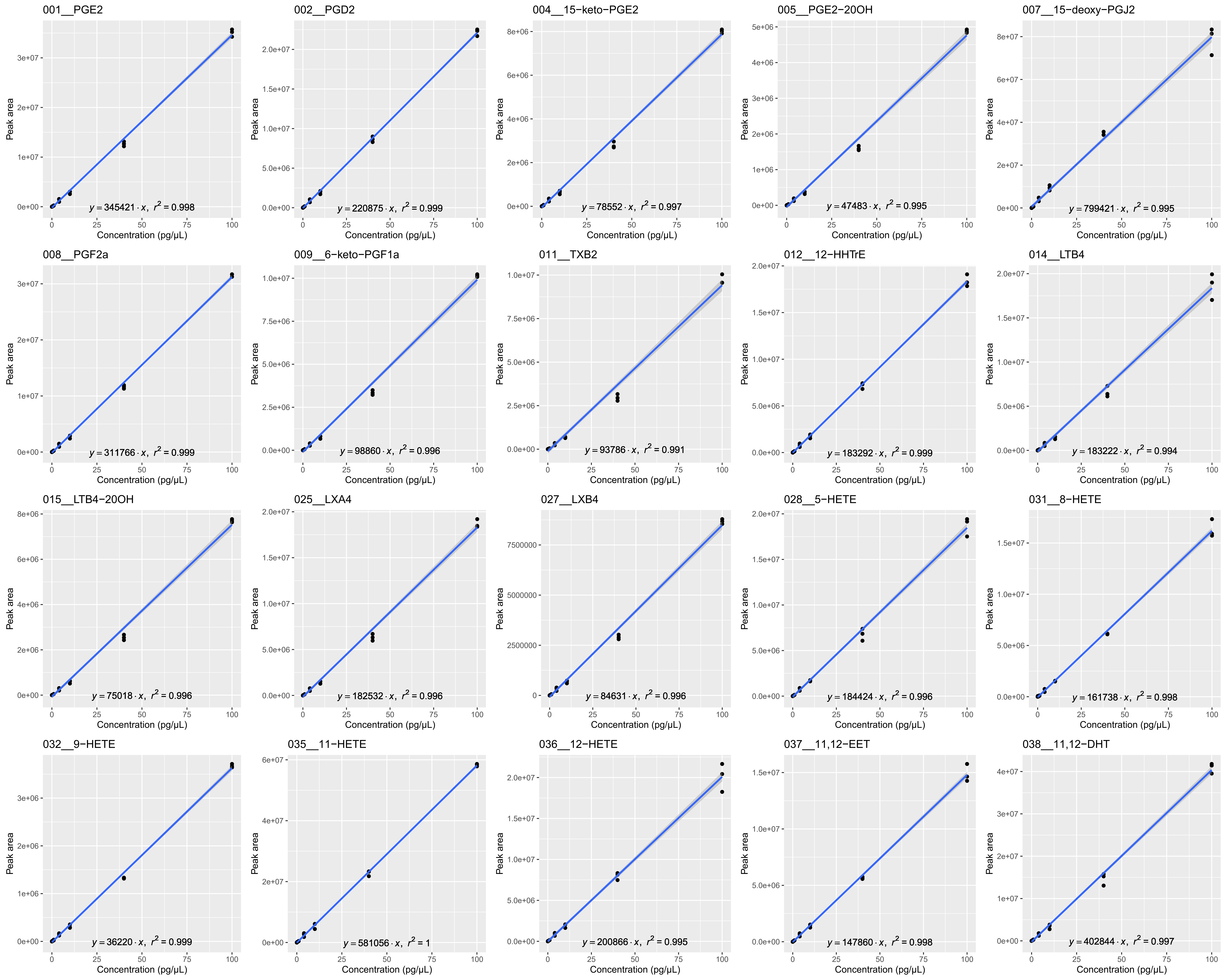
001__PGE2
y = 345421 · x, r^2 = 0.998
002__PGD2
y = 220875 · x, r^2 = 0.999
004__15−keto−PGE2
y = 78552 · x, r^2 = 0.997
005__PGE2−20OH
y = 47483 · x, r^2 = 0.995
007__15−deoxy−PGJ2
y = 799421 · x, r^2 = 0.995
008__PGF2a
y = 311766 · x, r^2 = 0.999
009__6−keto−PGF1a
y = 98860 · x, r^2 = 0.996
011__TXB2
y = 93786 · x, r^2 = 0.991
012__12−HHTrE
y = 183292 · x, r^2 = 0.999
014__LTB4
y = 183222 · x, r^2 = 0.994
015__LTB4−20OH
y = 75018 · x, r^2 = 0.996
025__LXA4
y = 182532 · x, r^2 = 0.996
027__LXB4
y = 84631 · x, r^2 = 0.996
028__5−HETE
y = 184424 · x, r^2 = 0.996
031__8−HETE
y = 161738 · x, r^2 = 0.998
032__9−HETE
y = 36220 · x, r^2 = 0.999
035__11−HETE
y = 581056 · x, r^2 = 1
036__12−HETE
y = 200866 · x, r^2 = 0.995
037__11,12−EET
y = 147860 · x, r^2 = 0.998
038__11,12−DHT
y = 402844 · x, r^2 = 0.997
Peak area
Concentration (pg/μL)

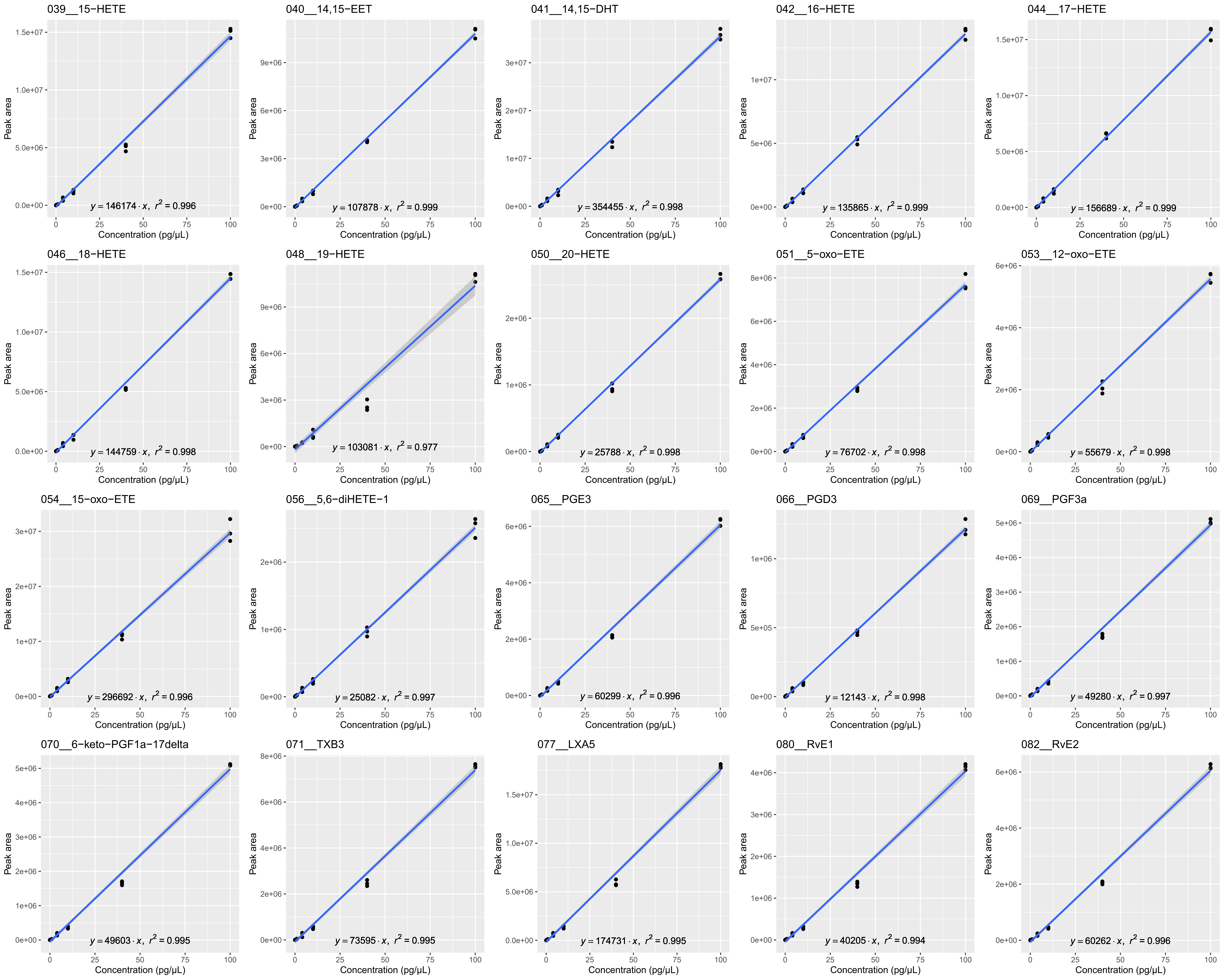

039__15−HETE
y = 146174 · x, r² = 0.996
040__14,15−EET
y = 107878 · x, r² = 0.999
041__14,15−DHT
y = 354455 · x, r² = 0.998
042__16−HETE
y = 135865 · x, r² = 0.999
044__17−HETE
y = 156689 · x, r² = 0.999
046__18−HETE
y = 144759 · x, r² = 0.998
048__19−HETE
y = 103081 · x, r² = 0.977
050__20−HETE
y = 25788 · x, r² = 0.998
051__5−oxo−ETE
y = 76702 · x, r² = 0.998
053__12−oxo−ETE
y = 55679 · x, r² = 0.998
054__15−oxo−ETE
y = 296692 · x, r² = 0.996
056__5,6−diHETE−1
y = 25082 · x, r² = 0.997
065__PGE3
y = 60299 · x, r² = 0.996
066__PGD3
y = 12143 · x, r² = 0.998
069__PGF3a
y = 49280 · x, r² = 0.997
070__6−keto−PGF1a−17delta
y = 49603 · x, r² = 0.995
071__TXB3
y = 73595 · x, r² = 0.995
077__LXA5
y = 174731 · x, r² = 0.995
080__RvE1
y = 40205 · x, r² = 0.994
082__RvE2
y = 60262 · x, r² = 0.996
Peak area
Concentration (pg/μL)

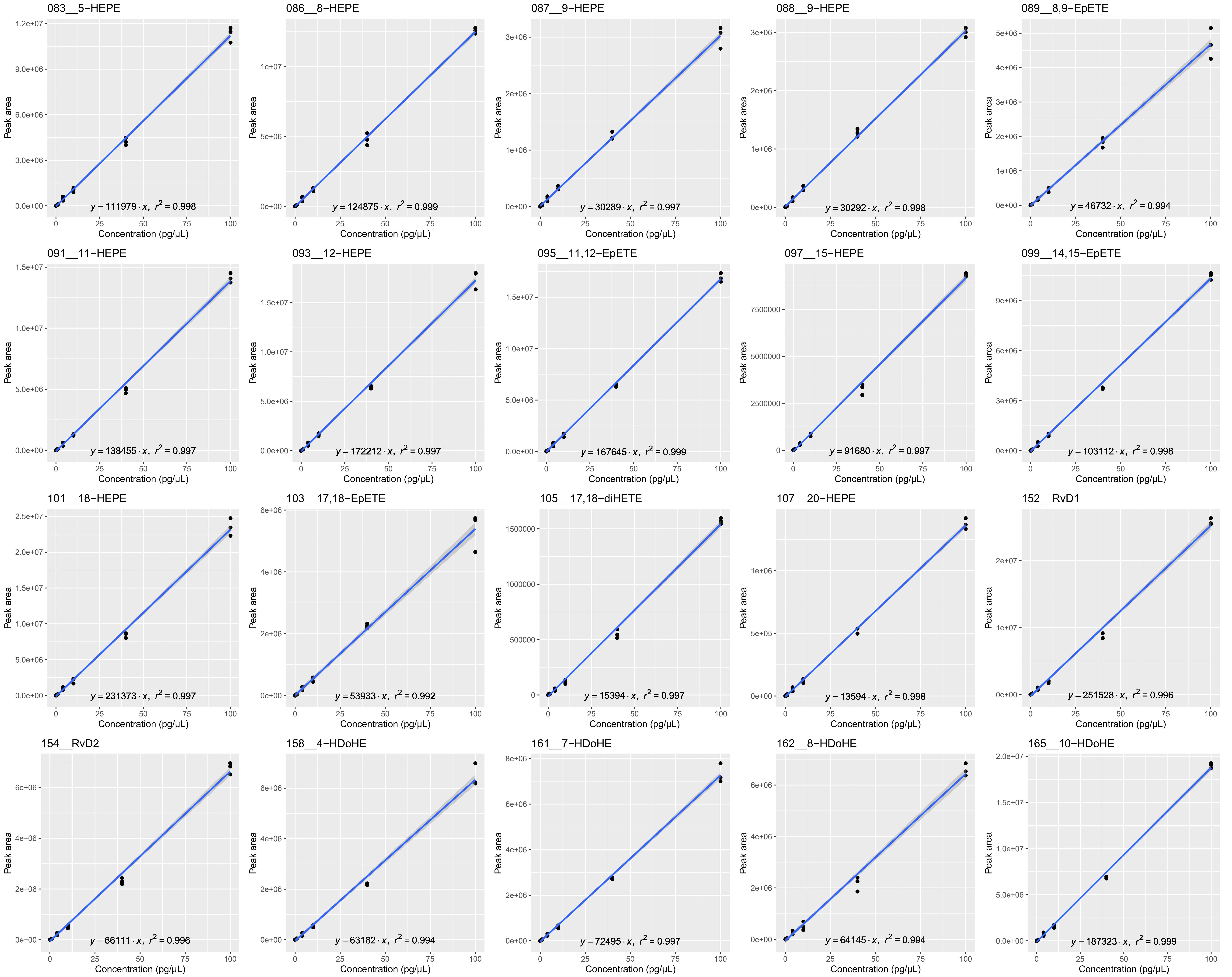
083__5−HEPE
y = 111979 · x, r^2 = 0.998
086__8−HEPE
y = 124875 · x, r^2 = 0.999
087__9−HEPE
y = 30289 · x, r^2 = 0.997
088__9−HEPE
y = 30292 · x, r^2 = 0.998
089__8,9−EpETE
y = 46732 · x, r^2 = 0.994
091__11−HEPE
y = 138455 · x, r^2 = 0.997
093__12−HEPE
y = 172212 · x, r^2 = 0.997
095__11,12−EpETE
y = 167645 · x, r^2 = 0.999
097__15−HEPE
y = 91680 · x, r^2 = 0.997
099__14,15−EpETE
y = 103112 · x, r^2 = 0.998
101__18−HEPE
y = 231373 · x, r^2 = 0.997
103__17,18−EpETE
y = 53933 · x, r^2 = 0.992
105__17,18−diHETE
y = 15394 · x, r^2 = 0.997
107__20−HEPE
y = 13594 · x, r^2 = 0.998
152__RvD1
y = 251528 · x, r^2 = 0.996
154__RvD2
y = 66111 · x, r^2 = 0.996
158__4−HDoHE
y = 63182 · x, r^2 = 0.994
161__7−HDoHE
y = 72495 · x, r^2 = 0.997
162__8−HDoHE
y = 64145 · x, r^2 = 0.994
165__10−HDoHE
y = 187323 · x, r^2 = 0.999
Peak area
Concentration (pg/μL)

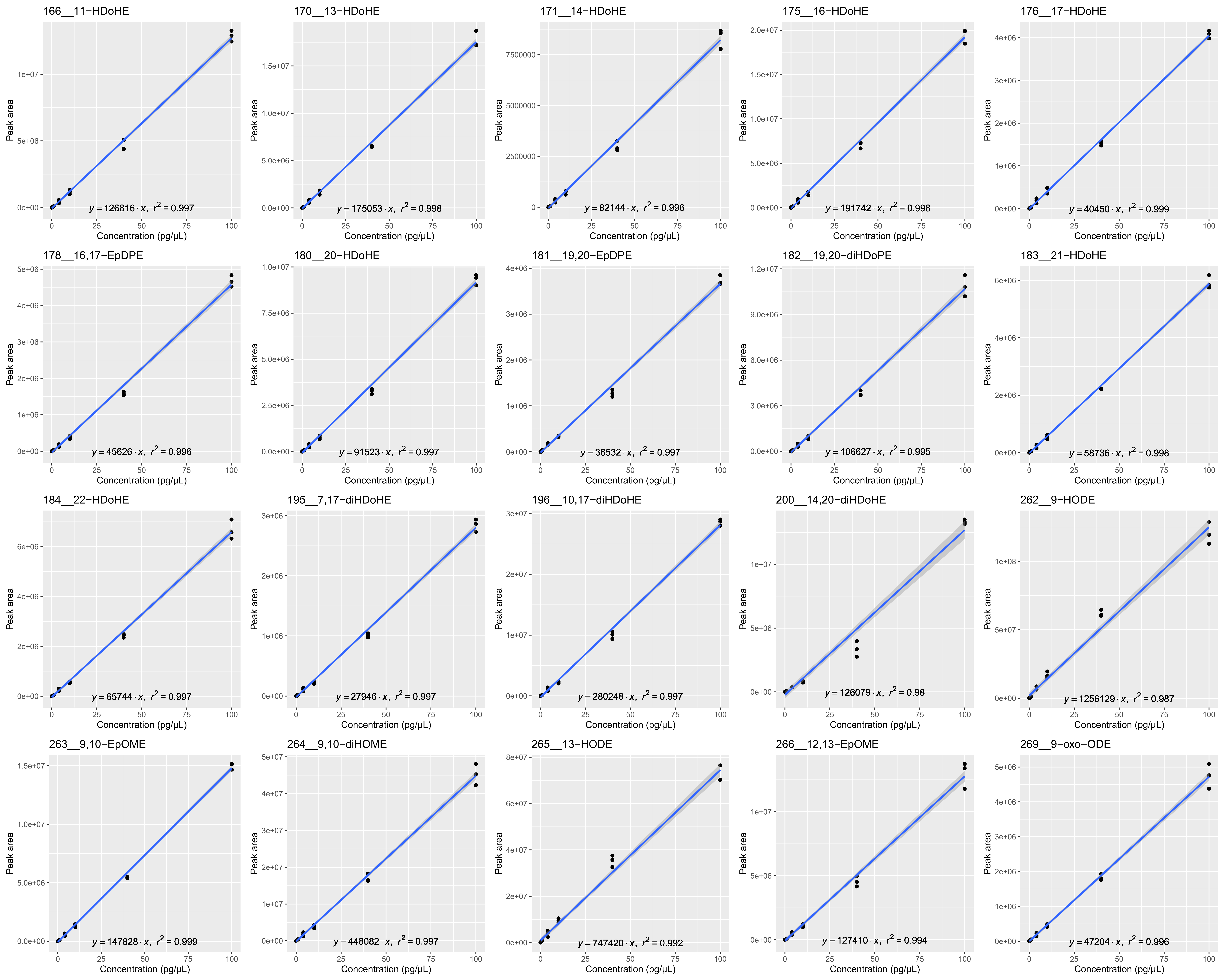

166__11−HDoHE
y = 126816 · x, r² = 0.997
170__13−HDoHE
y = 175053 · x, r² = 0.998
171__14−HDoHE
y = 82144 · x, r² = 0.996
175__16−HDoHE
y = 191742 · x, r² = 0.998
176__17−HDoHE
y = 40450 · x, r² = 0.999
178__16,17−EpDPE
y = 45626 · x, r² = 0.996
180__20−HDoHE
y = 91523 · x, r² = 0.997
181__19,20−EpDPE
y = 36532 · x, r² = 0.997
182__19,20−diHDoPE
y = 106627 · x, r² = 0.995
183__21−HDoHE
y = 58736 · x, r² = 0.998
184__22−HDoHE
y = 65744 · x, r² = 0.997
195__7,17−diHDoHE
y = 27946 · x, r² = 0.997
196__10,17−diHDoHE
y = 280248 · x, r² = 0.997
200__14,20−diHDoHE
y = 126079 · x, r² = 0.98
262__9−HODE
y = 1256129 · x, r² = 0.987
263__9,10−EpOME
y = 147828 · x, r² = 0.999
264__9,10−diHOME
y = 448082 · x, r² = 0.997
265__13−HODE
y = 747420 · x, r² = 0.992
266__12,13−EpOME
y = 127410 · x, r² = 0.994
269__9−oxo−ODE
y = 47204 · x, r² = 0.996
Peak area
Concentration (pg/μL)

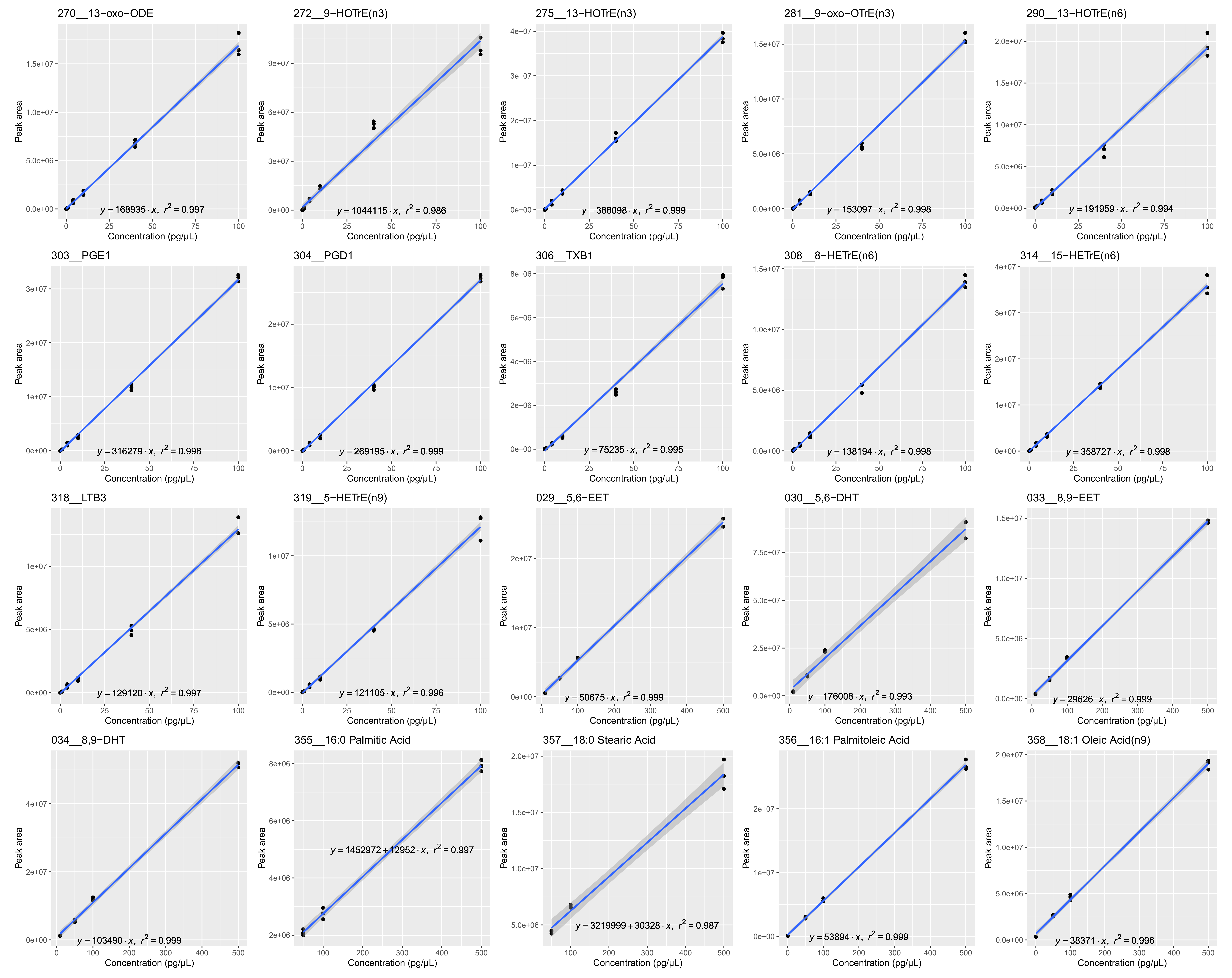
270__13-oxo-ODE
y = 168935 · x, r² = 0.997
272__9-HOTrE(n3)
y = 1044115 · x, r² = 0.986
275__13-HOTrE(n3)
y = 388098 · x, r² = 0.999
281__9-oxo-OTrE(n3)
y = 153097 · x, r² = 0.998
290__13-HOTrE(n6)
y = 191959 · x, r² = 0.994
303__PGE1
y = 316279 · x, r² = 0.998
304__PGD1
y = 269195 · x, r² = 0.999
306__TXB1
y = 75235 · x, r² = 0.995
308__8-HETrE(n6)
y = 138194 · x, r² = 0.998
314__15-HETrE(n6)
y = 358727 · x, r² = 0.998
318__LTB3
y = 129120 · x, r² = 0.997
319__5-HETrE(n9)
y = 121105 · x, r² = 0.996
029__5,6-EET
y = 50675 · x, r² = 0.999
030__5,6-DHT
y = 176008 · x, r² = 0.993
033__8,9-EET
y = 29626 · x, r² = 0.999
034__8,9-DHT
y = 103490 · x, r² = 0.999
355__16:0 Palmitic Acid
y = 1452972 + 12952 · x, r² = 0.997
357__18:0 Stearic Acid
y = 3219999 + 30328 · x, r² = 0.987
356__16:1 Palmitoleic Acid
y = 53894 · x, r² = 0.999
358__18:1 Oleic Acid(n9)
y = 38371 · x, r² = 0.996
Peak area
Concentration (pg/μL)

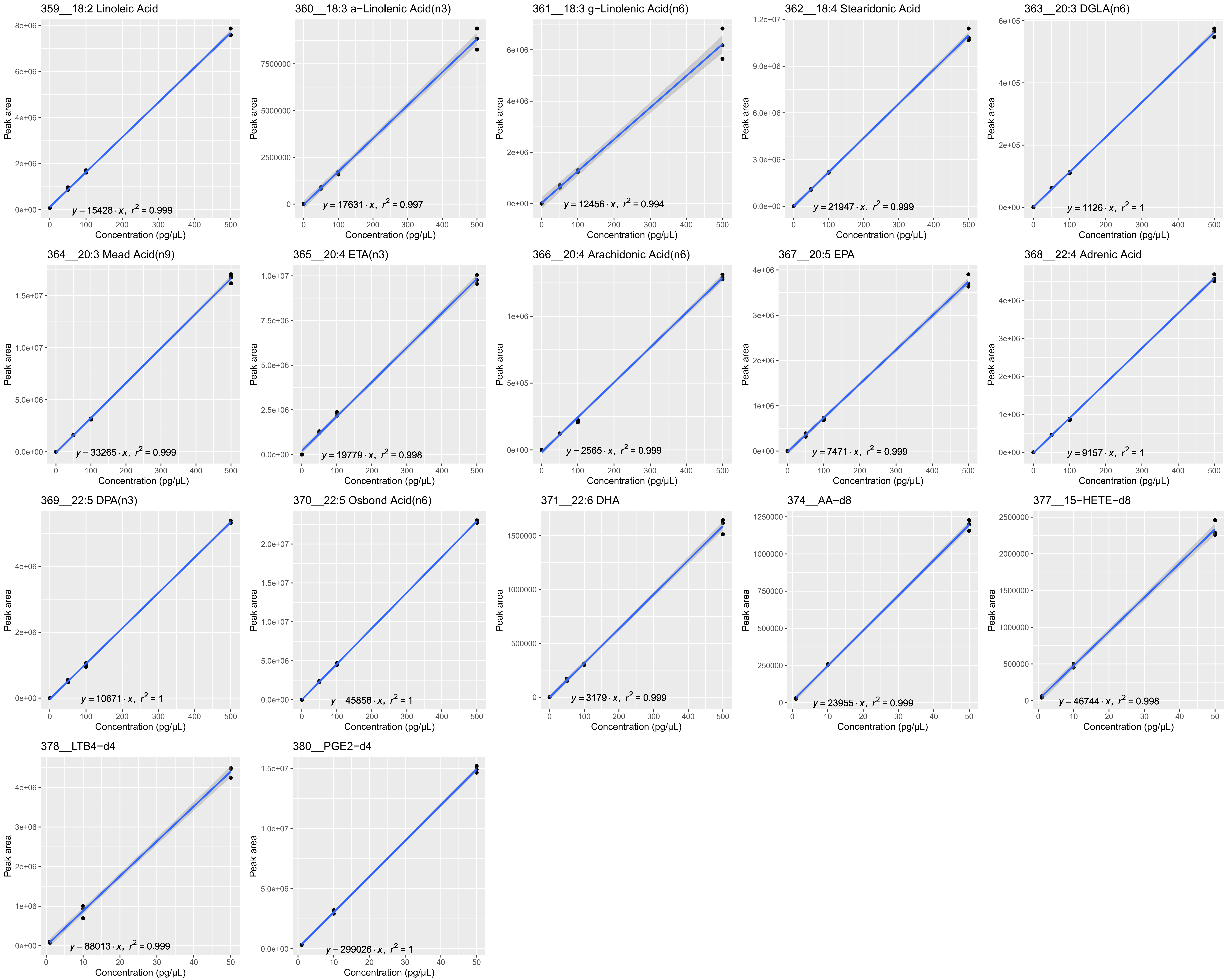

359__18:2 Linoleic Acid
y = 15428 · x, r² = 0.999
360__18:3 a−Linolenic Acid(n3)
y = 17631 · x, r² = 0.997
361__18:3 g−Linolenic Acid(n6)
y = 12456 · x, r² = 0.994
362__18:4 Stearidonic Acid
y = 21947 · x, r² = 0.999
363__20:3 DGLA(n6)
y = 1126 · x, r² = 1
364__20:3 Mead Acid(n9)
y = 33265 · x, r² = 0.999
365__20:4 ETA(n3)
y = 19779 · x, r² = 0.998
366__20:4 Arachidonic Acid(n6)
y = 2565 · x, r² = 0.999
367__20:5 EPA
y = 7471 · x, r² = 0.999
368__22:4 Adrenic Acid
y = 9157 · x, r² = 1
369__22:5 DPA(n3)
y = 10671 · x, r² = 1
370__22:5 Osbond Acid(n6)
y = 45858 · x, r² = 1
371__22:6 DHA
y = 3179 · x, r² = 0.999
374__AA−d8
y = 23955 · x, r² = 0.999
377__15−HETE−d8
y = 46744 · x, r² = 0.998
378__LTB4−d4
y = 88013 · x, r² = 0.999
380__PGE2−d4
y = 299026 · x, r² = 1
Peak area
Concentration (pg/μL)
