# Supplementary material for: Characterization of Lipid Profiles after Dietary Intake of Polyunsaturated Fatty Acids Using Integrated Untargeted and Targeted Lipidomics
Source: Metabolites. 2019 Oct 21;9(10):241. doi: 10.3390/metabo9100241 (PMC6836067; doi:10.3390/metabo9100241)
Supplement: Supplementary file 1 [file metabolites-09-00241-s001.zip › Supplementary Files/Supplementary Figure 2 new.pdf]

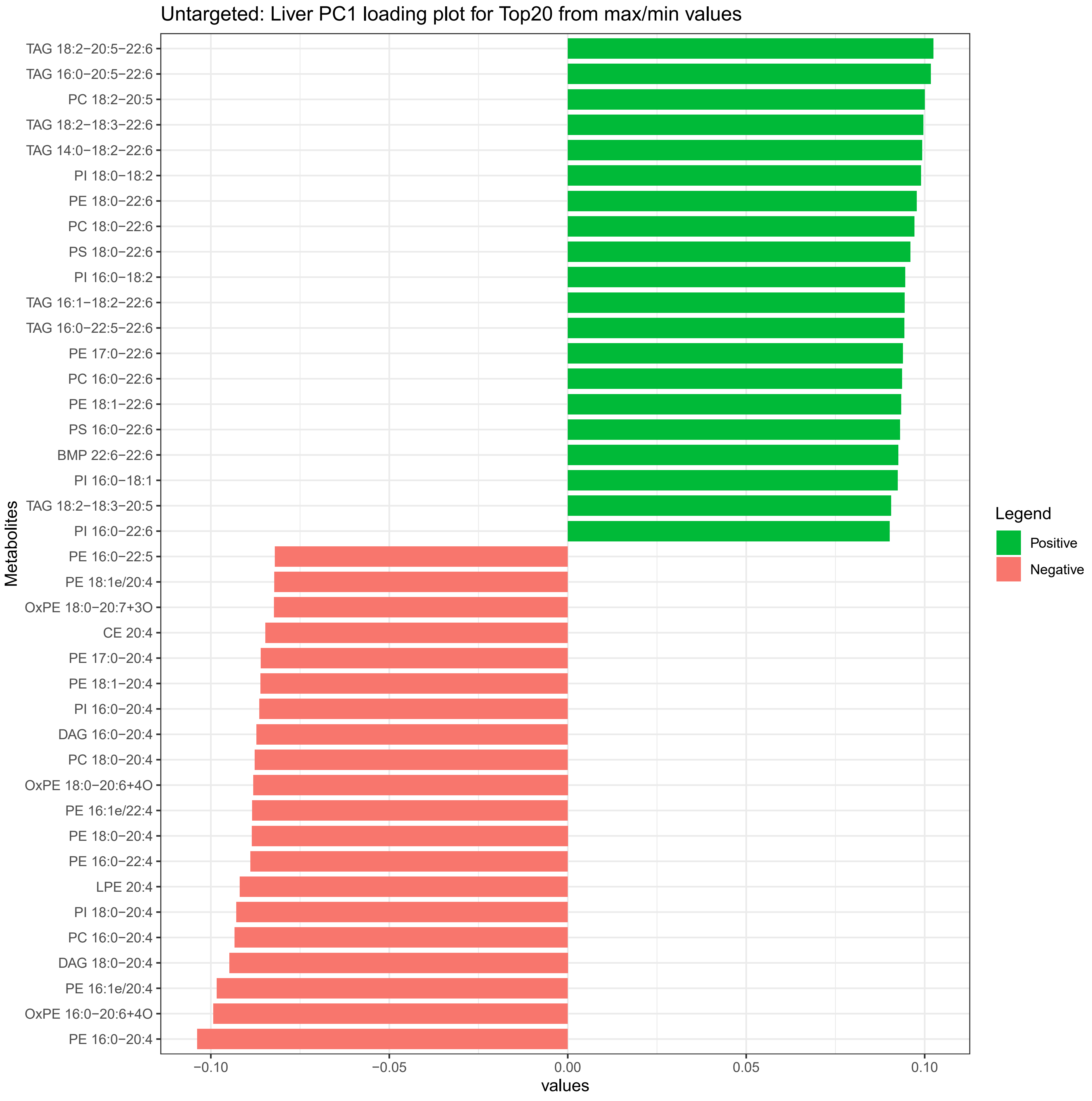
Untargeted: Liver PC1 loading plot for Top20 from max/min values
TAG 18:2−20:5−22:6
TAG 16:0−20:5−22:6
PC 18:2−20:5
TAG 18:2−18:3−22:6
TAG 14:0−18:2−22:6
PI 18:0−18:2
PE 18:0−22:6
PC 18:0−22:6
PS 18:0−22:6
PI 16:0−18:2
TAG 16:1−18:2−22:6
TAG 16:0−22:5−22:6
PE 17:0−22:6
PC 16:0−22:6
PE 18:1−22:6
PS 16:0−22:6
BMP 22:6−22:6
PI 16:0−18:1
TAG 18:2−18:3−20:5
PI 16:0−22:6
PE 16:0−22:5
PE 18:1e/20:4
OxPE 18:0−20:7+3O
CE 20:4
PE 17:0−20:4
PE 18:1−20:4
PI 16:0−20:4
DAG 16:0−20:4
PC 18:0−20:4
OxPE 18:0−20:6+4O
PE 16:1e/22:4
PE 18:0−20:4
PE 16:0−22:4
LPE 20:4
PI 18:0−20:4
PC 16:0−20:4
DAG 18:0−20:4
PE 16:1e/20:4
OxPE 16:0−20:6+4O
PE 16:0−20:4
Metabolites
values
−0.10
−0.05
0.00
0.05
0.10
Legend
Positive
Negative

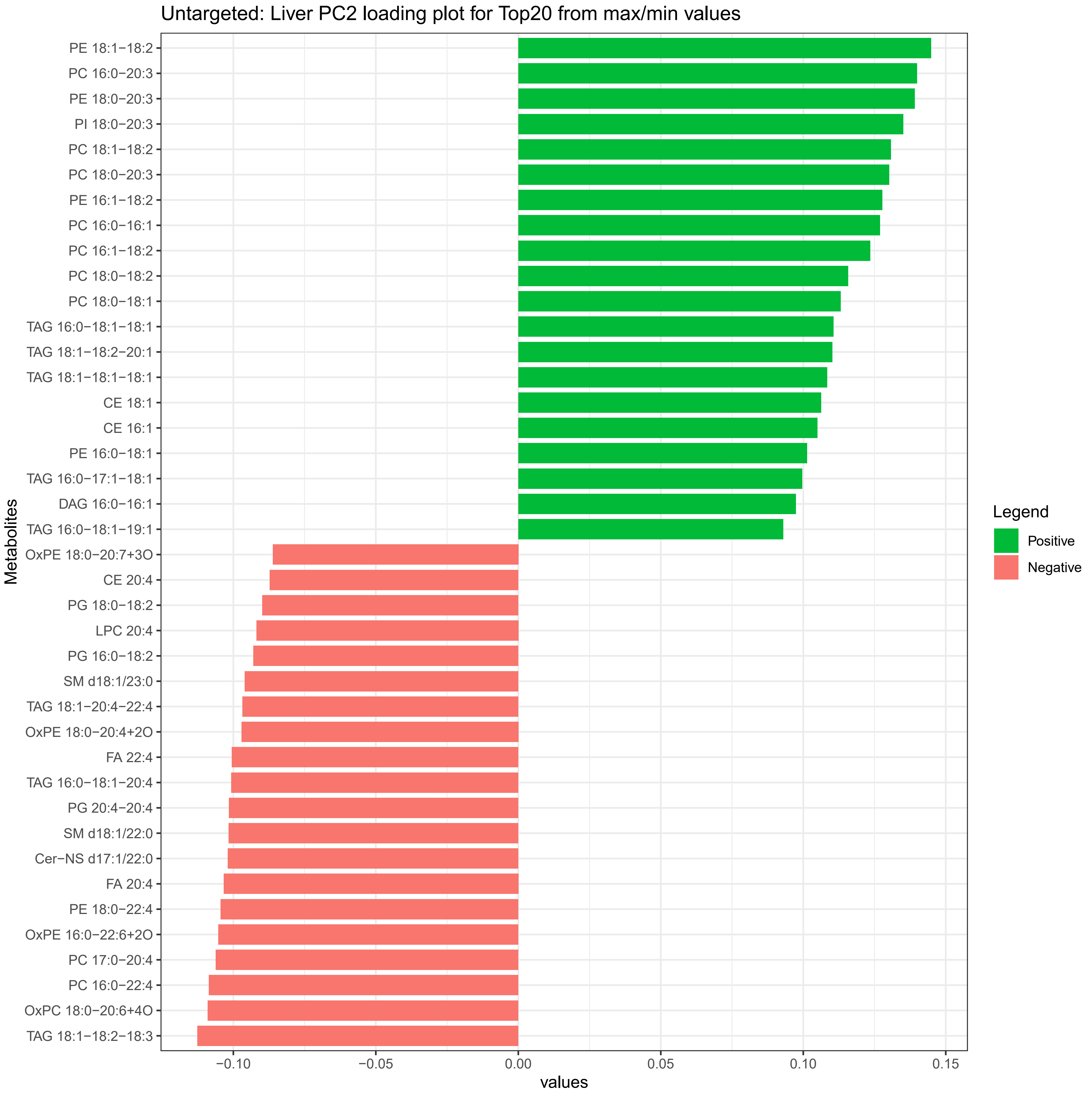
Untargeted: Liver PC2 loading plot for Top20 from max/min values
PE 18:1−18:2
PC 16:0−20:3
PE 18:0−20:3
PI 18:0−20:3
PC 18:1−18:2
PC 18:0−20:3
PE 16:1−18:2
PC 16:0−16:1
PC 16:1−18:2
PC 18:0−18:2
PC 18:0−18:1
TAG 16:0−18:1−18:1
TAG 18:1−18:2−20:1
TAG 18:1−18:1−18:1
CE 18:1
CE 16:1
PE 16:0−18:1
TAG 16:0−17:1−18:1
DAG 16:0−16:1
TAG 16:0−18:1−19:1
OxPE 18:0−20:7+3O
CE 20:4
PG 18:0−18:2
LPC 20:4
PG 16:0−18:2
SM d18:1/23:0
TAG 18:1−20:4−22:4
OxPE 18:0−20:4+2O
FA 22:4
TAG 16:0−18:1−20:4
PG 20:4−20:4
SM d18:1/22:0
Cer−NS d17:1/22:0
FA 20:4
PE 18:0−22:4
OxPE 16:0−22:6+2O
PC 17:0−20:4
PC 16:0−22:4
OxPC 18:0−20:6+4O
TAG 18:1−18:2−18:3
Metabolites
values
−0.10
−0.05
0.00
0.05
0.10
0.15
Legend
Positive
Negative

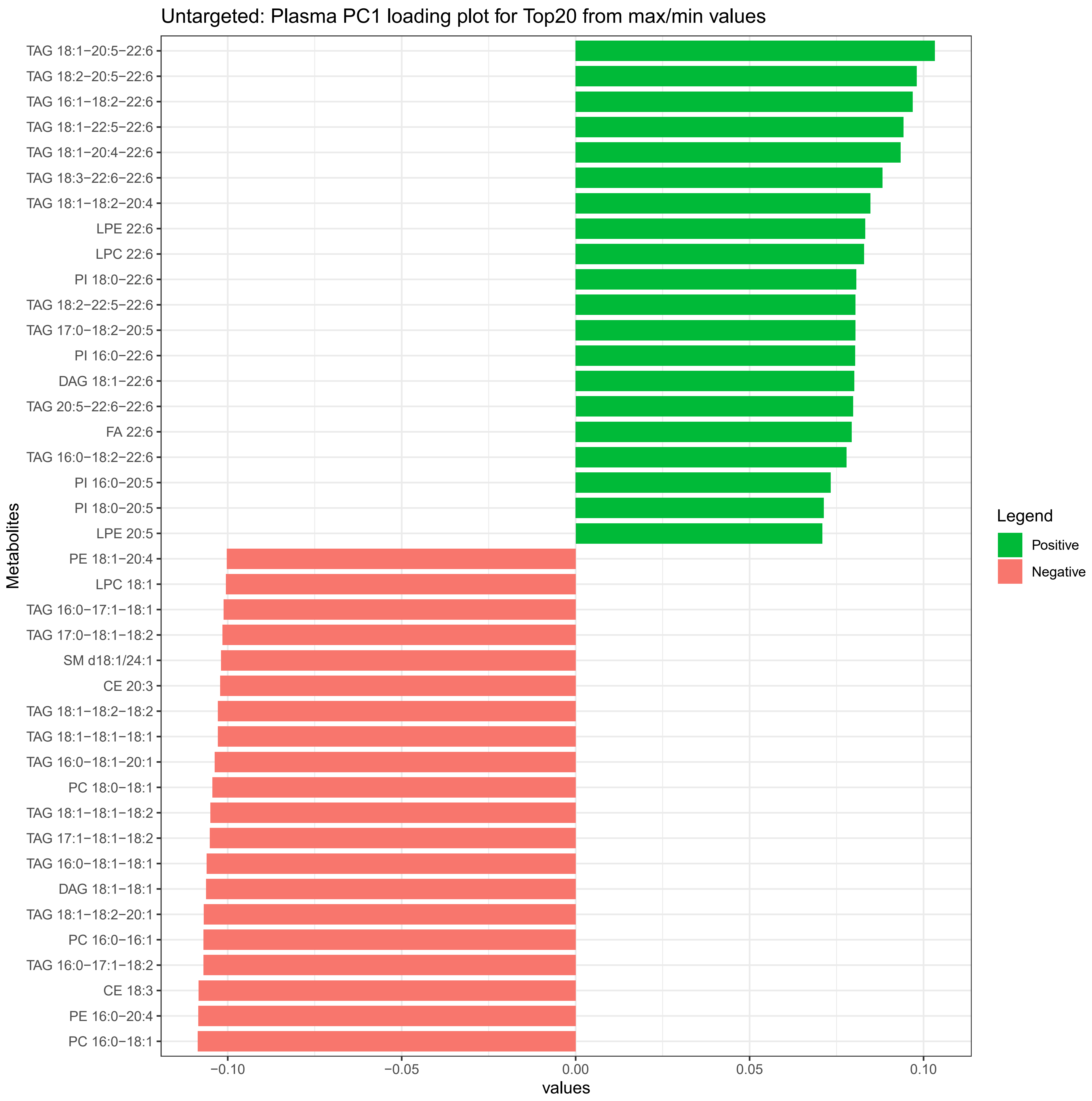
Untargeted: Plasma PC1 loading plot for Top20 from max/min values
TAG 18:1−20:5−22:6
TAG 18:2−20:5−22:6
TAG 16:1−18:2−22:6
TAG 18:1−22:5−22:6
TAG 18:1−20:4−22:6
TAG 18:3−22:6−22:6
TAG 18:1−18:2−20:4
LPE 22:6
LPC 22:6
PI 18:0−22:6
TAG 18:2−22:5−22:6
TAG 17:0−18:2−20:5
PI 16:0−22:6
DAG 18:1−22:6
TAG 20:5−22:6−22:6
FA 22:6
TAG 16:0−18:2−22:6
PI 16:0−20:5
PI 18:0−20:5
LPE 20:5
PE 18:1−20:4
LPC 18:1
TAG 16:0−17:1−18:1
TAG 17:0−18:1−18:2
SM d18:1/24:1
CE 20:3
TAG 18:1−18:2−18:2
TAG 18:1−18:1−18:1
TAG 16:0−18:1−20:1
PC 18:0−18:1
TAG 18:1−18:1−18:2
TAG 17:1−18:1−18:2
TAG 16:0−18:1−18:1
DAG 18:1−18:1
TAG 18:1−18:2−20:1
PC 16:0−16:1
TAG 16:0−17:1−18:2
CE 18:3
PE 16:0−20:4
PC 16:0−18:1
Metabolites
−0.10
−0.05
0.00
0.05
0.10
values
Legend
Positive
Negative
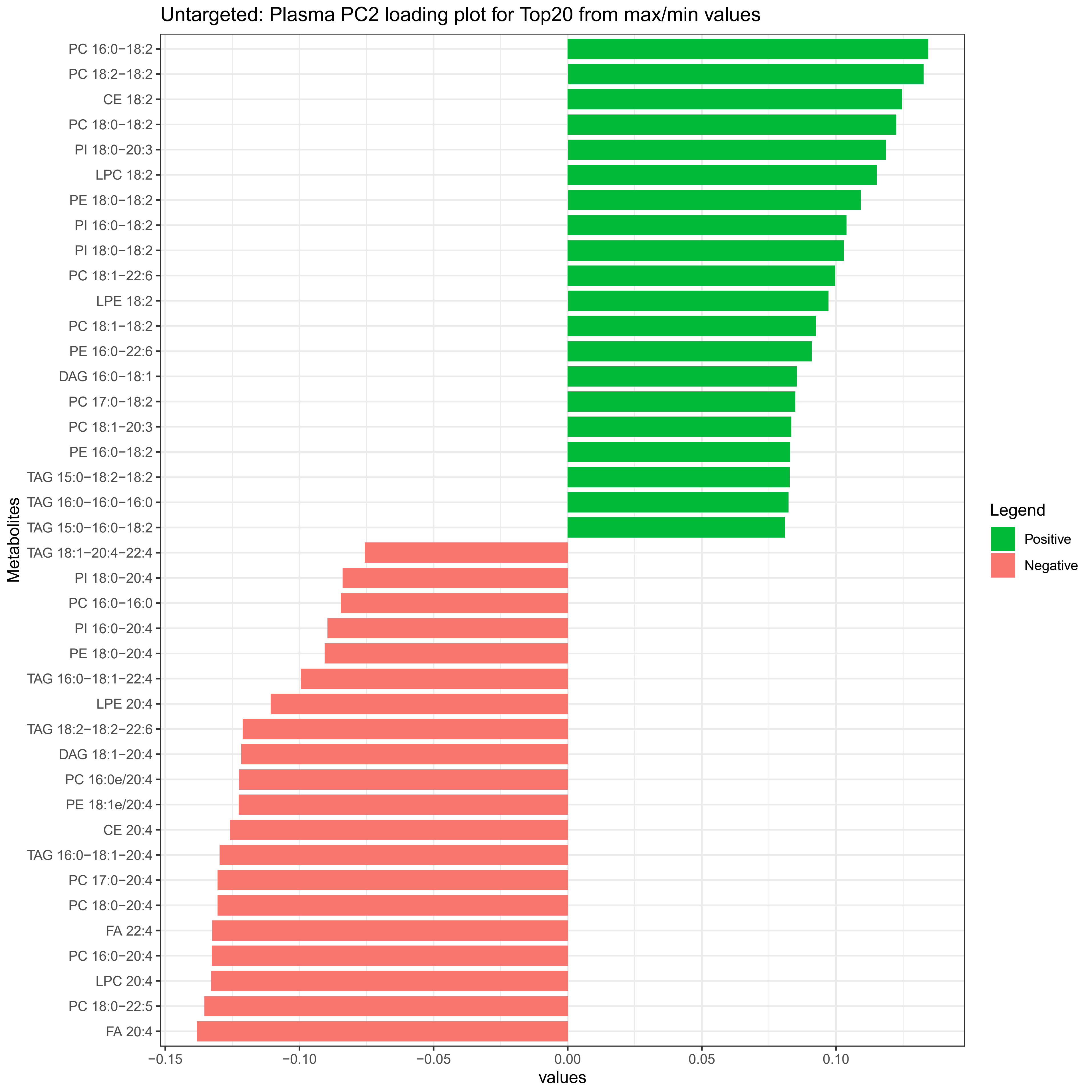
Untargeted: Plasma PC2 loading plot for Top20 from max/min values
PC 16:0−18:2
PC 18:2−18:2
CE 18:2
PC 18:0−18:2
PI 18:0−20:3
LPC 18:2
PE 18:0−18:2
PI 16:0−18:2
PI 18:0−18:2
PC 18:1−22:6
LPE 18:2
PC 18:1−18:2
PE 16:0−22:6
DAG 16:0−18:1
PC 17:0−18:2
PC 18:1−20:3
PE 16:0−18:2
TAG 15:0−18:2−18:2
TAG 16:0−16:0−16:0
TAG 15:0−16:0−18:2
TAG 18:1−20:4−22:4
PI 18:0−20:4
PC 16:0−16:0
PI 16:0−20:4
PE 18:0−20:4
TAG 16:0−18:1−22:4
LPE 20:4
TAG 18:2−18:2−22:6
DAG 18:1−20:4
PC 16:0e/20:4
PE 18:1e/20:4
CE 20:4
TAG 16:0−18:1−20:4
PC 17:0−20:4
PC 18:0−20:4
FA 22:4
PC 16:0−20:4
LPC 20:4
PC 18:0−22:5
FA 20:4
Metabolites
−0.15
−0.10
−0.05
0.00
0.05
0.10
values
Legend
Positive
Negative

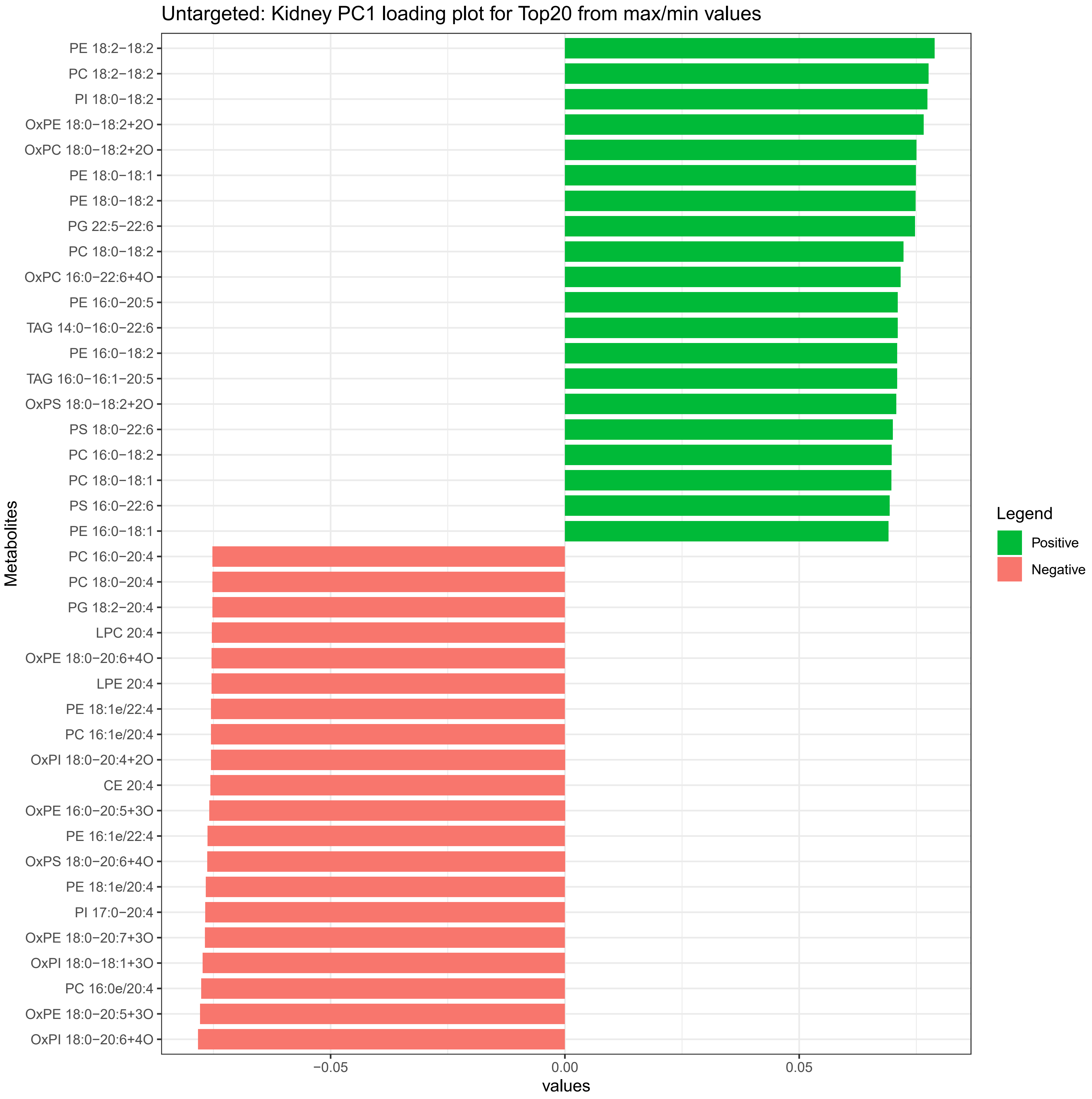
Untargeted: Kidney PC1 loading plot for Top20 from max/min values
PE 18:2−18:2
PC 18:2−18:2
PI 18:0−18:2
OxPE 18:0−18:2+2O
OxPC 18:0−18:2+2O
PE 18:0−18:1
PE 18:0−18:2
PG 22:5−22:6
PC 18:0−18:2
OxPC 16:0−22:6+4O
PE 16:0−20:5
TAG 14:0−16:0−22:6
PE 16:0−18:2
TAG 16:0−16:1−20:5
OxPS 18:0−18:2+2O
PS 18:0−22:6
PC 16:0−18:2
PC 18:0−18:1
PS 16:0−22:6
PE 16:0−18:1
PC 16:0−20:4
PC 18:0−20:4
PG 18:2−20:4
LPC 20:4
OxPE 18:0−20:6+4O
LPE 20:4
PE 18:1e/22:4
PC 16:1e/20:4
OxPI 18:0−20:4+2O
CE 20:4
OxPE 16:0−20:5+3O
PE 16:1e/22:4
OxPS 18:0−20:6+4O
PE 18:1e/20:4
PI 17:0−20:4
OxPE 18:0−20:7+3O
OxPI 18:0−18:1+3O
PC 16:0e/20:4
OxPE 18:0−20:5+3O
OxPI 18:0−20:6+4O
Metabolites
−0.05
0.00
0.05
values
Legend
Positive
Negative

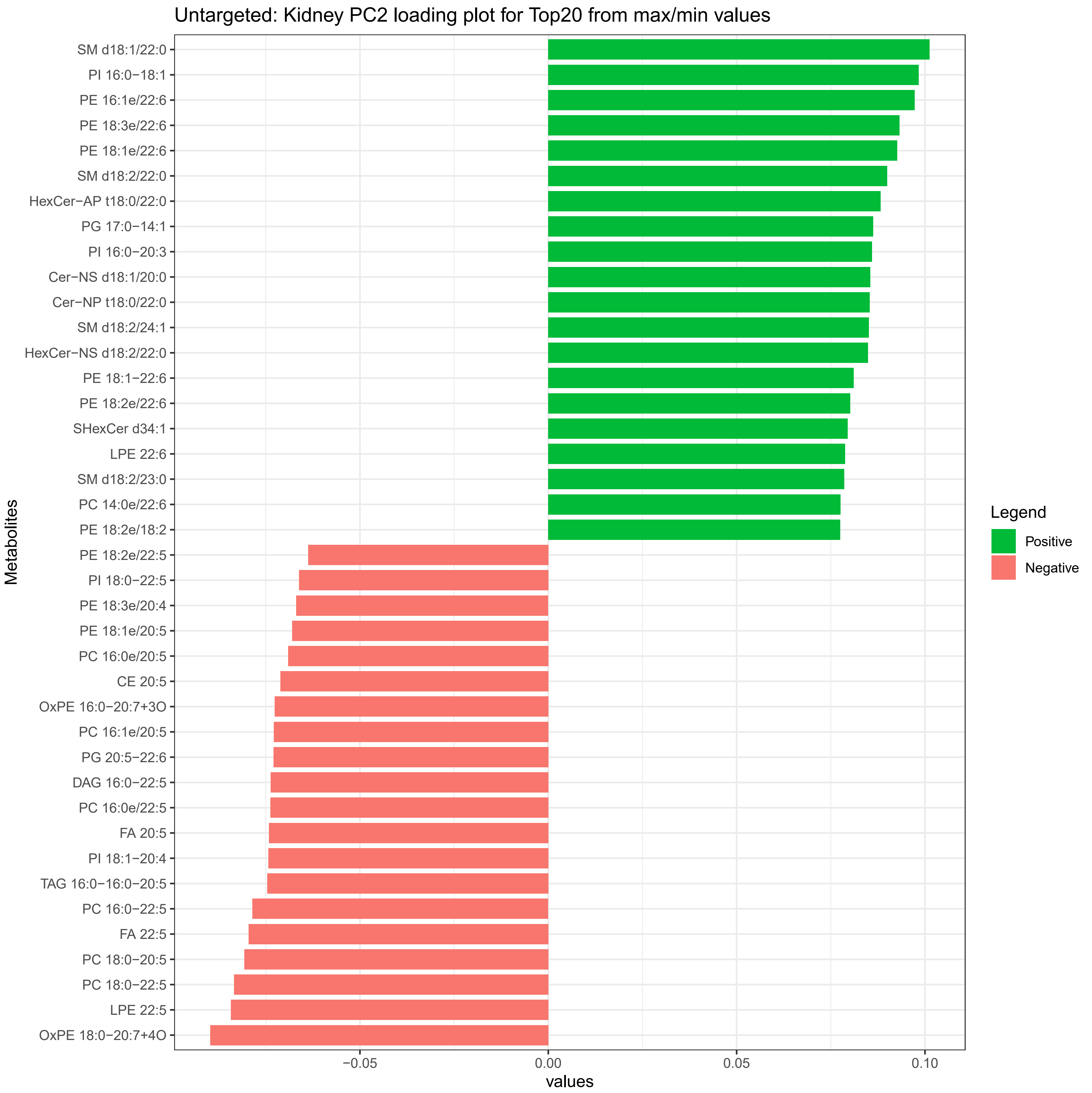
Untargeted: Kidney PC2 loading plot for Top20 from max/min values
SM d18:1/22:0
PI 16:0−18:1
PE 16:1e/22:6
PE 18:3e/22:6
PE 18:1e/22:6
SM d18:2/22:0
HexCer−AP t18:0/22:0
PG 17:0−14:1
PI 16:0−20:3
Cer−NS d18:1/20:0
Cer−NP t18:0/22:0
SM d18:2/24:1
HexCer−NS d18:2/22:0
PE 18:1−22:6
PE 18:2e/22:6
SHexCer d34:1
LPE 22:6
SM d18:2/23:0
PC 14:0e/22:6
PE 18:2e/18:2
PE 18:2e/22:5
PI 18:0−22:5
PE 18:3e/20:4
PE 18:1e/20:5
PC 16:0e/20:5
CE 20:5
OxPE 16:0−20:7+3O
PC 16:1e/20:5
PG 20:5−22:6
DAG 16:0−22:5
PC 16:0e/22:5
FA 20:5
PI 18:1−20:4
TAG 16:0−16:0−20:5
PC 16:0−22:5
FA 22:5
PC 18:0−20:5
PC 18:0−22:5
LPE 22:5
OxPE 18:0−20:7+4O
Metabolites
−0.05
0.00
0.05
0.10
values
Legend
Positive
Negative

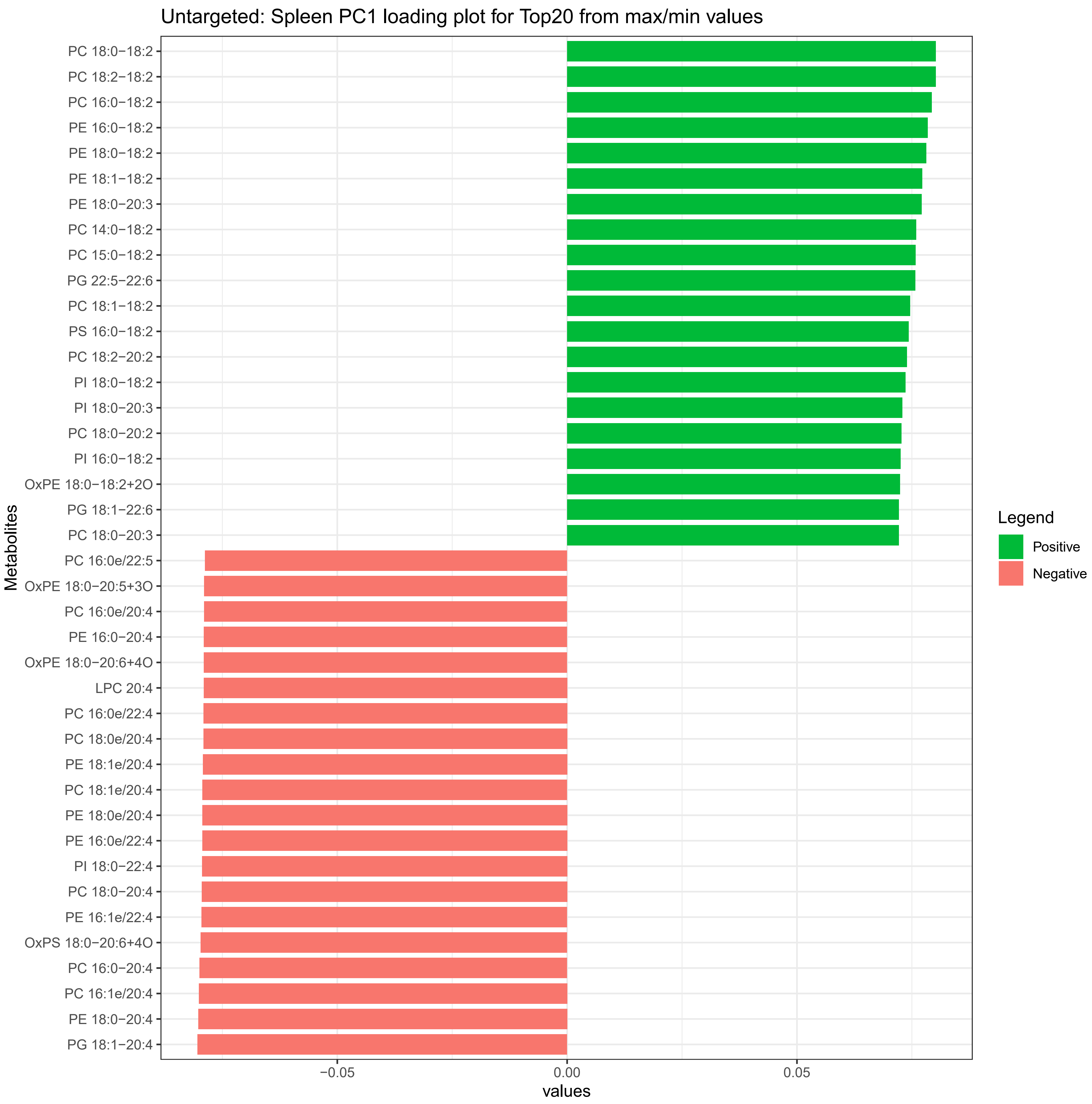

Untargeted: Spleen PC1 loading plot for Top20 from max/min values
Metabolites
values
Legend
Positive
Negative
PC 18:0−18:2
PC 18:2−18:2
PC 16:0−18:2
PE 16:0−18:2
PE 18:0−18:2
PE 18:1−18:2
PE 18:0−20:3
PC 14:0−18:2
PC 15:0−18:2
PG 22:5−22:6
PC 18:1−18:2
PS 16:0−18:2
PC 18:2−20:2
PI 18:0−18:2
PI 18:0−20:3
PC 18:0−20:2
PI 16:0−18:2
OxPE 18:0−18:2+2O
PG 18:1−22:6
PC 18:0−20:3
PC 16:0e/22:5
OxPE 18:0−20:5+3O
PC 16:0e/20:4
PE 16:0−20:4
OxPE 18:0−20:6+4O
LPC 20:4
PC 16:0e/22:4
PC 18:0e/20:4
PE 18:1e/20:4
PC 18:1e/20:4
PE 18:0e/20:4
PE 16:0e/22:4
PI 18:0−22:4
PC 18:0−20:4
PE 16:1e/22:4
OxPS 18:0−20:6+4O
PC 16:0−20:4
PC 16:1e/20:4
PE 18:0−20:4
PG 18:1−20:4
−0.05
0.00
0.05

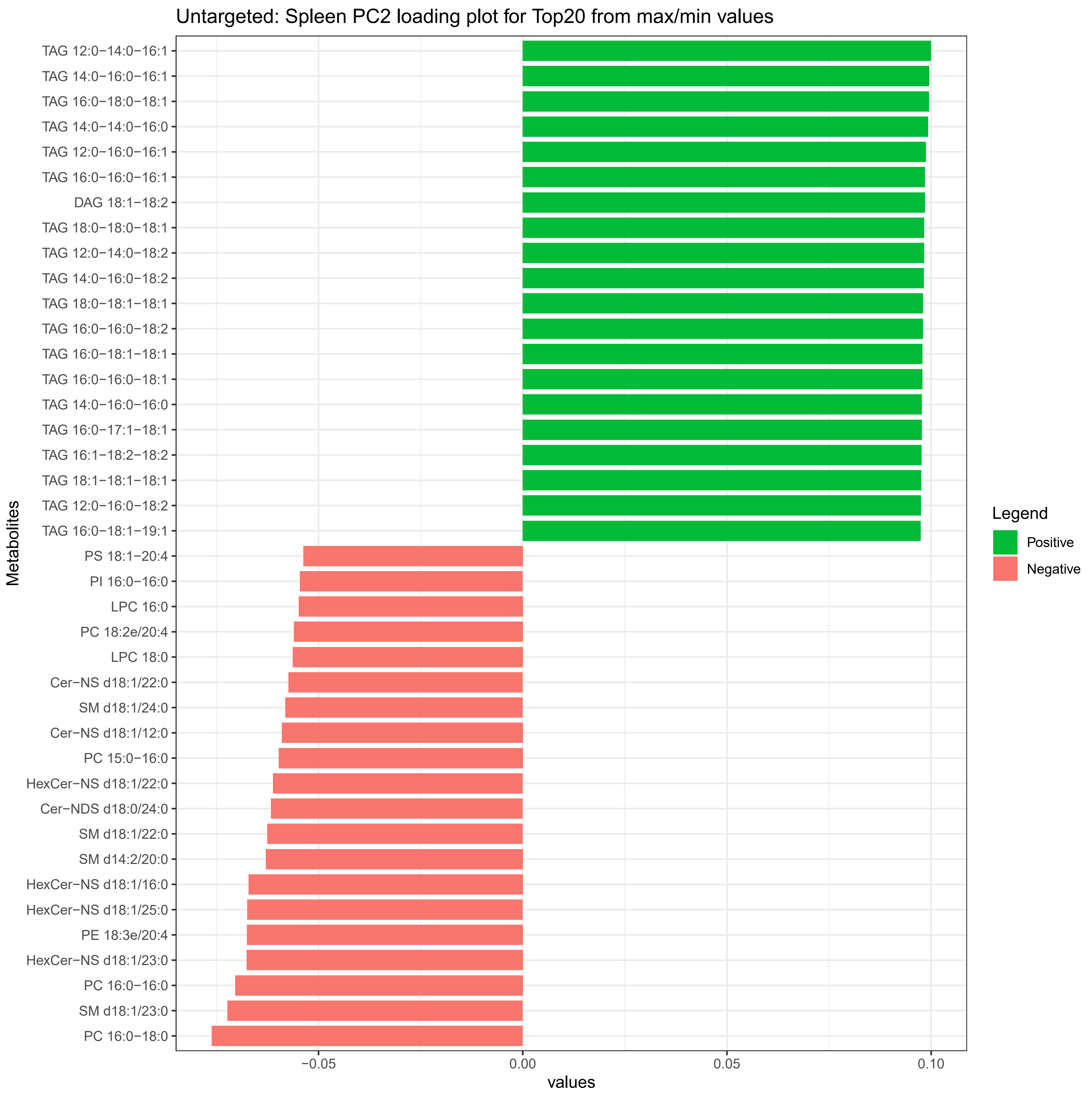

Untargeted: Spleen PC2 loading plot for Top20 from max/min values
Metabolites
values
Legend
Positive
Negative
TAG 12:0−14:0−16:1
TAG 14:0−16:0−16:1
TAG 16:0−18:0−18:1
TAG 14:0−14:0−16:0
TAG 12:0−16:0−16:1
TAG 16:0−16:0−16:1
DAG 18:1−18:2
TAG 18:0−18:0−18:1
TAG 12:0−14:0−18:2
TAG 14:0−16:0−18:2
TAG 18:0−18:1−18:1
TAG 16:0−16:0−18:2
TAG 16:0−18:1−18:1
TAG 16:0−16:0−18:1
TAG 14:0−16:0−16:0
TAG 16:0−17:1−18:1
TAG 16:1−18:2−18:2
TAG 18:1−18:1−18:1
TAG 12:0−16:0−18:2
TAG 16:0−18:1−19:1
PS 18:1−20:4
PI 16:0−16:0
LPC 16:0
PC 18:2e/20:4
LPC 18:0
Cer−NS d18:1/22:0
SM d18:1/24:0
Cer−NS d18:1/12:0
PC 15:0−16:0
HexCer−NS d18:1/22:0
Cer−NDS d18:0/24:0
SM d18:1/22:0
SM d14:2/20:0
HexCer−NS d18:1/16:0
HexCer−NS d18:1/25:0
PE 18:3e/20:4
HexCer−NS d18:1/23:0
PC 16:0−16:0
SM d18:1/23:0
PC 16:0−18:0
−0.05
0.00
0.05
0.10

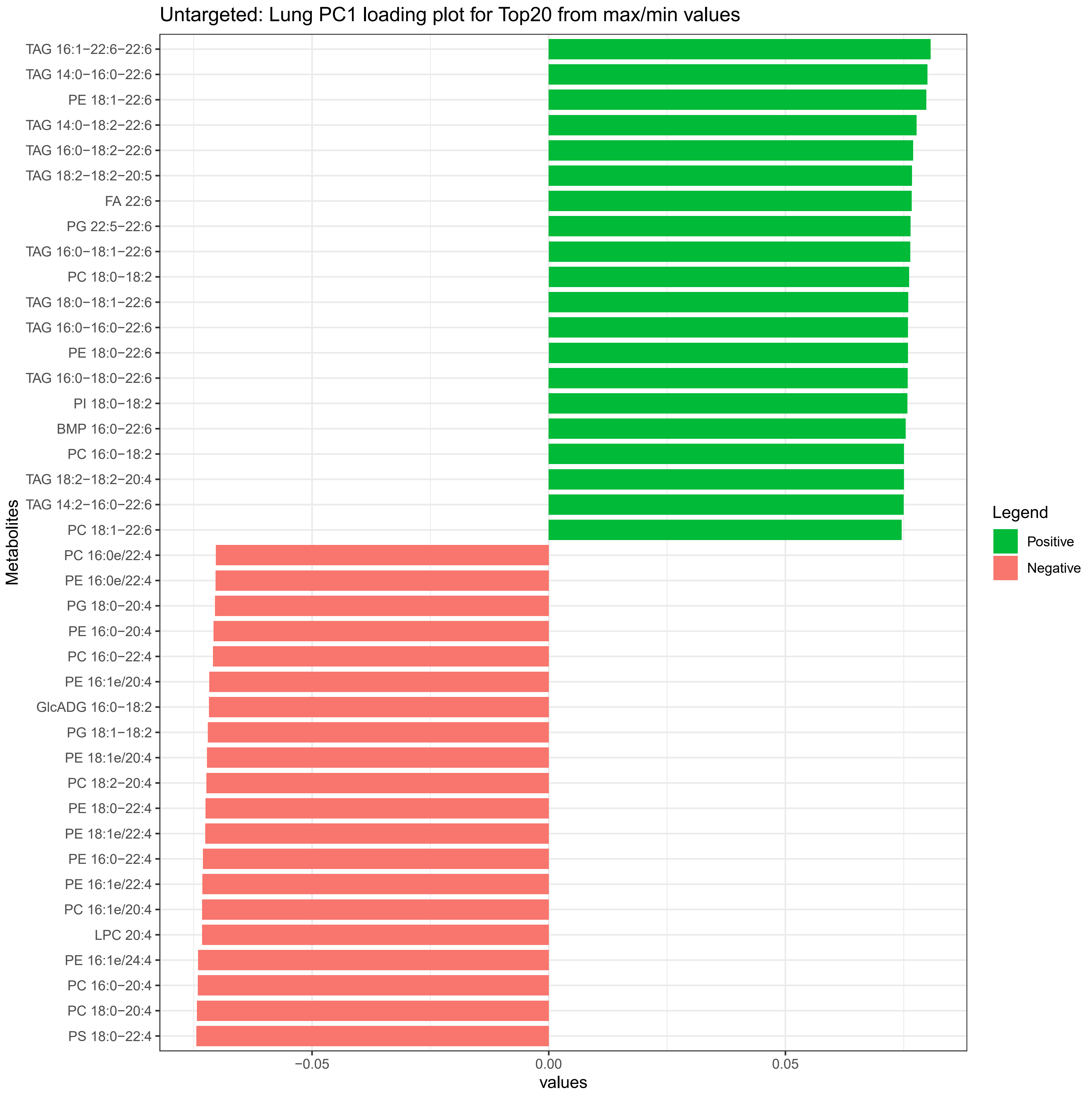

Untargeted: Lung PC1 loading plot for Top20 from max/min values
TAG 16:1−22:6−22:6
TAG 14:0−16:0−22:6
PE 18:1−22:6
TAG 14:0−18:2−22:6
TAG 16:0−18:2−22:6
TAG 18:2−18:2−20:5
FA 22:6
PG 22:5−22:6
TAG 16:0−18:1−22:6
PC 18:0−18:2
TAG 18:0−18:1−22:6
TAG 16:0−16:0−22:6
PE 18:0−22:6
TAG 16:0−18:0−22:6
PI 18:0−18:2
BMP 16:0−22:6
PC 16:0−18:2
TAG 18:2−18:2−20:4
TAG 14:2−16:0−22:6
PC 18:1−22:6
PC 16:0e/22:4
PE 16:0e/22:4
PG 18:0−20:4
PE 16:0−20:4
PC 16:0−22:4
PE 16:1e/20:4
GlcADG 16:0−18:2
PG 18:1−18:2
PE 18:1e/20:4
PC 18:2−20:4
PE 18:0−22:4
PE 18:1e/22:4
PE 16:0−22:4
PE 16:1e/22:4
PC 16:1e/20:4
LPC 20:4
PE 16:1e/24:4
PC 16:0−20:4
PC 18:0−20:4
PS 18:0−22:4
Metabolites
−0.05
0.00
0.05
values
Legend
Positive
Negative

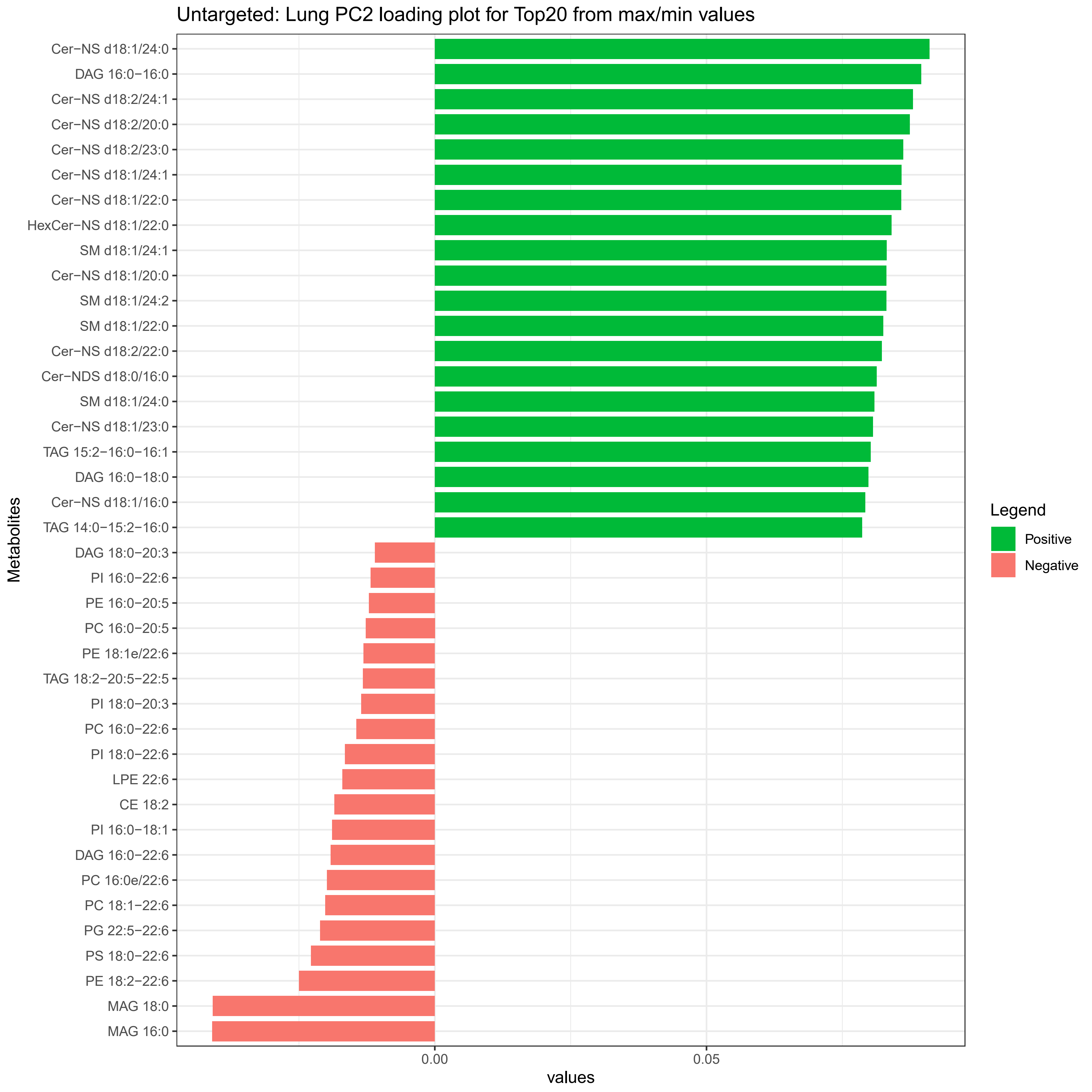

Untargeted: Lung PC2 loading plot for Top20 from max/min values
Cer−NS d18:1/24:0
DAG 16:0−16:0
Cer−NS d18:2/24:1
Cer−NS d18:2/20:0
Cer−NS d18:2/23:0
Cer−NS d18:1/24:1
Cer−NS d18:1/22:0
HexCer−NS d18:1/22:0
SM d18:1/24:1
Cer−NS d18:1/20:0
SM d18:1/24:2
SM d18:1/22:0
Cer−NS d18:2/22:0
Cer−NDS d18:0/16:0
SM d18:1/24:0
Cer−NS d18:1/23:0
TAG 15:2−16:0−16:1
DAG 16:0−18:0
Cer−NS d18:1/16:0
TAG 14:0−15:2−16:0
DAG 18:0−20:3
PI 16:0−22:6
PE 16:0−20:5
PC 16:0−20:5
PE 18:1e/22:6
TAG 18:2−20:5−22:5
PI 18:0−20:3
PC 16:0−22:6
PI 18:0−22:6
LPE 22:6
CE 18:2
PI 16:0−18:1
DAG 16:0−22:6
PC 16:0e/22:6
PC 18:1−22:6
PG 22:5−22:6
PS 18:0−22:6
PE 18:2−22:6
MAG 18:0
MAG 16:0
Metabolites
0.00
0.05
values
Legend
Positive
Negative

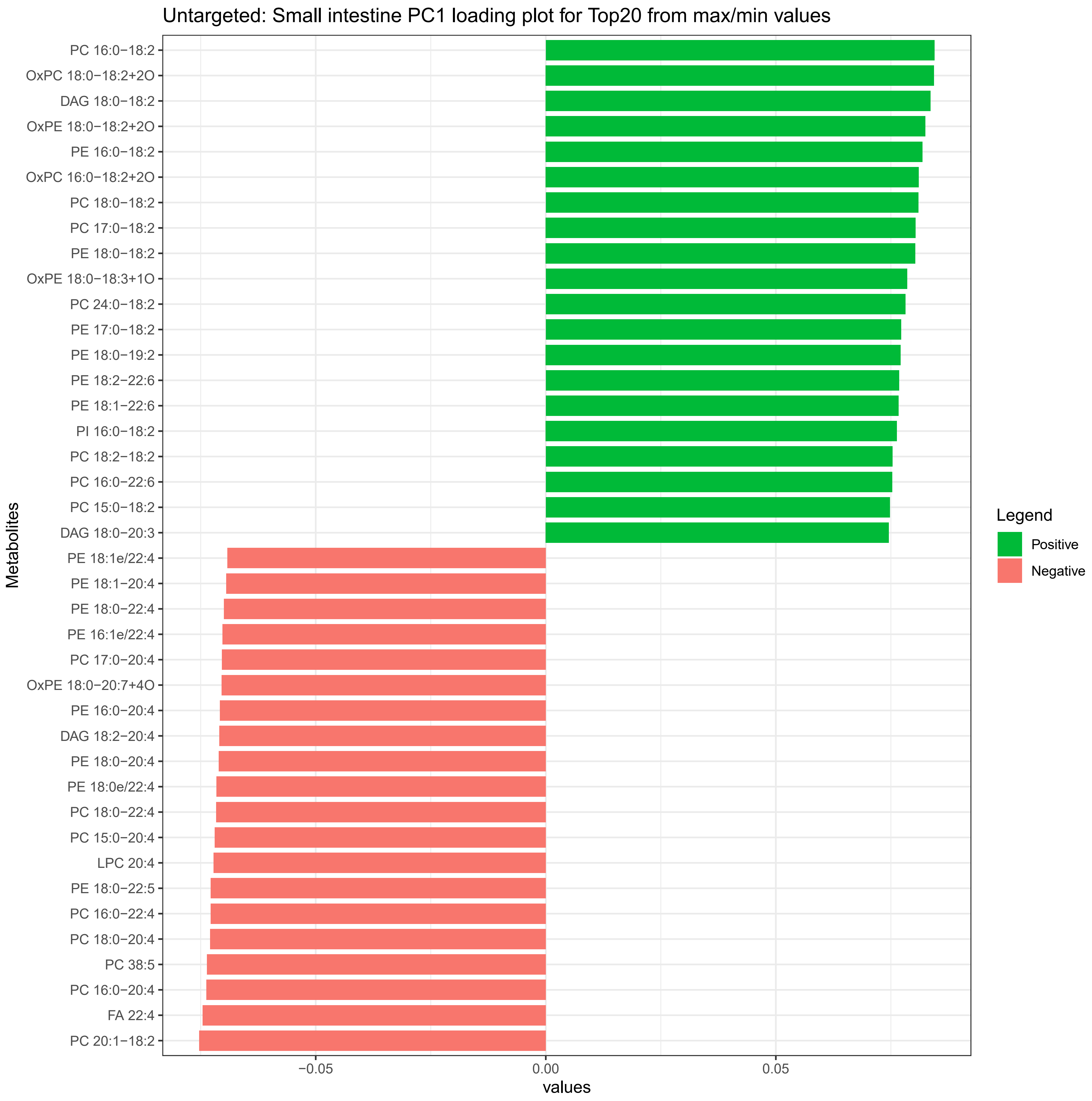

Untargeted: Small intestine PC1 loading plot for Top20 from max/min values
Metabolites
values
Legend
Positive
Negative
PC 16:0−18:2
OxPC 18:0−18:2+2O
DAG 18:0−18:2
OxPE 18:0−18:2+2O
PE 16:0−18:2
OxPC 16:0−18:2+2O
PC 18:0−18:2
PC 17:0−18:2
PE 18:0−18:2
OxPE 18:0−18:3+1O
PC 24:0−18:2
PE 17:0−18:2
PE 18:0−19:2
PE 18:2−22:6
PE 18:1−22:6
PI 16:0−18:2
PC 18:2−18:2
PC 16:0−22:6
PC 15:0−18:2
DAG 18:0−20:3
PE 18:1e/22:4
PE 18:1−20:4
PE 18:0−22:4
PE 16:1e/22:4
PC 17:0−20:4
OxPE 18:0−20:7+4O
PE 16:0−20:4
DAG 18:2−20:4
PE 18:0−20:4
PE 18:0e/22:4
PC 18:0−22:4
PC 15:0−20:4
LPC 20:4
PE 18:0−22:5
PC 16:0−22:4
PC 18:0−20:4
PC 38:5
PC 16:0−20:4
FA 22:4
PC 20:1−18:2
−0.05
0.00
0.05

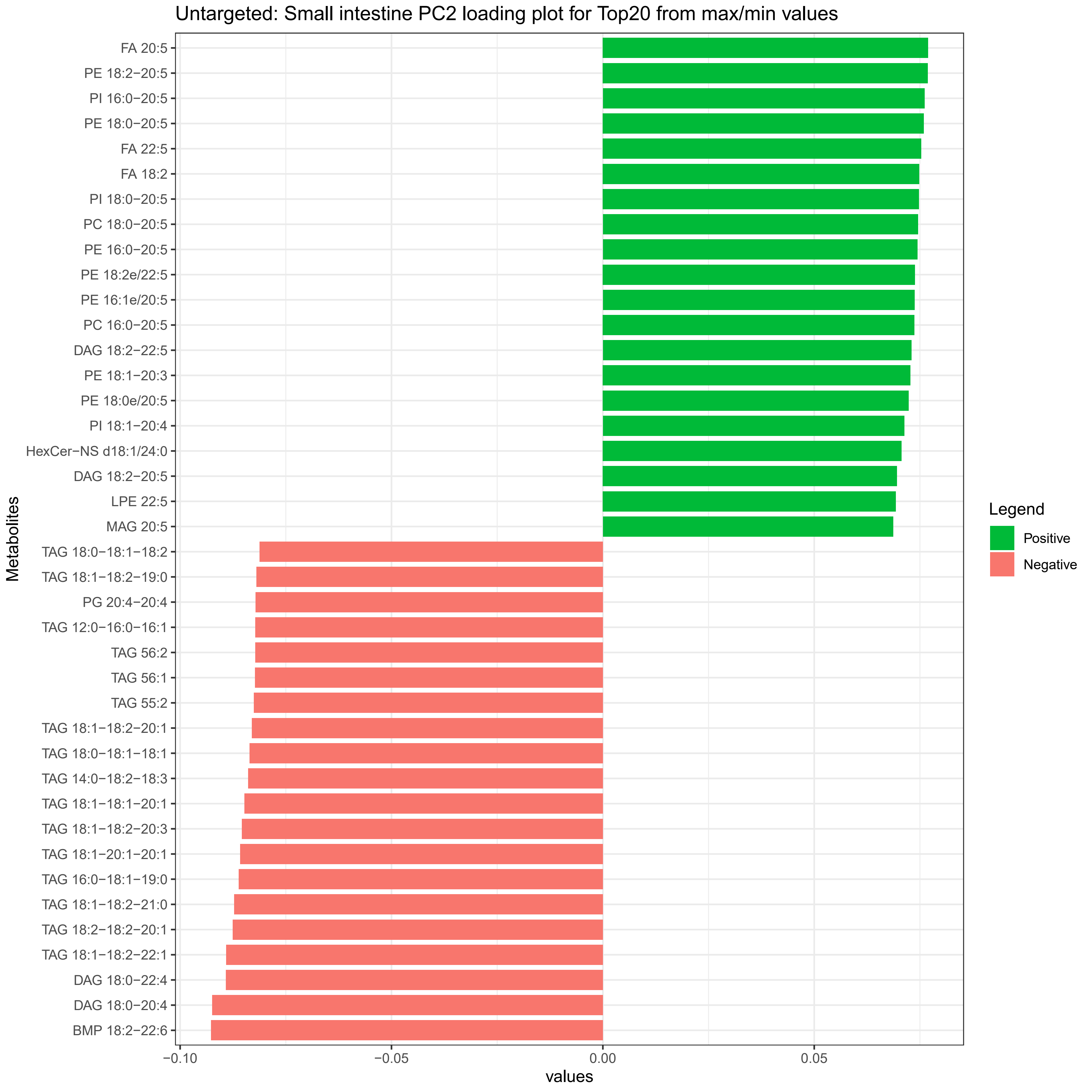

Untargeted: Small intestine PC2 loading plot for Top20 from max/min values
Metabolites
values
Legend
Positive
Negative
FA 20:5
PE 18:2−20:5
PI 16:0−20:5
PE 18:0−20:5
FA 22:5
FA 18:2
PI 18:0−20:5
PC 18:0−20:5
PE 16:0−20:5
PE 18:2e/22:5
PE 16:1e/20:5
PC 16:0−20:5
DAG 18:2−22:5
PE 18:1−20:3
PE 18:0e/20:5
PI 18:1−20:4
HexCer−NS d18:1/24:0
DAG 18:2−20:5
LPE 22:5
MAG 20:5
TAG 18:0−18:1−18:2
TAG 18:1−18:2−19:0
PG 20:4−20:4
TAG 12:0−16:0−16:1
TAG 56:2
TAG 56:1
TAG 55:2
TAG 18:1−18:2−20:1
TAG 18:0−18:1−18:1
TAG 14:0−18:2−18:3
TAG 18:1−18:1−20:1
TAG 18:1−18:2−20:3
TAG 18:1−20:1−20:1
TAG 16:0−18:1−19:0
TAG 18:1−18:2−21:0
TAG 18:2−18:2−20:1
TAG 18:1−18:2−22:1
DAG 18:0−22:4
DAG 18:0−20:4
BMP 18:2−22:6
−0.10
−0.05
0.00
0.05

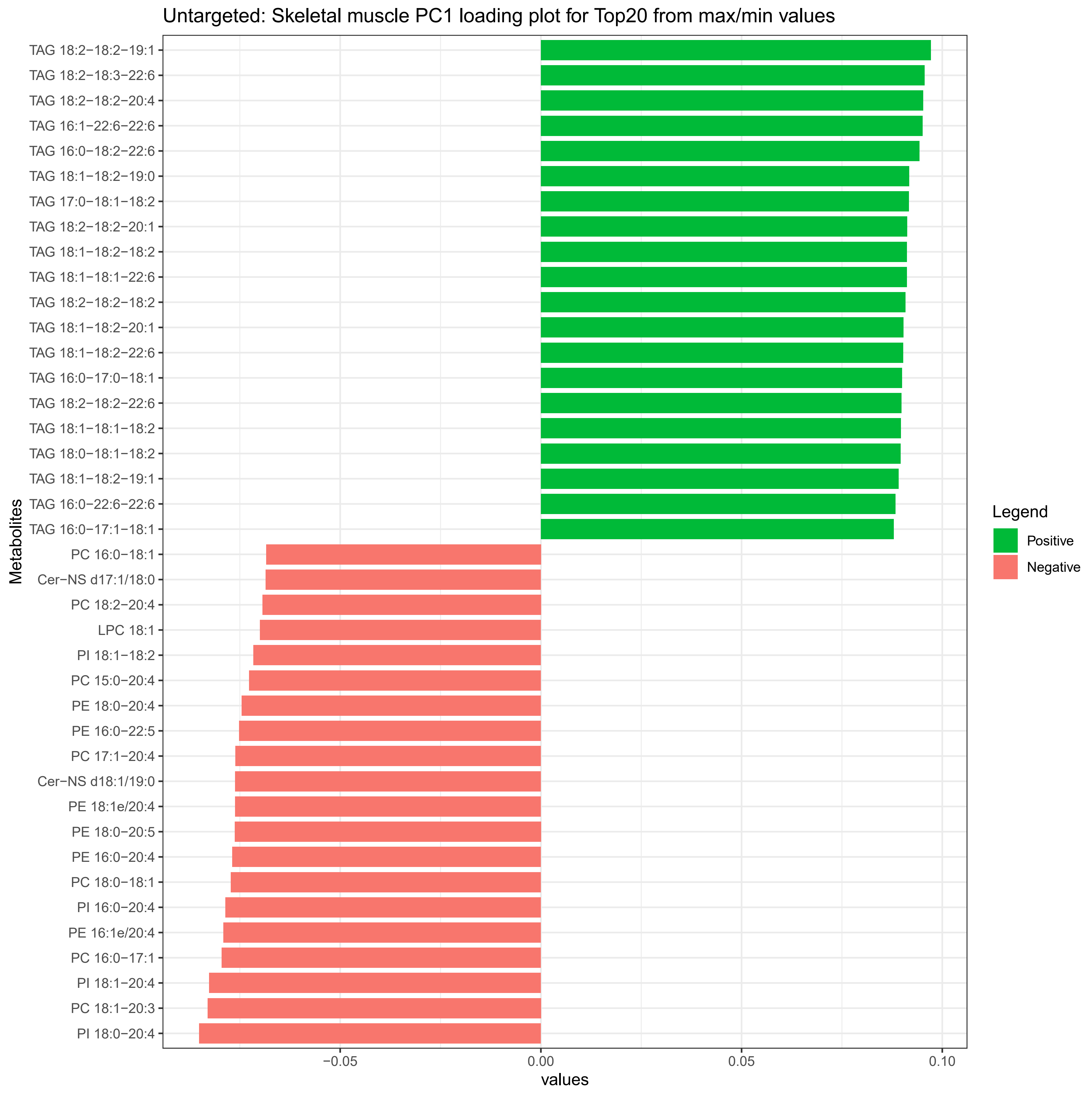
Untargeted: Skeletal muscle PC1 loading plot for Top20 from max/min values
TAG 18:2−18:2−19:1
TAG 18:2−18:3−22:6
TAG 18:2−18:2−20:4
TAG 16:1−22:6−22:6
TAG 16:0−18:2−22:6
TAG 18:1−18:2−19:0
TAG 17:0−18:1−18:2
TAG 18:2−18:2−20:1
TAG 18:1−18:2−18:2
TAG 18:1−18:1−22:6
TAG 18:2−18:2−18:2
TAG 18:1−18:2−20:1
TAG 18:1−18:2−22:6
TAG 16:0−17:0−18:1
TAG 18:2−18:2−22:6
TAG 18:1−18:1−18:2
TAG 18:0−18:1−18:2
TAG 18:1−18:2−19:1
TAG 16:0−22:6−22:6
TAG 16:0−17:1−18:1
PC 16:0−18:1
Cer−NS d17:1/18:0
PC 18:2−20:4
LPC 18:1
PI 18:1−18:2
PC 15:0−20:4
PE 18:0−20:4
PE 16:0−22:5
PC 17:1−20:4
Cer−NS d18:1/19:0
PE 18:1e/20:4
PE 18:0−20:5
PE 16:0−20:4
PC 18:0−18:1
PI 16:0−20:4
PE 16:1e/20:4
PC 16:0−17:1
PI 18:1−20:4
PC 18:1−20:3
PI 18:0−20:4
Metabolites
−0.05
0.00
0.05
0.10
values
Legend
Positive
Negative

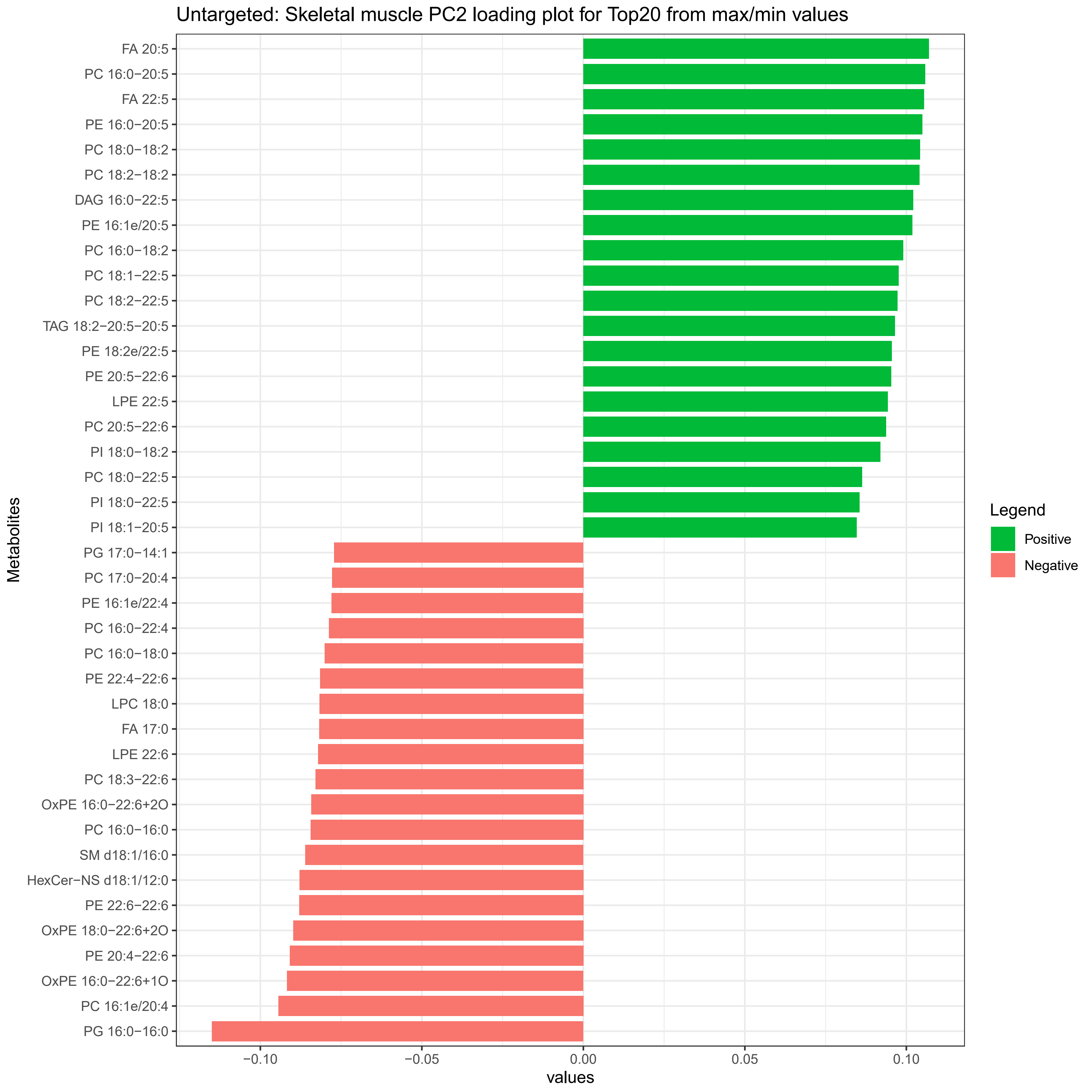
Untargeted: Skeletal muscle PC2 loading plot for Top20 from max/min values
FA 20:5
PC 16:0−20:5
FA 22:5
PE 16:0−20:5
PC 18:0−18:2
PC 18:2−18:2
DAG 16:0−22:5
PE 16:1e/20:5
PC 16:0−18:2
PC 18:1−22:5
PC 18:2−22:5
TAG 18:2−20:5−20:5
PE 18:2e/22:5
PE 20:5−22:6
LPE 22:5
PC 20:5−22:6
PI 18:0−18:2
PC 18:0−22:5
PI 18:0−22:5
PI 18:1−20:5
PG 17:0−14:1
PC 17:0−20:4
PE 16:1e/22:4
PC 16:0−22:4
PC 16:0−18:0
PE 22:4−22:6
LPC 18:0
FA 17:0
LPE 22:6
PC 18:3−22:6
OxPE 16:0−22:6+2O
PC 16:0−16:0
SM d18:1/16:0
HexCer−NS d18:1/12:0
PE 22:6−22:6
OxPE 18:0−22:6+2O
PE 20:4−22:6
OxPE 16:0−22:6+1O
PC 16:1e/20:4
PG 16:0−16:0
Metabolites
−0.10
−0.05
0.00
0.05
0.10
values
Legend
Positive
Negative

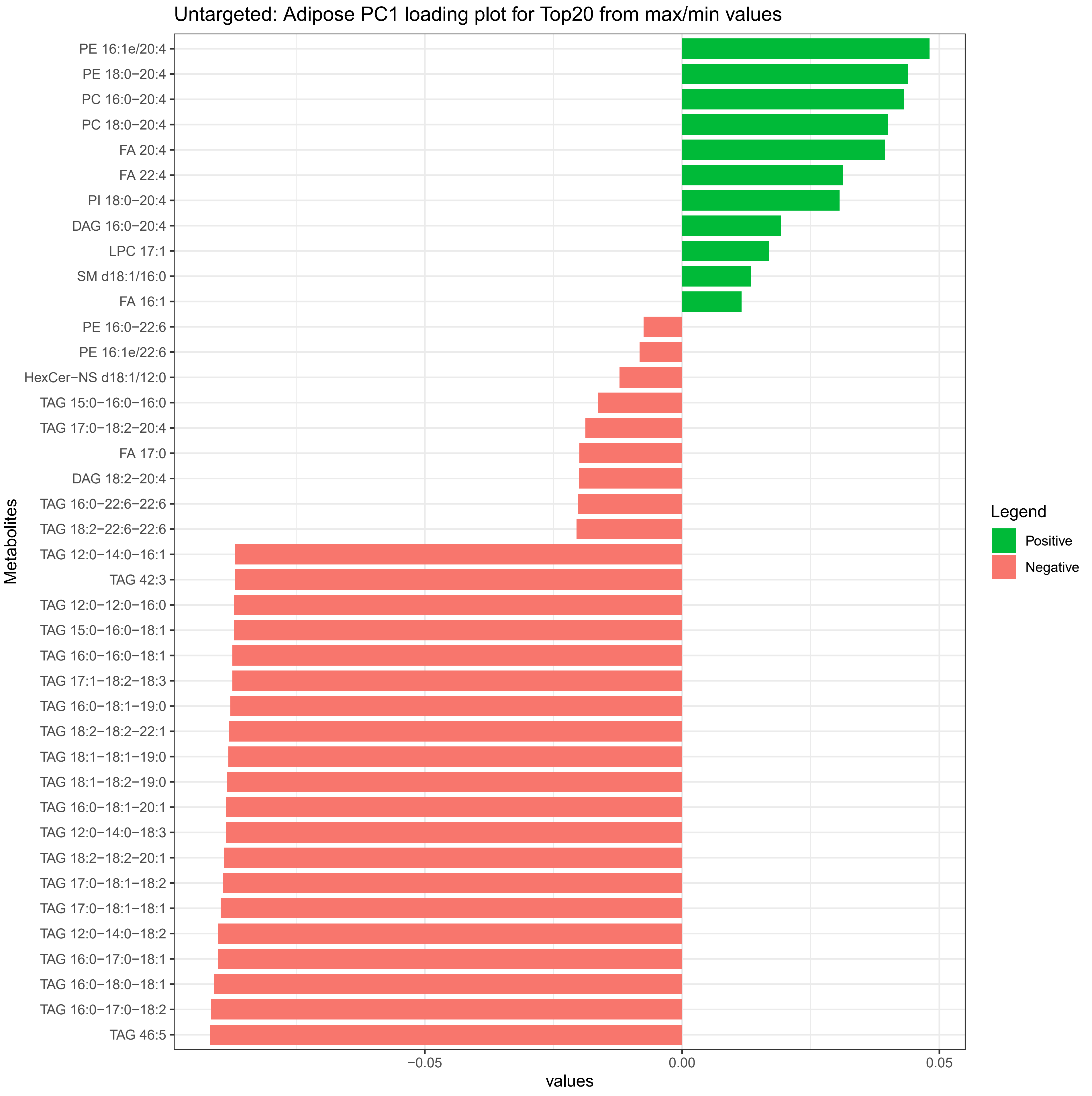

Untargeted: Adipose PC1 loading plot for Top20 from max/min values
PE 16:1e/20:4
PE 18:0−20:4
PC 16:0−20:4
PC 18:0−20:4
FA 20:4
FA 22:4
PI 18:0−20:4
DAG 16:0−20:4
LPC 17:1
SM d18:1/16:0
FA 16:1
PE 16:0−22:6
PE 16:1e/22:6
HexCer−NS d18:1/12:0
TAG 15:0−16:0−16:0
TAG 17:0−18:2−20:4
FA 17:0
DAG 18:2−20:4
TAG 16:0−22:6−22:6
TAG 18:2−22:6−22:6
TAG 12:0−14:0−16:1
TAG 42:3
TAG 12:0−12:0−16:0
TAG 15:0−16:0−18:1
TAG 16:0−16:0−18:1
TAG 17:1−18:2−18:3
TAG 16:0−18:1−19:0
TAG 18:2−18:2−22:1
TAG 18:1−18:1−19:0
TAG 18:1−18:2−19:0
TAG 16:0−18:1−20:1
TAG 12:0−14:0−18:3
TAG 18:2−18:2−20:1
TAG 17:0−18:1−18:2
TAG 17:0−18:1−18:1
TAG 12:0−14:0−18:2
TAG 16:0−17:0−18:1
TAG 16:0−18:0−18:1
TAG 16:0−17:0−18:2
TAG 46:5
Metabolites
−0.05
0.00
0.05
values
Legend
Positive
Negative

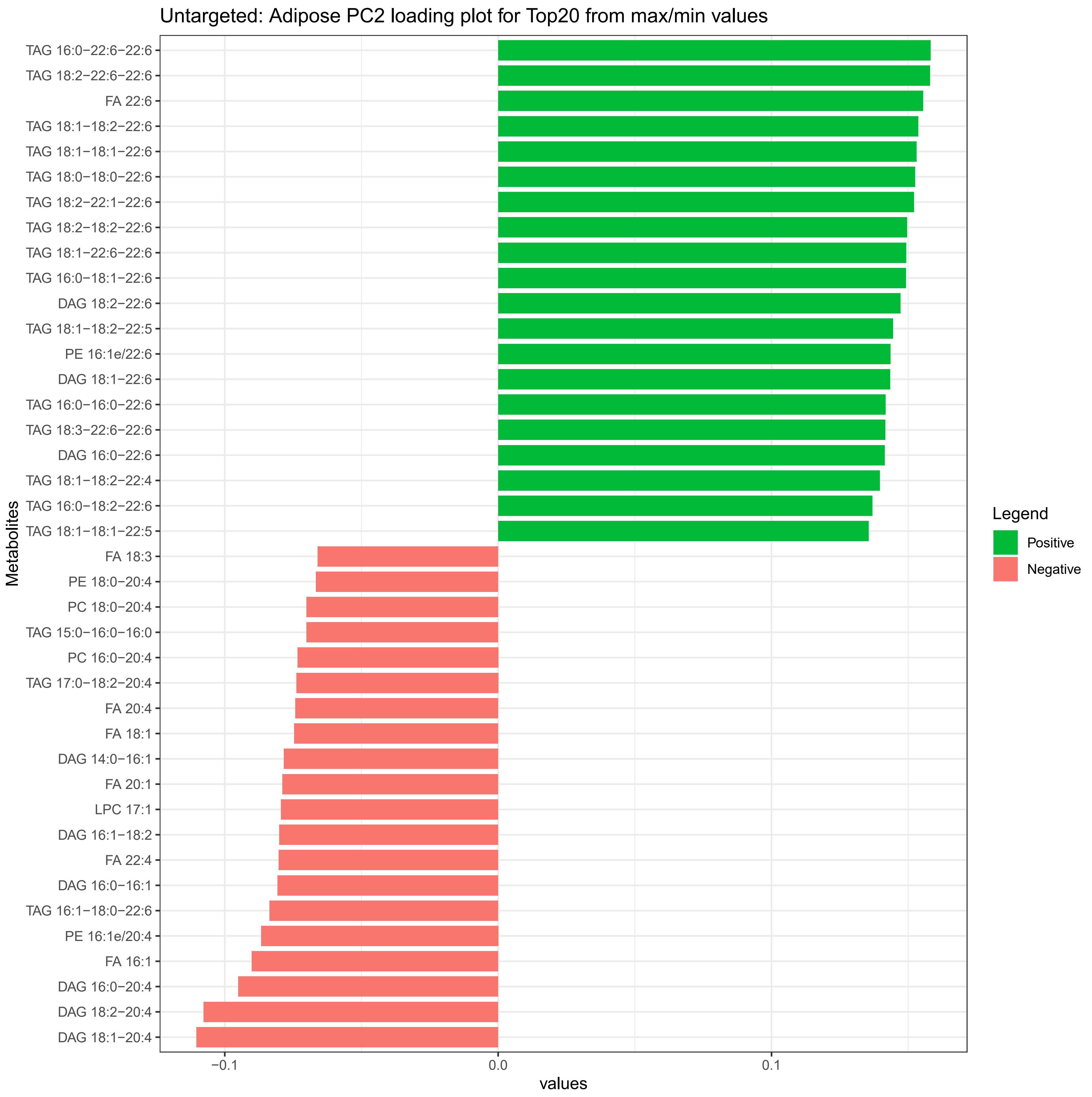

Untargeted: Adipose PC2 loading plot for Top20 from max/min values
TAG 16:0−22:6−22:6
TAG 18:2−22:6−22:6
FA 22:6
TAG 18:1−18:2−22:6
TAG 18:1−18:1−22:6
TAG 18:0−18:0−22:6
TAG 18:2−22:1−22:6
TAG 18:2−18:2−22:6
TAG 18:1−22:6−22:6
TAG 16:0−18:1−22:6
DAG 18:2−22:6
TAG 18:1−18:2−22:5
PE 16:1e/22:6
DAG 18:1−22:6
TAG 16:0−16:0−22:6
TAG 18:3−22:6−22:6
DAG 16:0−22:6
TAG 18:1−18:2−22:4
TAG 16:0−18:2−22:6
TAG 18:1−18:1−22:5
FA 18:3
PE 18:0−20:4
PC 18:0−20:4
TAG 15:0−16:0−16:0
PC 16:0−20:4
TAG 17:0−18:2−20:4
FA 20:4
FA 18:1
DAG 14:0−16:1
FA 20:1
LPC 17:1
DAG 16:1−18:2
FA 22:4
DAG 16:0−16:1
TAG 16:1−18:0−22:6
PE 16:1e/20:4
FA 16:1
DAG 16:0−20:4
DAG 18:2−20:4
DAG 18:1−20:4
Metabolites
−0.1
0.0
0.1
values
Legend
Positive
Negative

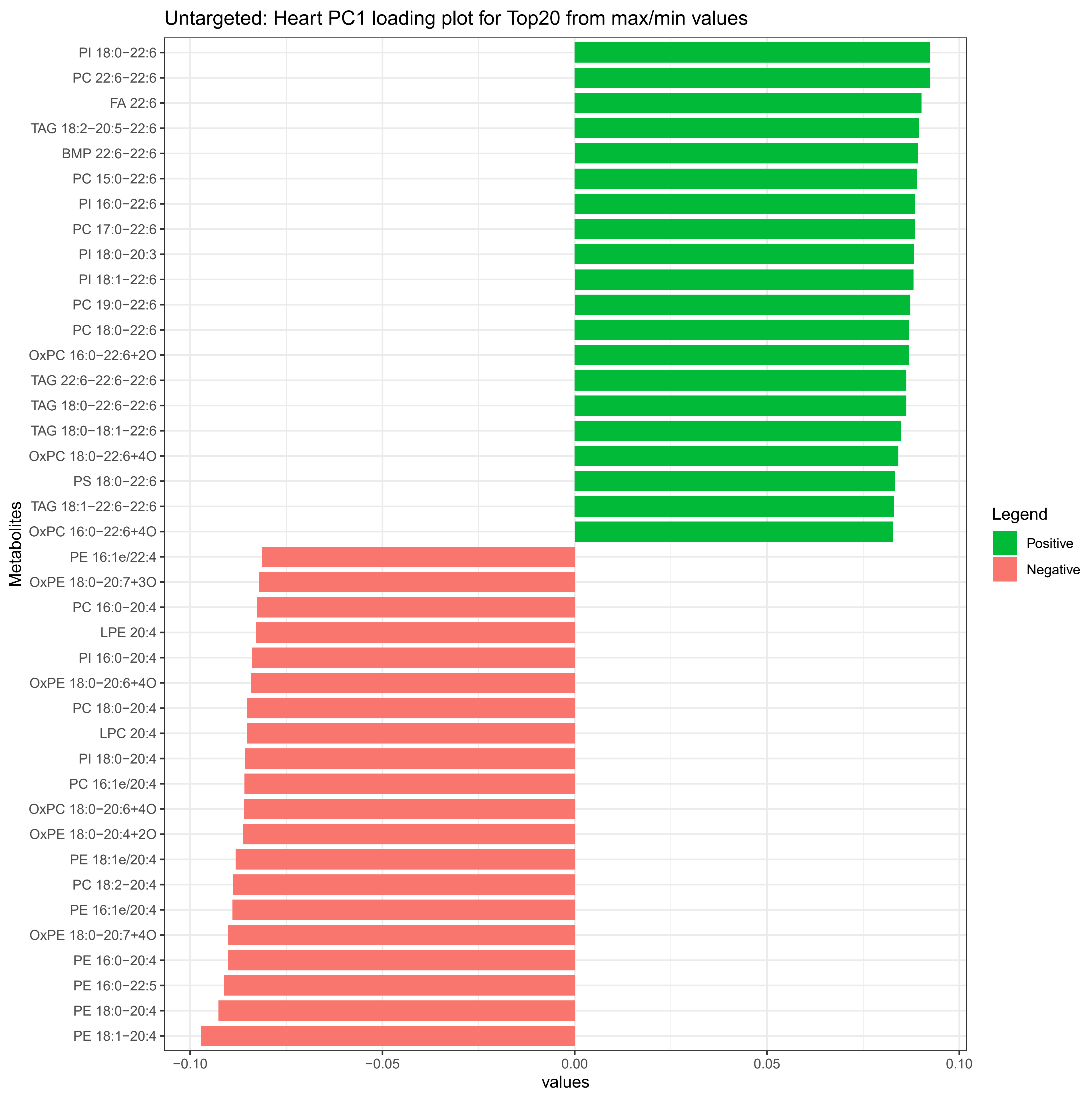
Untargeted: Heart PC1 loading plot for Top20 from max/min values
PI 18:0−22:6
PC 22:6−22:6
FA 22:6
TAG 18:2−20:5−22:6
BMP 22:6−22:6
PC 15:0−22:6
PI 16:0−22:6
PC 17:0−22:6
PI 18:0−20:3
PI 18:1−22:6
PC 19:0−22:6
PC 18:0−22:6
OxPC 16:0−22:6+2O
TAG 22:6−22:6−22:6
TAG 18:0−22:6−22:6
TAG 18:0−18:1−22:6
OxPC 18:0−22:6+4O
PS 18:0−22:6
TAG 18:1−22:6−22:6
OxPC 16:0−22:6+4O
PE 16:1e/22:4
OxPE 18:0−20:7+3O
PC 16:0−20:4
LPE 20:4
PI 16:0−20:4
OxPE 18:0−20:6+4O
PC 18:0−20:4
LPC 20:4
PI 18:0−20:4
PC 16:1e/20:4
OxPC 18:0−20:6+4O
OxPE 18:0−20:4+2O
PE 18:1e/20:4
PC 18:2−20:4
PE 16:1e/20:4
OxPE 18:0−20:7+4O
PE 16:0−20:4
PE 16:0−22:5
PE 18:0−20:4
PE 18:1−20:4
Metabolites
−0.10
−0.05
0.00
0.05
0.10
values
Legend
Positive
Negative

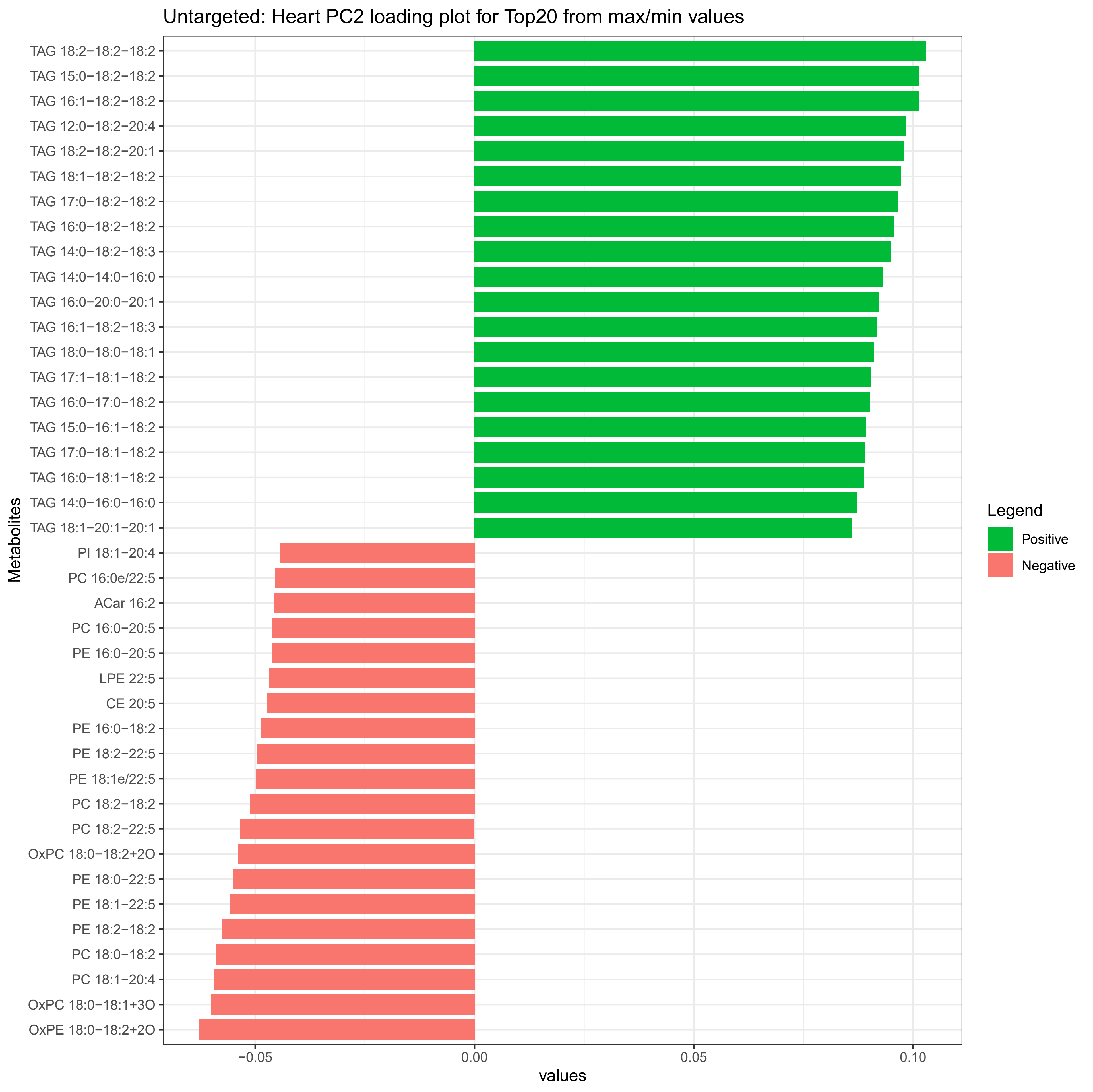
Untargeted: Heart PC2 loading plot for Top20 from max/min values
TAG 18:2−18:2−18:2
TAG 15:0−18:2−18:2
TAG 16:1−18:2−18:2
TAG 12:0−18:2−20:4
TAG 18:2−18:2−20:1
TAG 18:1−18:2−18:2
TAG 17:0−18:2−18:2
TAG 16:0−18:2−18:2
TAG 14:0−18:2−18:3
TAG 14:0−14:0−16:0
TAG 16:0−20:0−20:1
TAG 16:1−18:2−18:3
TAG 18:0−18:0−18:1
TAG 17:1−18:1−18:2
TAG 16:0−17:0−18:2
TAG 15:0−16:1−18:2
TAG 17:0−18:1−18:2
TAG 16:0−18:1−18:2
TAG 14:0−16:0−16:0
TAG 18:1−20:1−20:1
PI 18:1−20:4
PC 16:0e/22:5
ACar 16:2
PC 16:0−20:5
PE 16:0−20:5
LPE 22:5
CE 20:5
PE 16:0−18:2
PE 18:2−22:5
PE 18:1e/22:5
PC 18:2−18:2
PC 18:2−22:5
OxPC 18:0−18:2+2O
PE 18:0−22:5
PE 18:1−22:5
PE 18:2−18:2
PC 18:0−18:2
PC 18:1−20:4
OxPC 18:0−18:1+3O
OxPE 18:0−18:2+2O
Metabolites
−0.05
0.00
0.05
0.10
values
Legend
Positive
Negative

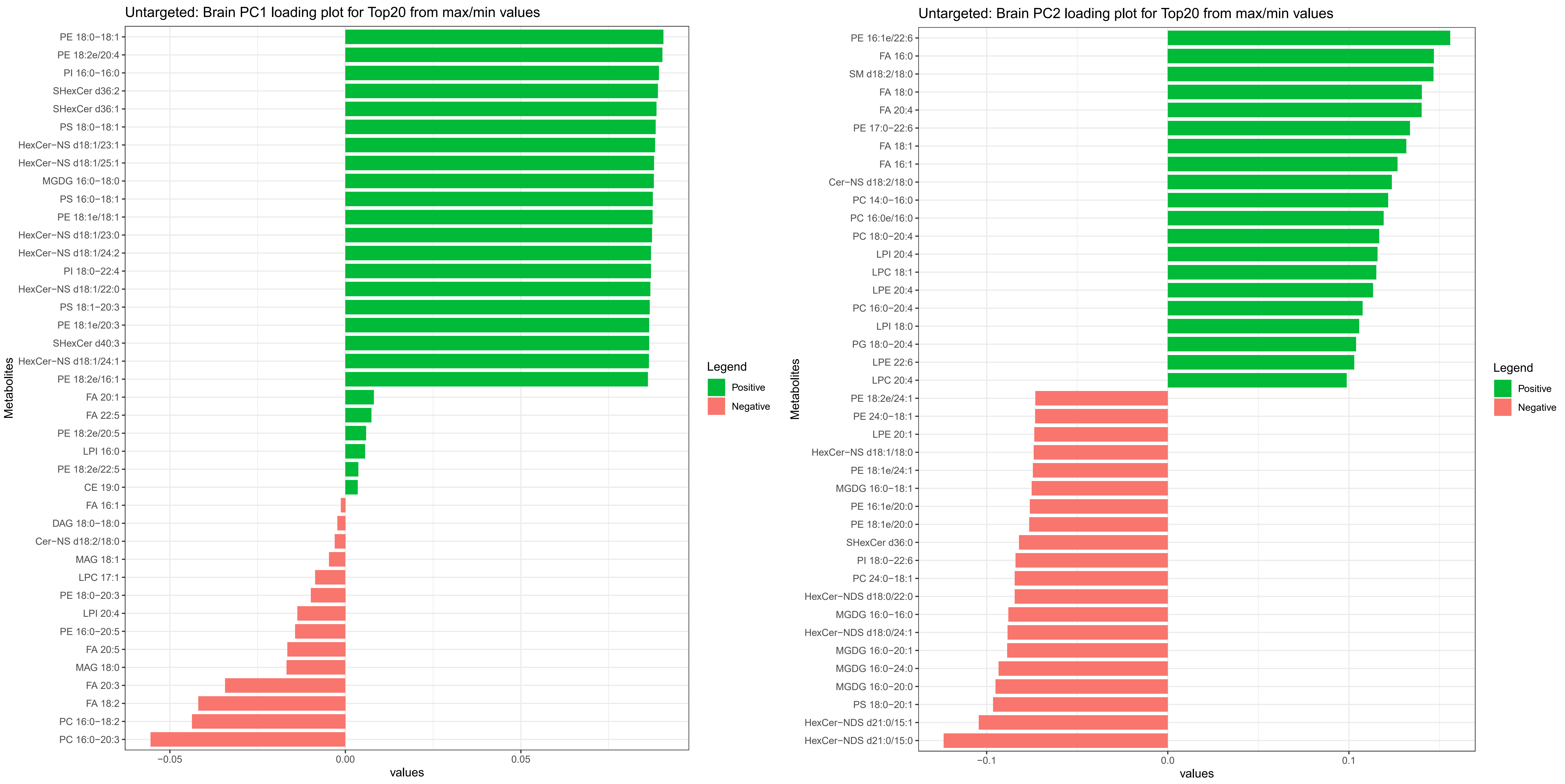
Untargeted: Brain PC1 loading plot for Top20 from max/min values
PE 18:0−18:1
PE 18:2e/20:4
PI 16:0−16:0
SHexCer d36:2
SHexCer d36:1
PS 18:0−18:1
HexCer−NS d18:1/23:1
HexCer−NS d18:1/25:1
MGDG 16:0−18:0
PS 16:0−18:1
PE 18:1e/18:1
HexCer−NS d18:1/23:0
HexCer−NS d18:1/24:2
PI 18:0−22:4
HexCer−NS d18:1/22:0
PS 18:1−20:3
PE 18:1e/20:3
SHexCer d40:3
HexCer−NS d18:1/24:1
PE 18:2e/16:1
FA 20:1
FA 22:5
PE 18:2e/20:5
LPI 16:0
PE 18:2e/22:5
CE 19:0
FA 16:1
DAG 18:0−18:0
Cer−NS d18:2/18:0
MAG 18:1
LPC 17:1
PE 18:0−20:3
LPI 20:4
PE 16:0−20:5
FA 20:5
MAG 18:0
FA 20:3
FA 18:2
PC 16:0−18:2
PC 16:0−20:3
Metabolites
−0.05
0.00
0.05
values
Legend
Positive
Negative
Untargeted: Brain PC2 loading plot for Top20 from max/min values
PE 16:1e/22:6
FA 16:0
SM d18:2/18:0
FA 18:0
FA 20:4
PE 17:0−22:6
FA 18:1
FA 16:1
Cer−NS d18:2/18:0
PC 14:0−16:0
PC 16:0e/16:0
PC 18:0−20:4
LPI 20:4
LPC 18:1
LPE 20:4
PC 16:0−20:4
LPI 18:0
PG 18:0−20:4
LPE 22:6
LPC 20:4
PE 18:2e/24:1
PE 24:0−18:1
LPE 20:1
HexCer−NS d18:1/18:0
PE 18:1e/24:1
MGDG 16:0−18:1
PE 16:1e/20:0
PE 18:1e/20:0
SHexCer d36:0
PI 18:0−22:6
PC 24:0−18:1
HexCer−NDS d18:0/22:0
MGDG 16:0−16:0
HexCer−NDS d18:0/24:1
MGDG 16:0−20:1
MGDG 16:0−24:0
MGDG 16:0−20:0
PS 18:0−20:1
HexCer−NDS d21:0/15:1
HexCer−NDS d21:0/15:0
Metabolites
−0.1
0.0
0.1
values
Legend
Positive
Negative

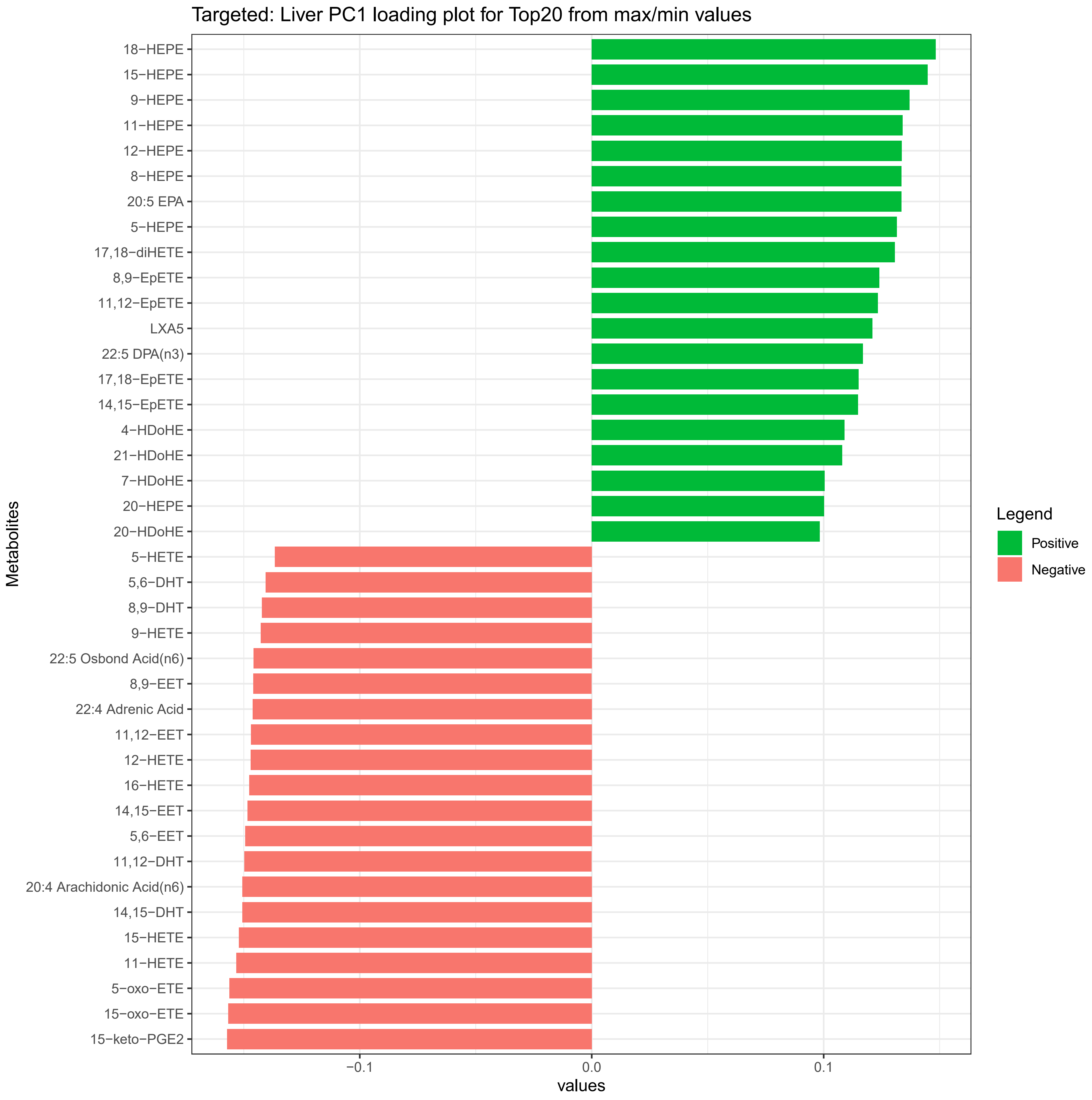

Targeted: Liver PC1 loading plot for Top20 from max/min values
18−HEPE
15−HEPE
9−HEPE
11−HEPE
12−HEPE
8−HEPE
20:5 EPA
5−HEPE
17,18−diHETE
8,9−EpETE
11,12−EpETE
LXA5
22:5 DPA(n3)
17,18−EpETE
14,15−EpETE
4−HDoHE
21−HDoHE
7−HDoHE
20−HEPE
20−HDoHE
5−HETE
5,6−DHT
8,9−DHT
9−HETE
22:5 Osbond Acid(n6)
8,9−EET
22:4 Adrenic Acid
11,12−EET
12−HETE
16−HETE
14,15−EET
5,6−EET
11,12−DHT
20:4 Arachidonic Acid(n6)
14,15−DHT
15−HETE
11−HETE
5−oxo−ETE
15−oxo−ETE
15−keto−PGE2
Metabolites
−0.1
0.0
0.1
values
Legend
Positive
Negative

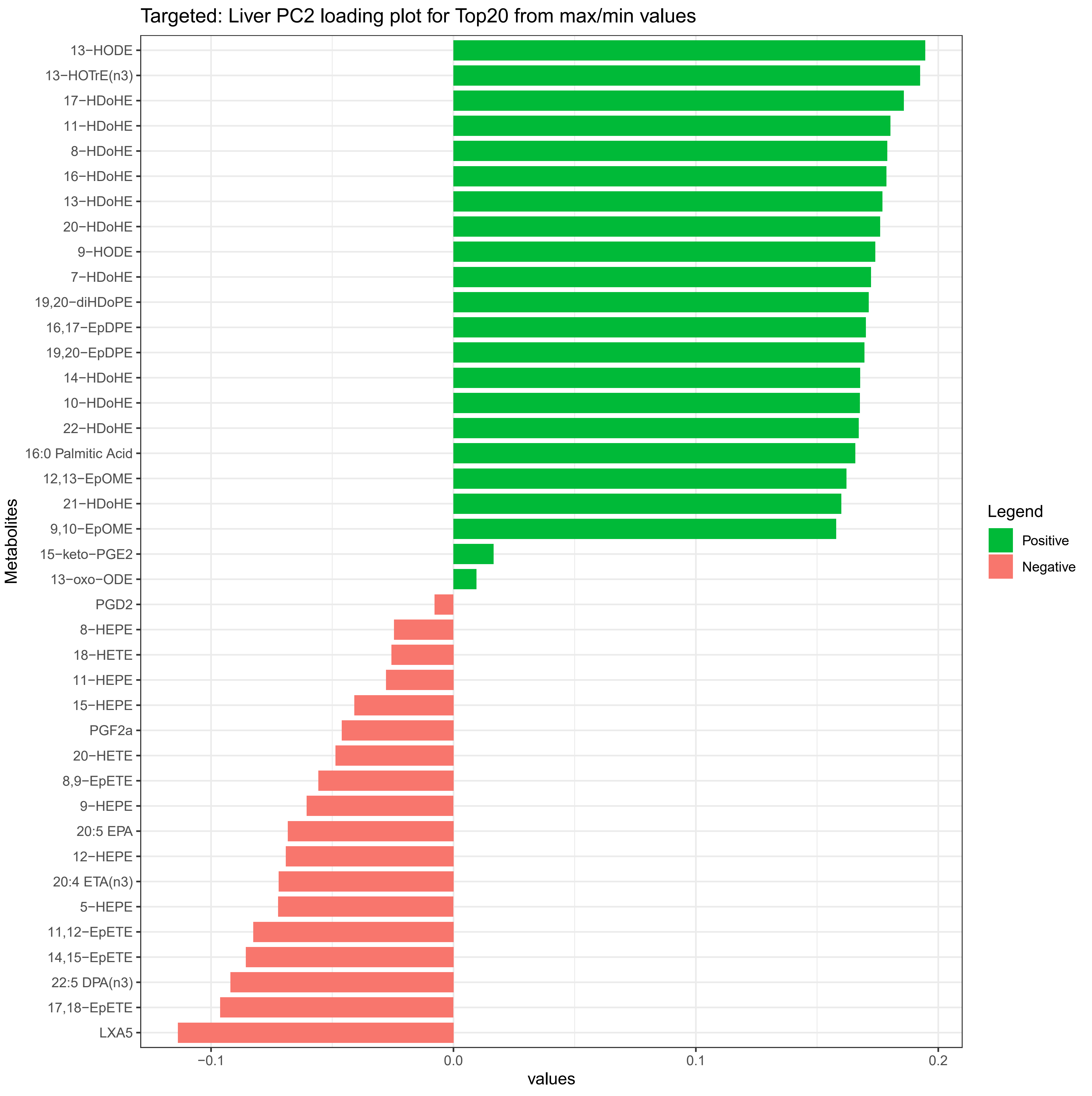

Targeted: Liver PC2 loading plot for Top20 from max/min values
13−HODE
13−HOTrE(n3)
17−HDoHE
11−HDoHE
8−HDoHE
16−HDoHE
13−HDoHE
20−HDoHE
9−HODE
7−HDoHE
19,20−diHDoPE
16,17−EpDPE
19,20−EpDPE
14−HDoHE
10−HDoHE
22−HDoHE
16:0 Palmitic Acid
12,13−EpOME
21−HDoHE
9,10−EpOME
15−keto−PGE2
13−oxo−ODE
PGD2
8−HEPE
18−HETE
11−HEPE
15−HEPE
PGF2a
20−HETE
8,9−EpETE
9−HEPE
20:5 EPA
12−HEPE
20:4 ETA(n3)
5−HEPE
11,12−EpETE
14,15−EpETE
22:5 DPA(n3)
17,18−EpETE
LXA5
Metabolites
−0.1
0.0
0.1
0.2
values
Legend
Positive
Negative

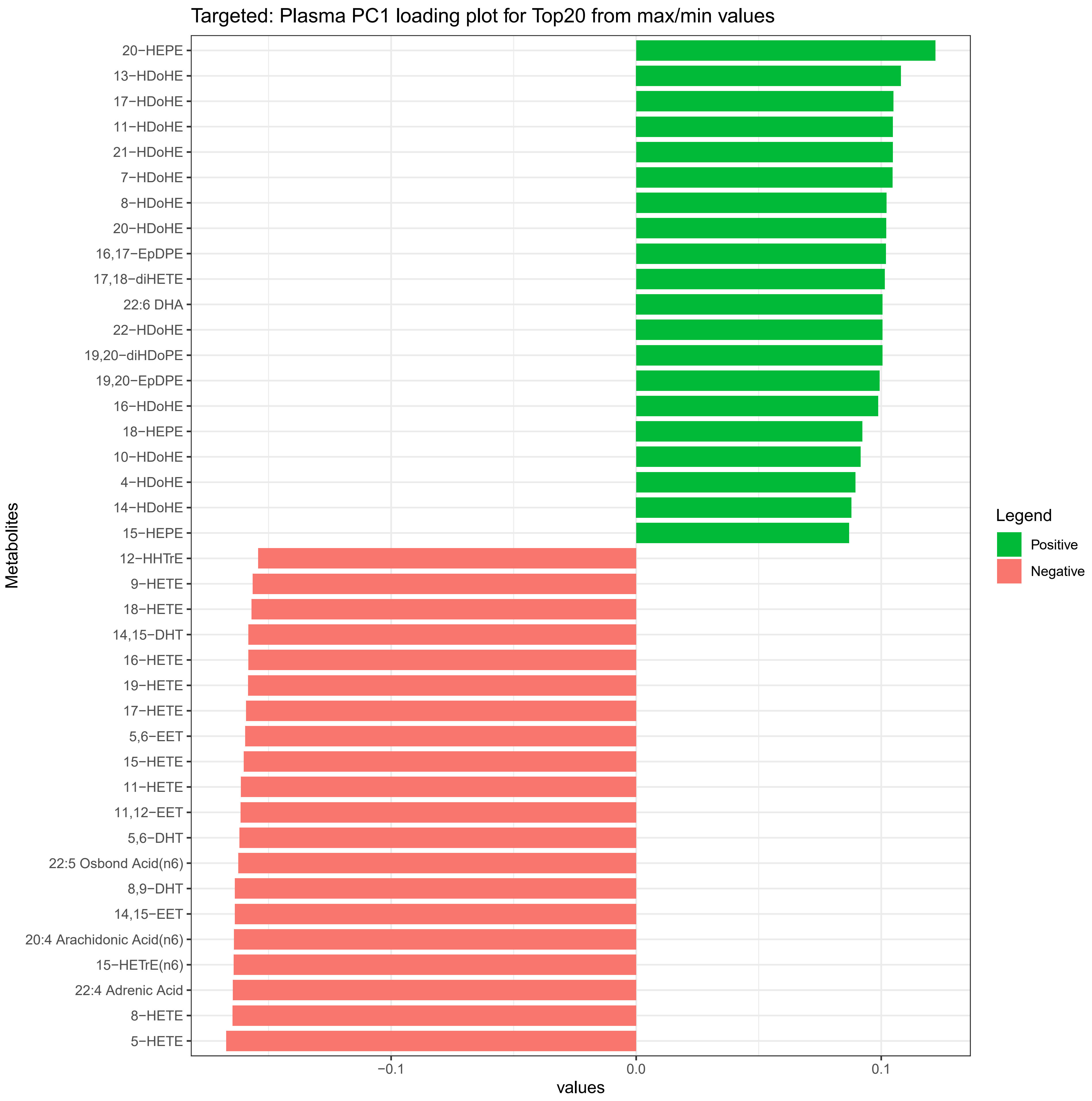
Targeted: Plasma PC1 loading plot for Top20 from max/min values
20−HEPE
13−HDoHE
17−HDoHE
11−HDoHE
21−HDoHE
7−HDoHE
8−HDoHE
20−HDoHE
16,17−EpDPE
17,18−diHETE
22:6 DHA
22−HDoHE
19,20−diHDoPE
19,20−EpDPE
16−HDoHE
18−HEPE
10−HDoHE
4−HDoHE
14−HDoHE
15−HEPE
12−HHTrE
9−HETE
18−HETE
14,15−DHT
16−HETE
19−HETE
17−HETE
5,6−EET
15−HETE
11−HETE
11,12−EET
5,6−DHT
22:5 Osbond Acid(n6)
8,9−DHT
14,15−EET
20:4 Arachidonic Acid(n6)
15−HETrE(n6)
22:4 Adrenic Acid
8−HETE
5−HETE
Metabolites
−0.1
0.0
0.1
values
Legend
Positive
Negative

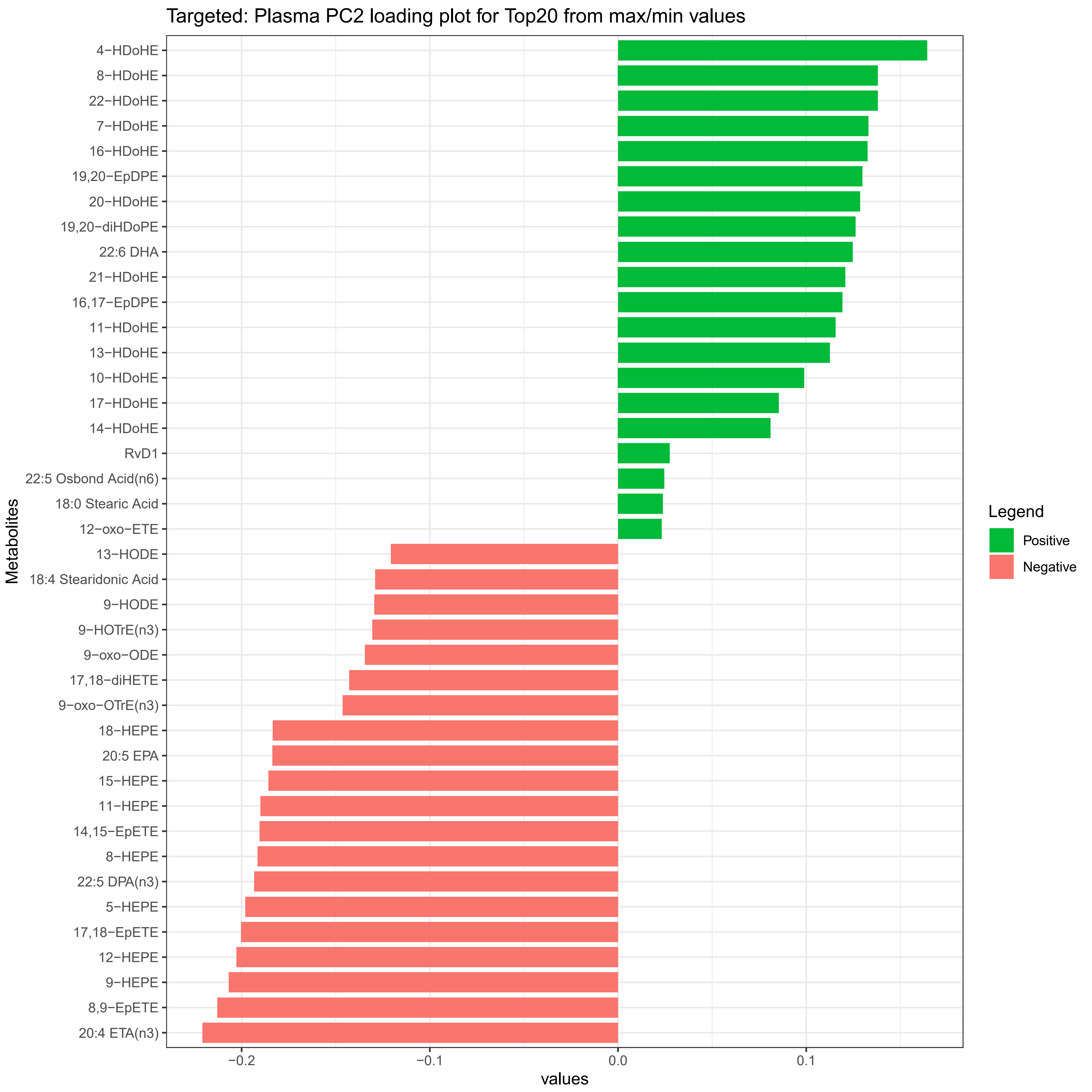
Targeted: Plasma PC2 loading plot for Top20 from max/min values
4−HDoHE
8−HDoHE
22−HDoHE
7−HDoHE
16−HDoHE
19,20−EpDPE
20−HDoHE
19,20−diHDoPE
22:6 DHA
21−HDoHE
16,17−EpDPE
11−HDoHE
13−HDoHE
10−HDoHE
17−HDoHE
14−HDoHE
RvD1
22:5 Osbond Acid(n6)
18:0 Stearic Acid
12−oxo−ETE
13−HODE
18:4 Stearidonic Acid
9−HODE
9−HOTrE(n3)
9−oxo−ODE
17,18−diHETE
9−oxo−OTrE(n3)
18−HEPE
20:5 EPA
15−HEPE
11−HEPE
14,15−EpETE
8−HEPE
22:5 DPA(n3)
5−HEPE
17,18−EpETE
12−HEPE
9−HEPE
8,9−EpETE
20:4 ETA(n3)
Metabolites
−0.2
−0.1
0.0
0.1
values
Legend
Positive
Negative

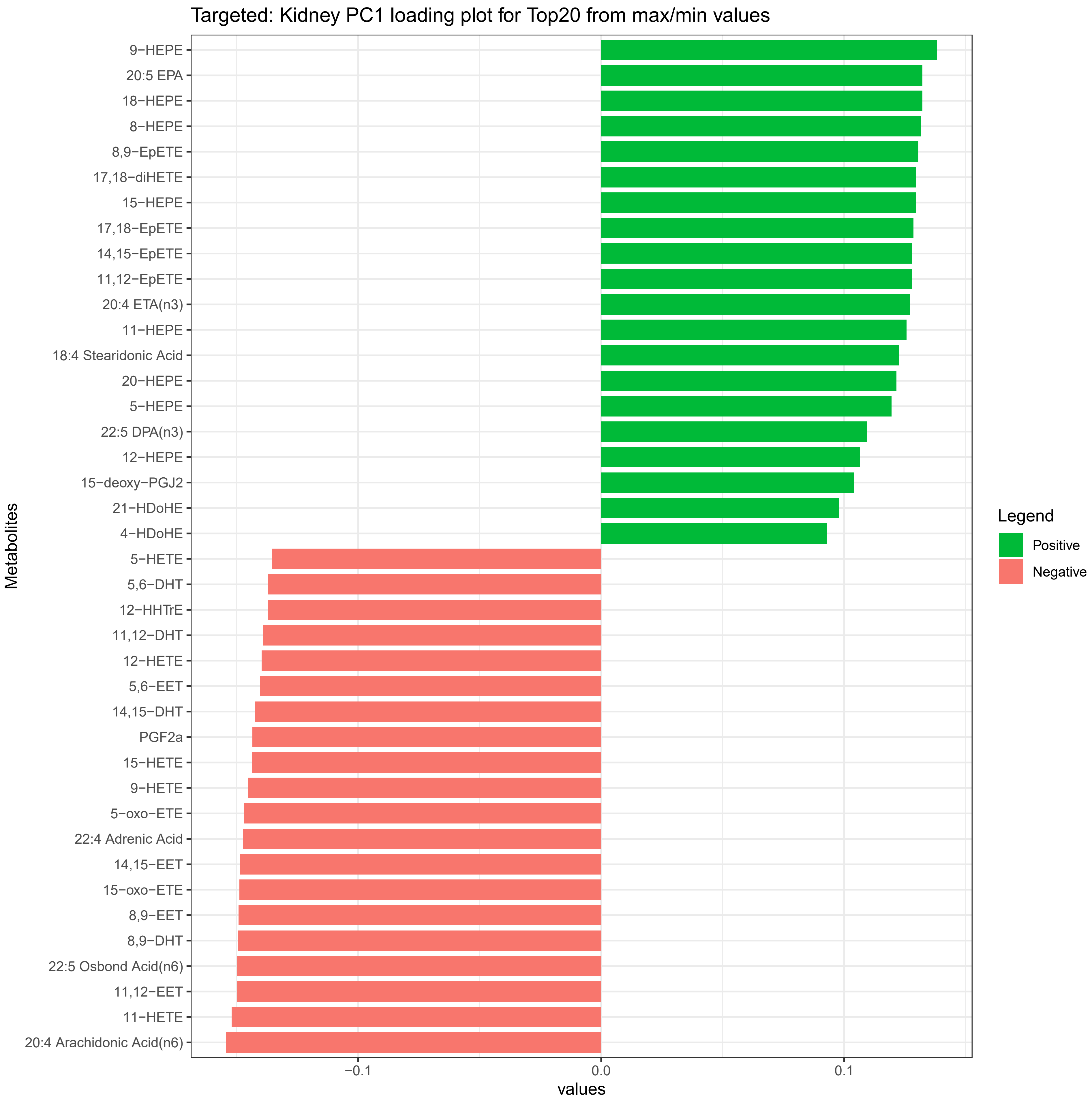

Targeted: Kidney PC1 loading plot for Top20 from max/min values
9−HEPE
20:5 EPA
18−HEPE
8−HEPE
8,9−EpETE
17,18−diHETE
15−HEPE
17,18−EpETE
14,15−EpETE
11,12−EpETE
20:4 ETA(n3)
11−HEPE
18:4 Stearidonic Acid
20−HEPE
5−HEPE
22:5 DPA(n3)
12−HEPE
15−deoxy−PGJ2
21−HDoHE
4−HDoHE
5−HETE
5,6−DHT
12−HHTrE
11,12−DHT
12−HETE
5,6−EET
14,15−DHT
PGF2a
15−HETE
9−HETE
5−oxo−ETE
22:4 Adrenic Acid
14,15−EET
15−oxo−ETE
8,9−EET
8,9−DHT
22:5 Osbond Acid(n6)
11,12−EET
11−HETE
20:4 Arachidonic Acid(n6)
Metabolites
−0.1
0.0
0.1
values
Legend
Positive
Negative

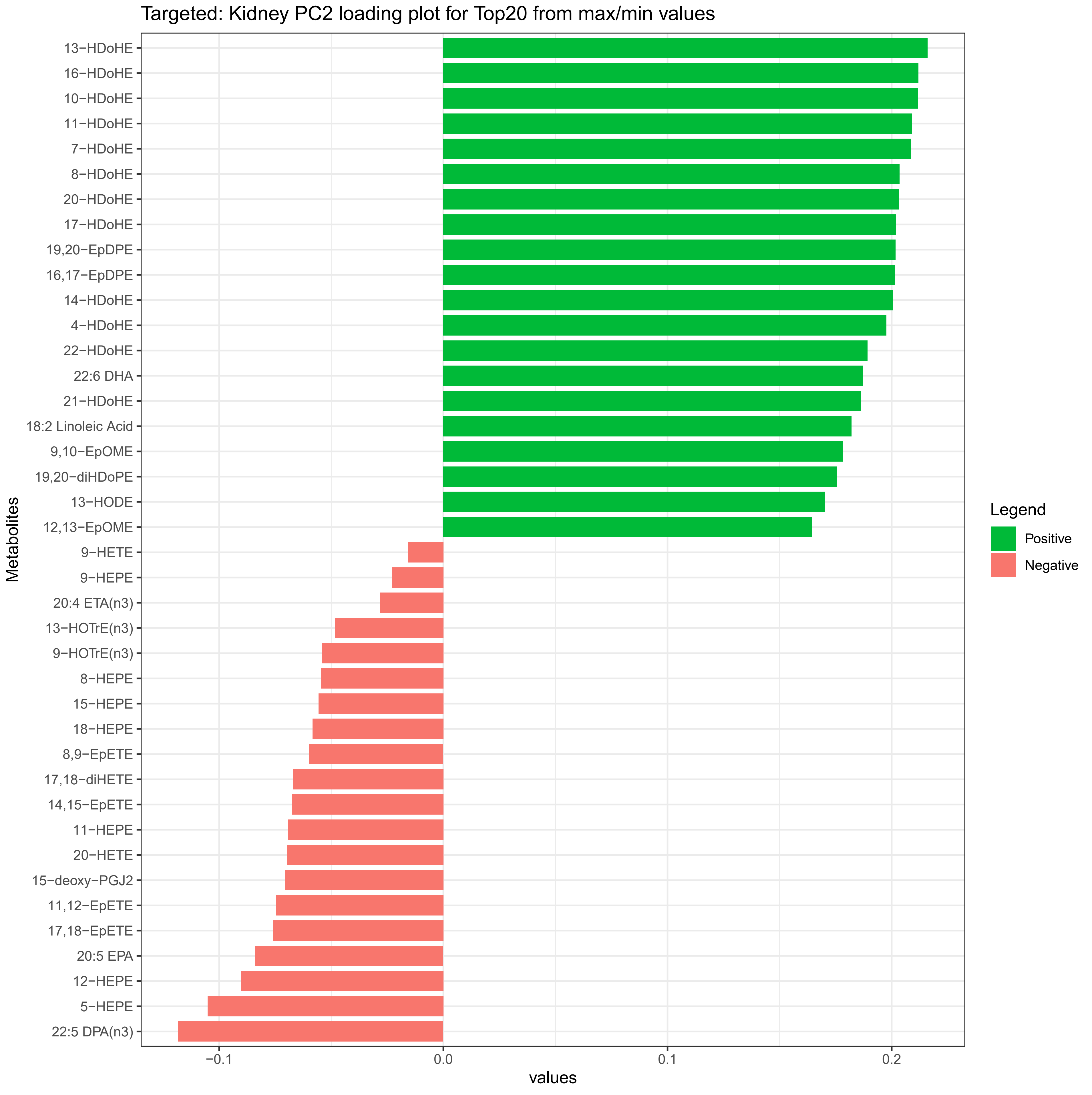

Targeted: Kidney PC2 loading plot for Top20 from max/min values
13−HDoHE
16−HDoHE
10−HDoHE
11−HDoHE
7−HDoHE
8−HDoHE
20−HDoHE
17−HDoHE
19,20−EpDPE
16,17−EpDPE
14−HDoHE
4−HDoHE
22−HDoHE
22:6 DHA
21−HDoHE
18:2 Linoleic Acid
9,10−EpOME
19,20−diHDoPE
13−HODE
12,13−EpOME
9−HETE
9−HEPE
20:4 ETA(n3)
13−HOTrE(n3)
9−HOTrE(n3)
8−HEPE
15−HEPE
18−HEPE
8,9−EpETE
17,18−diHETE
14,15−EpETE
11−HEPE
20−HETE
15−deoxy−PGJ2
11,12−EpETE
17,18−EpETE
20:5 EPA
12−HEPE
5−HEPE
22:5 DPA(n3)
Metabolites
−0.1
0.0
0.1
0.2
values
Legend
Positive
Negative

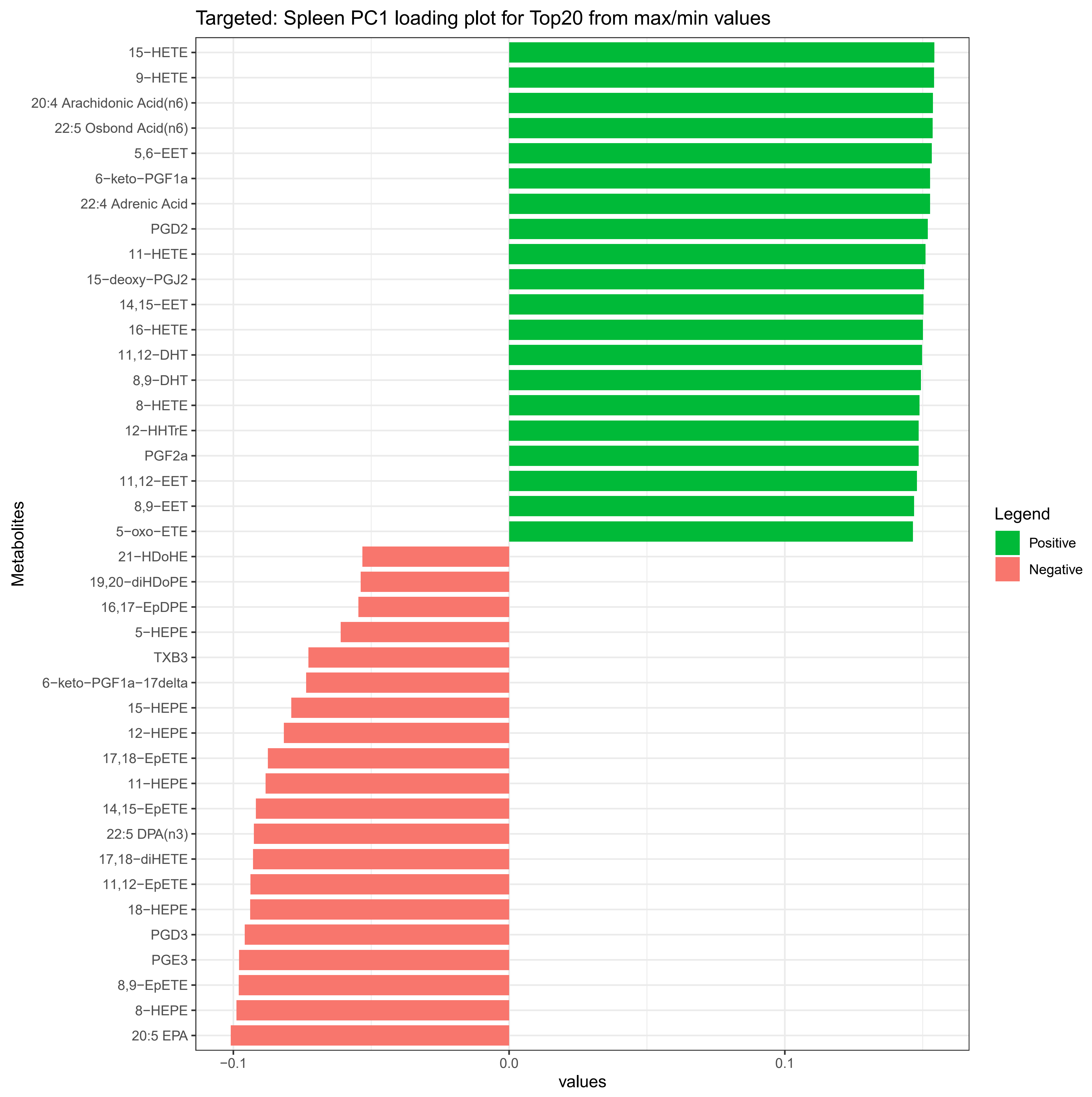

Targeted: Spleen PC1 loading plot for Top20 from max/min values
15−HETE
9−HETE
20:4 Arachidonic Acid(n6)
22:5 Osbond Acid(n6)
5,6−EET
6−keto−PGF1a
22:4 Adrenic Acid
PGD2
11−HETE
15−deoxy−PGJ2
14,15−EET
16−HETE
11,12−DHT
8,9−DHT
8−HETE
12−HHTrE
PGF2a
11,12−EET
8,9−EET
5−oxo−ETE
21−HDoHE
19,20−diHDoPE
16,17−EpDPE
5−HEPE
TXB3
6−keto−PGF1a−17delta
15−HEPE
12−HEPE
17,18−EpETE
11−HEPE
14,15−EpETE
22:5 DPA(n3)
17,18−diHETE
11,12−EpETE
18−HEPE
PGD3
PGE3
8,9−EpETE
8−HEPE
20:5 EPA
Metabolites
−0.1
0.0
0.1
values
Legend
Positive
Negative

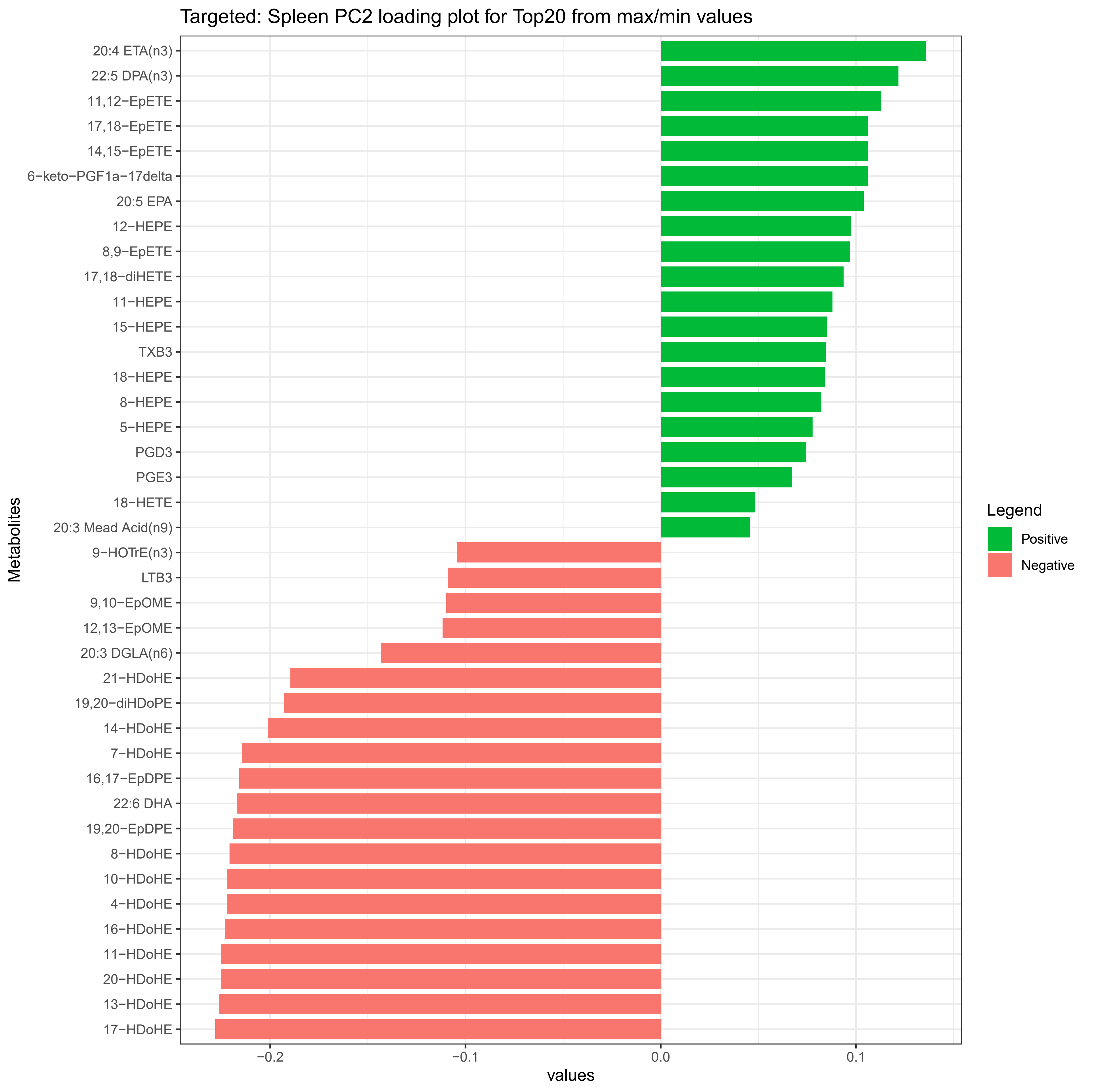

Targeted: Spleen PC2 loading plot for Top20 from max/min values
20:4 ETA(n3)
22:5 DPA(n3)
11,12−EpETE
17,18−EpETE
14,15−EpETE
6−keto−PGF1a−17delta
20:5 EPA
12−HEPE
8,9−EpETE
17,18−diHETE
11−HEPE
15−HEPE
TXB3
18−HEPE
8−HEPE
5−HEPE
PGD3
PGE3
18−HETE
20:3 Mead Acid(n9)
9−HOTrE(n3)
LTB3
9,10−EpOME
12,13−EpOME
20:3 DGLA(n6)
21−HDoHE
19,20−diHDoPE
14−HDoHE
7−HDoHE
16,17−EpDPE
22:6 DHA
19,20−EpDPE
8−HDoHE
10−HDoHE
4−HDoHE
16−HDoHE
11−HDoHE
20−HDoHE
13−HDoHE
17−HDoHE
Metabolites
−0.2
−0.1
0.0
0.1
values
Legend
Positive
Negative

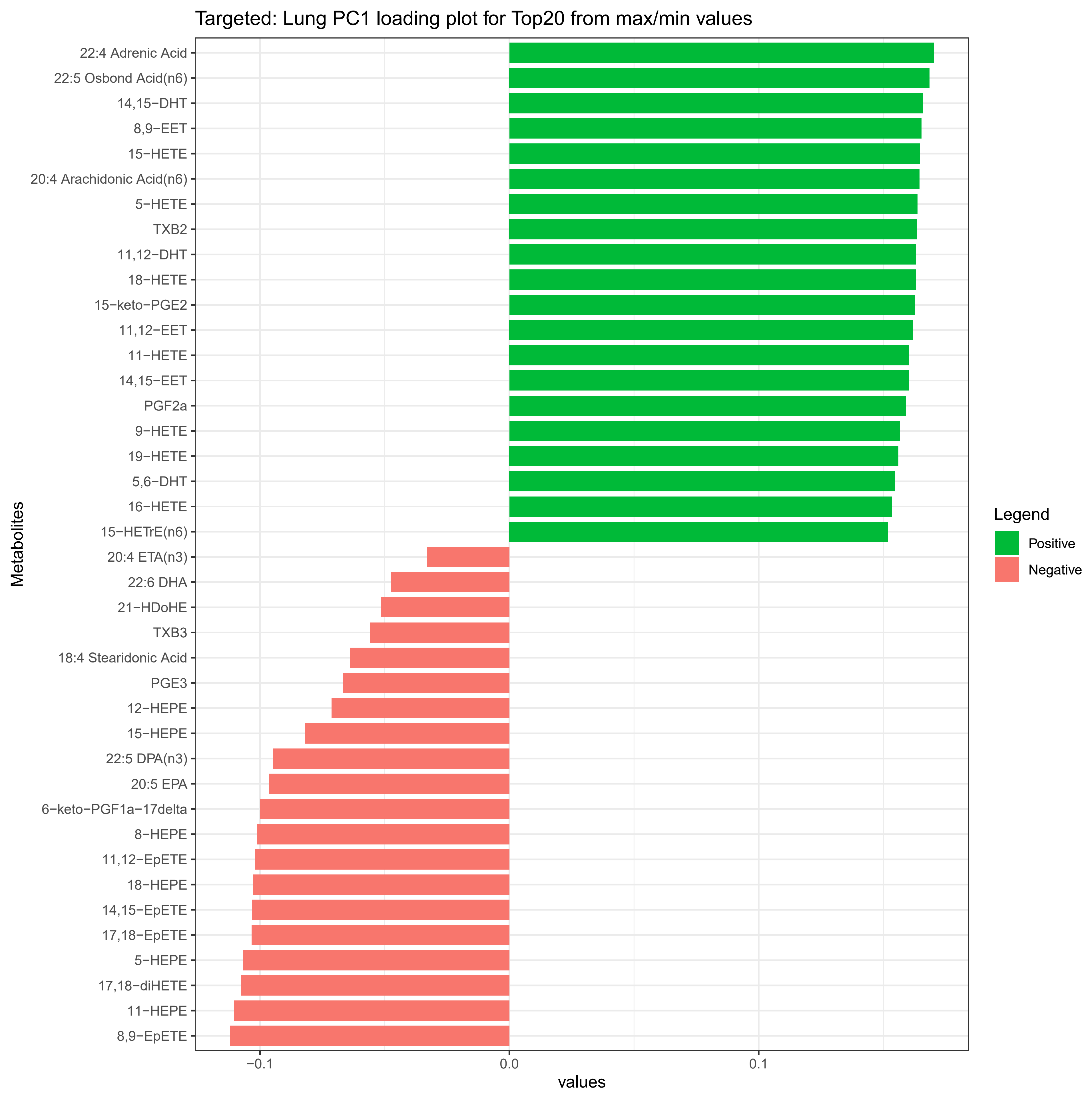
Targeted: Lung PC1 loading plot for Top20 from max/min values
Metabolites
22:4 Adrenic Acid
22:5 Osbond Acid(n6)
14,15−DHT
8,9−EET
15−HETE
20:4 Arachidonic Acid(n6)
5−HETE
TXB2
11,12−DHT
18−HETE
15−keto−PGE2
11,12−EET
11−HETE
14,15−EET
PGF2a
9−HETE
19−HETE
5,6−DHT
16−HETE
15−HETrE(n6)
20:4 ETA(n3)
22:6 DHA
21−HDoHE
TXB3
18:4 Stearidonic Acid
PGE3
12−HEPE
15−HEPE
22:5 DPA(n3)
20:5 EPA
6−keto−PGF1a−17delta
8−HEPE
11,12−EpETE
18−HEPE
14,15−EpETE
17,18−EpETE
5−HEPE
17,18−diHETE
11−HEPE
8,9−EpETE
−0.1
0.0
0.1
values
Legend
Positive
Negative

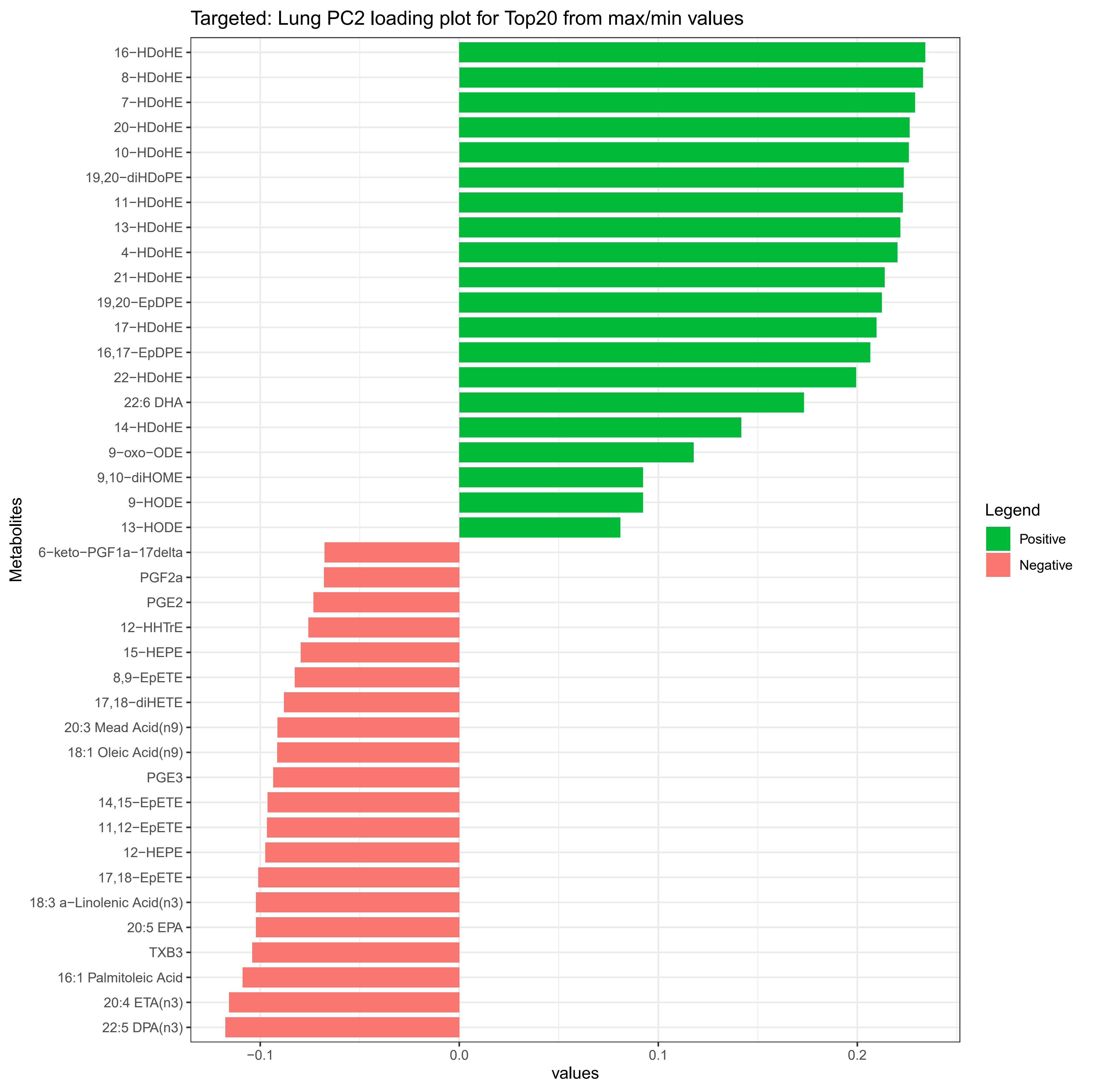
Targeted: Lung PC2 loading plot for Top20 from max/min values
Metabolites
16−HDoHE
8−HDoHE
7−HDoHE
20−HDoHE
10−HDoHE
19,20−diHDoPE
11−HDoHE
13−HDoHE
4−HDoHE
21−HDoHE
19,20−EpDPE
17−HDoHE
16,17−EpDPE
22−HDoHE
22:6 DHA
14−HDoHE
9−oxo−ODE
9,10−diHOME
9−HODE
13−HODE
6−keto−PGF1a−17delta
PGF2a
PGE2
12−HHTrE
15−HEPE
8,9−EpETE
17,18−diHETE
20:3 Mead Acid(n9)
18:1 Oleic Acid(n9)
PGE3
14,15−EpETE
11,12−EpETE
12−HEPE
17,18−EpETE
18:3 a−Linolenic Acid(n3)
20:5 EPA
TXB3
16:1 Palmitoleic Acid
20:4 ETA(n3)
22:5 DPA(n3)
−0.1
0.0
0.1
0.2
values
Legend
Positive
Negative

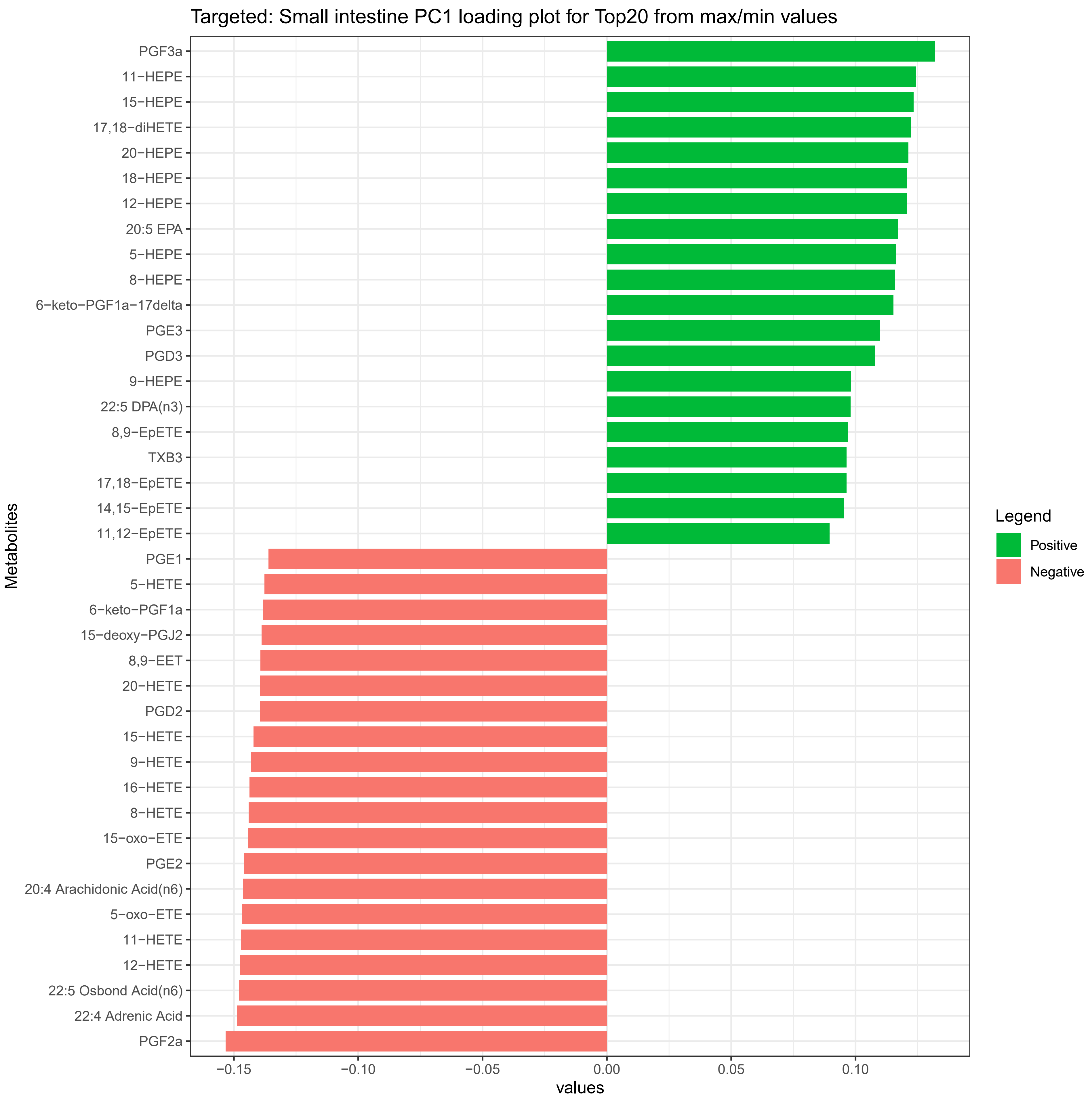
Targeted: Small intestine PC1 loading plot for Top20 from max/min values
Metabolites
PGF3a
11−HEPE
15−HEPE
17,18−diHETE
20−HEPE
18−HEPE
12−HEPE
20:5 EPA
5−HEPE
8−HEPE
6−keto−PGF1a−17delta
PGE3
PGD3
9−HEPE
22:5 DPA(n3)
8,9−EpETE
TXB3
17,18−EpETE
14,15−EpETE
11,12−EpETE
PGE1
5−HETE
6−keto−PGF1a
15−deoxy−PGJ2
8,9−EET
20−HETE
PGD2
15−HETE
9−HETE
16−HETE
8−HETE
15−oxo−ETE
PGE2
20:4 Arachidonic Acid(n6)
5−oxo−ETE
11−HETE
12−HETE
22:5 Osbond Acid(n6)
22:4 Adrenic Acid
PGF2a
−0.15
−0.10
−0.05
0.00
0.05
0.10
values
Legend
Positive
Negative

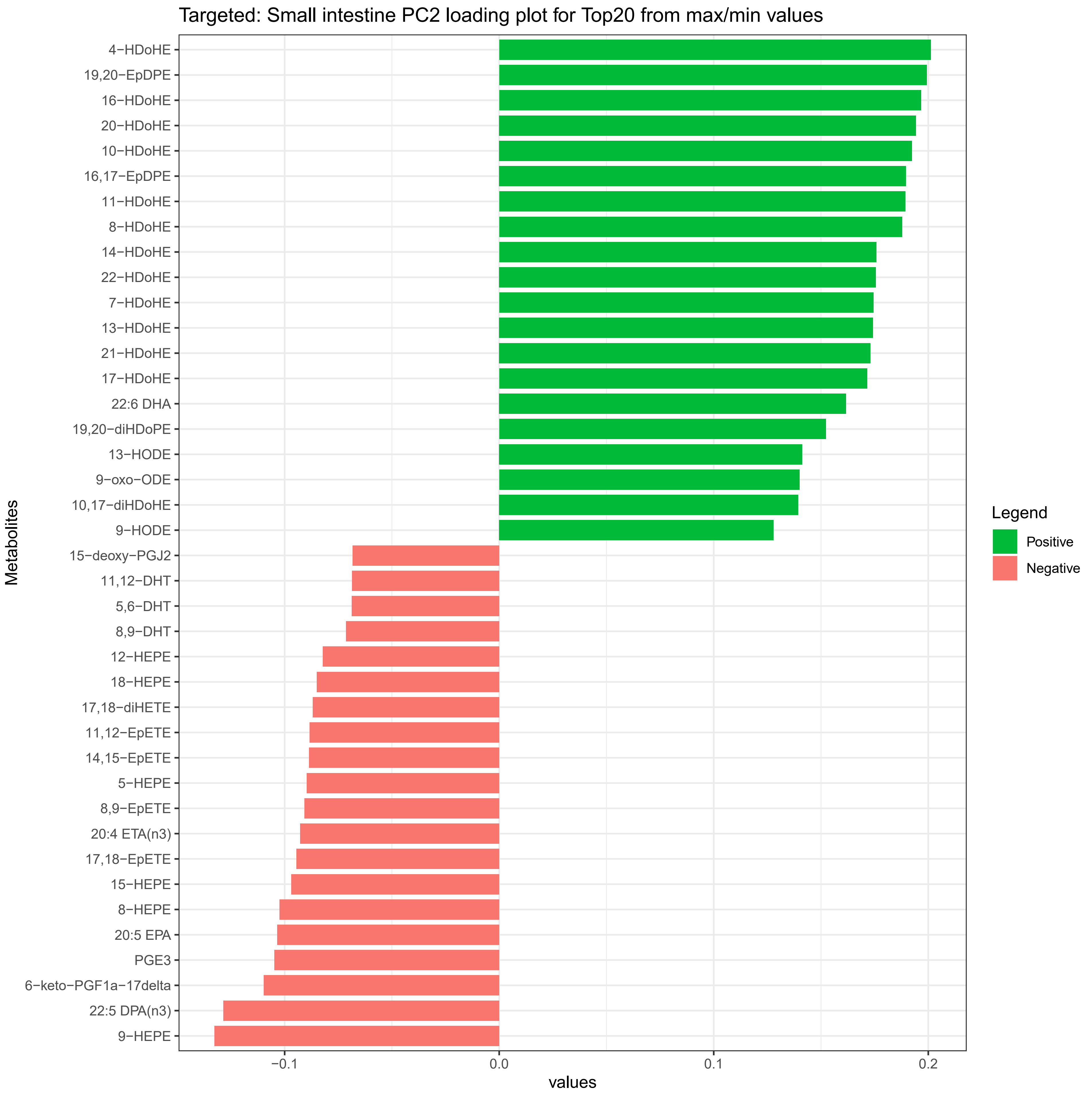
Targeted: Small intestine PC2 loading plot for Top20 from max/min values
Metabolites
4−HDoHE
19,20−EpDPE
16−HDoHE
20−HDoHE
10−HDoHE
16,17−EpDPE
11−HDoHE
8−HDoHE
14−HDoHE
22−HDoHE
7−HDoHE
13−HDoHE
21−HDoHE
17−HDoHE
22:6 DHA
19,20−diHDoPE
13−HODE
9−oxo−ODE
10,17−diHDoHE
9−HODE
15−deoxy−PGJ2
11,12−DHT
5,6−DHT
8,9−DHT
12−HEPE
18−HEPE
17,18−diHETE
11,12−EpETE
14,15−EpETE
5−HEPE
8,9−EpETE
20:4 ETA(n3)
17,18−EpETE
15−HEPE
8−HEPE
20:5 EPA
PGE3
6−keto−PGF1a−17delta
22:5 DPA(n3)
9−HEPE
−0.1
0.0
0.1
0.2
values
Legend
Positive
Negative

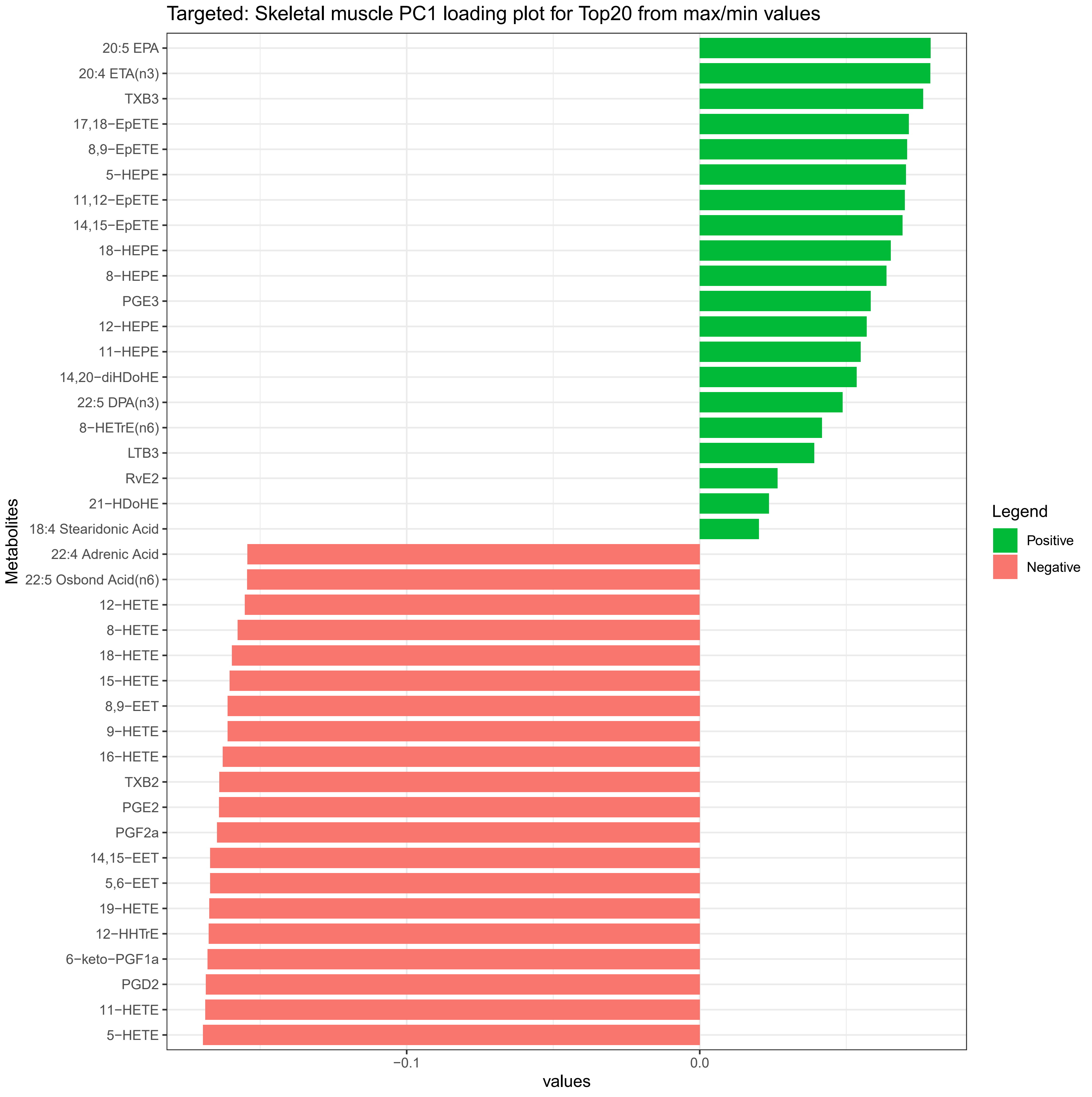

Targeted: Skeletal muscle PC1 loading plot for Top20 from max/min values
20:5 EPA
20:4 ETA(n3)
TXB3
17,18−EpETE
8,9−EpETE
5−HEPE
11,12−EpETE
14,15−EpETE
18−HEPE
8−HEPE
PGE3
12−HEPE
11−HEPE
14,20−diHDoHE
22:5 DPA(n3)
8−HETrE(n6)
LTB3
RvE2
21−HDoHE
18:4 Stearidonic Acid
22:4 Adrenic Acid
22:5 Osbond Acid(n6)
12−HETE
8−HETE
18−HETE
15−HETE
8,9−EET
9−HETE
16−HETE
TXB2
PGE2
PGF2a
14,15−EET
5,6−EET
19−HETE
12−HHTrE
6−keto−PGF1a
PGD2
11−HETE
5−HETE
Metabolites
−0.1
0.0
values
Legend
Positive
Negative

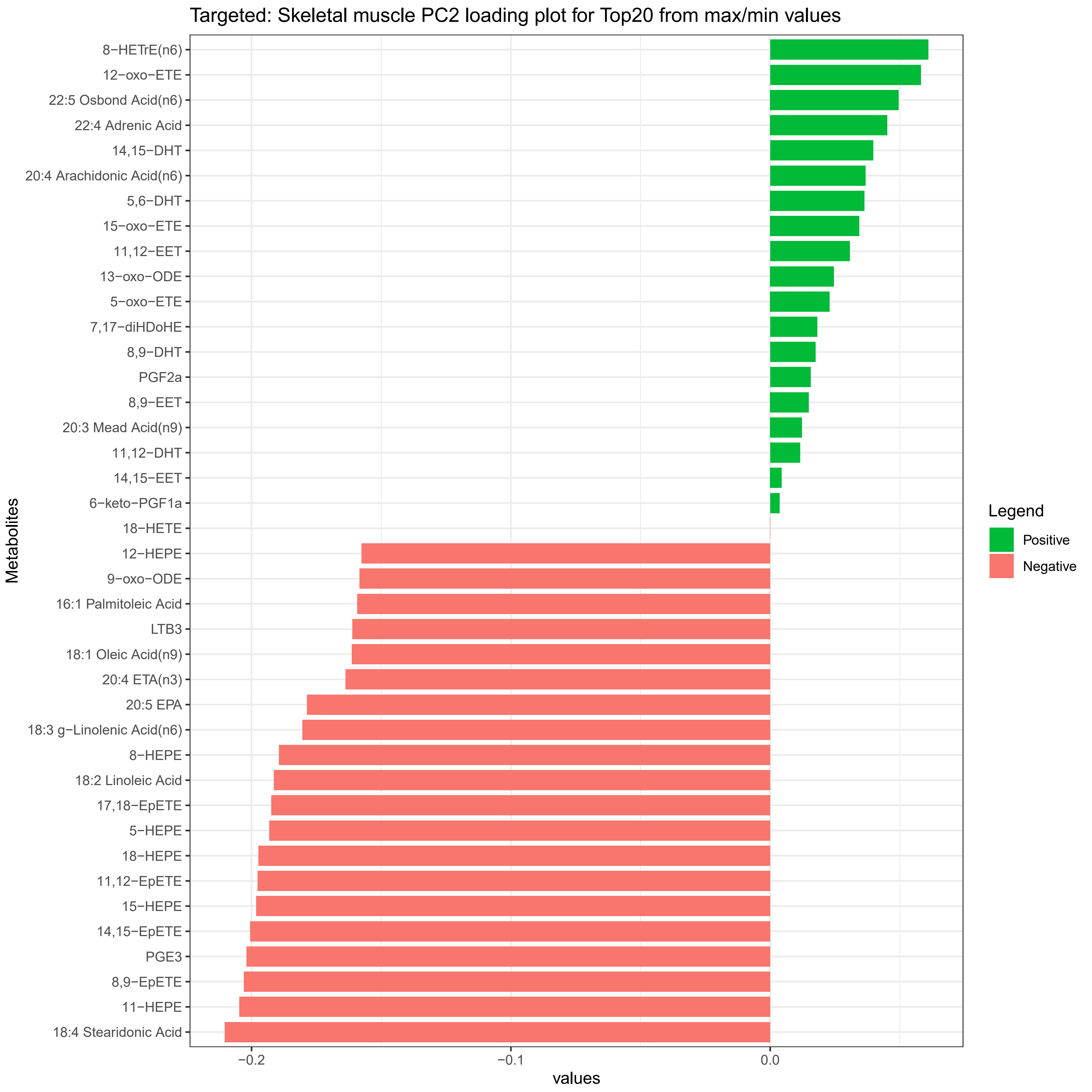

Targeted: Skeletal muscle PC2 loading plot for Top20 from max/min values
8−HETrE(n6)
12−oxo−ETE
22:5 Osbond Acid(n6)
22:4 Adrenic Acid
14,15−DHT
20:4 Arachidonic Acid(n6)
5,6−DHT
15−oxo−ETE
11,12−EET
13−oxo−ODE
5−oxo−ETE
7,17−diHDoHE
8,9−DHT
PGF2a
8,9−EET
20:3 Mead Acid(n9)
11,12−DHT
14,15−EET
6−keto−PGF1a
18−HETE
12−HEPE
9−oxo−ODE
16:1 Palmitoleic Acid
LTB3
18:1 Oleic Acid(n9)
20:4 ETA(n3)
20:5 EPA
18:3 g−Linolenic Acid(n6)
8−HEPE
18:2 Linoleic Acid
17,18−EpETE
5−HEPE
18−HEPE
11,12−EpETE
15−HEPE
14,15−EpETE
PGE3
8,9−EpETE
11−HEPE
18:4 Stearidonic Acid
Metabolites
−0.2
−0.1
0.0
values
Legend
Positive
Negative

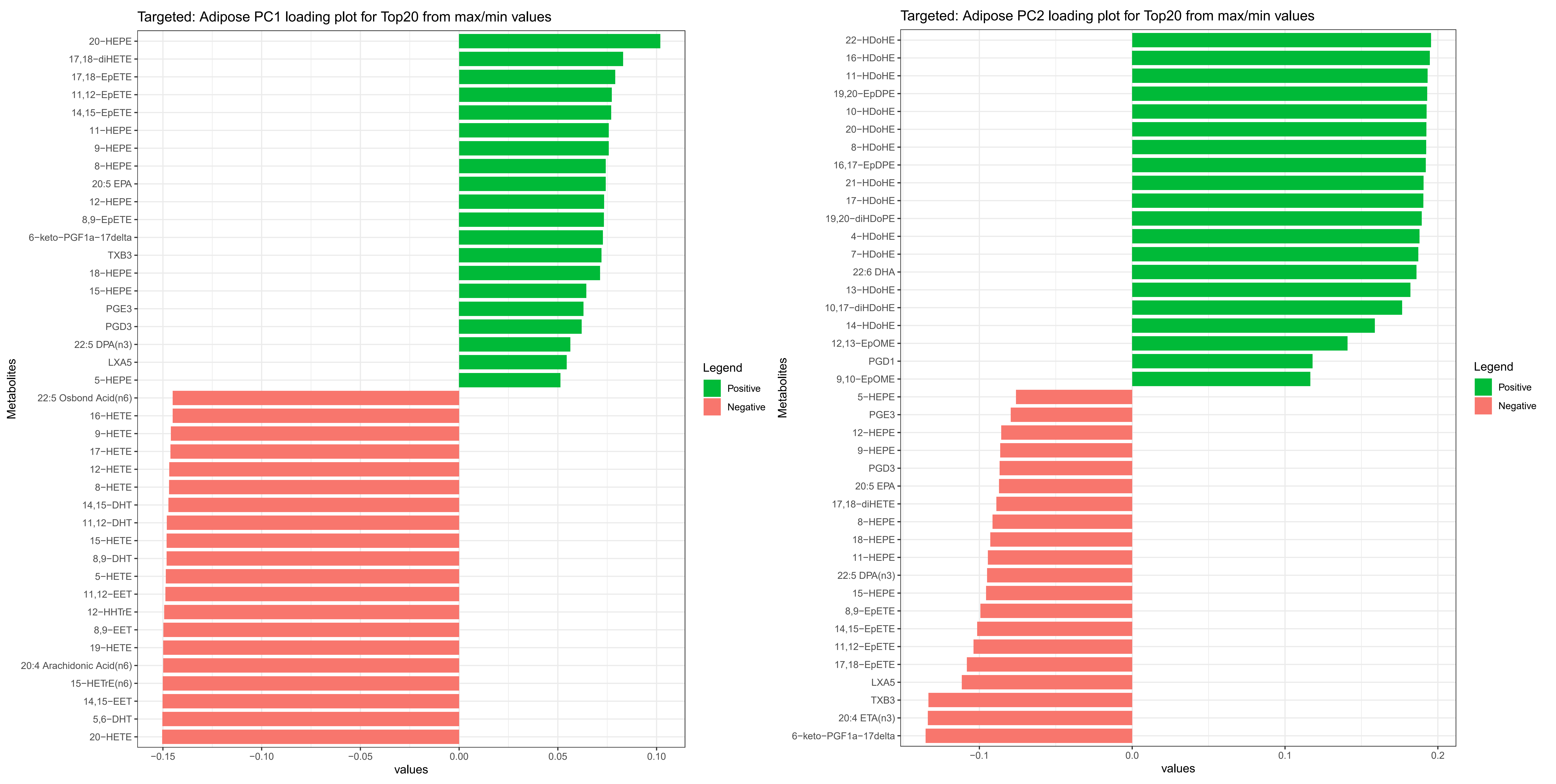

Targeted: Adipose PC1 loading plot for Top20 from max/min values
20−HEPE
17,18−diHETE
17,18−EpETE
11,12−EpETE
14,15−EpETE
11−HEPE
9−HEPE
8−HEPE
20:5 EPA
12−HEPE
8,9−EpETE
6−keto−PGF1a−17delta
TXB3
18−HEPE
15−HEPE
PGE3
PGD3
22:5 DPA(n3)
LXA5
5−HEPE
22:5 Osbond Acid(n6)
16−HETE
9−HETE
17−HETE
12−HETE
8−HETE
14,15−DHT
11,12−DHT
15−HETE
8,9−DHT
5−HETE
11,12−EET
12−HHTrE
8,9−EET
19−HETE
20:4 Arachidonic Acid(n6)
15−HETrE(n6)
14,15−EET
5,6−DHT
20−HETE
Metabolites
−0.15
−0.10
−0.05
0.00
0.05
0.10
values
Legend
Positive
Negative
Targeted: Adipose PC2 loading plot for Top20 from max/min values
22−HDoHE
16−HDoHE
11−HDoHE
19,20−EpDPE
10−HDoHE
20−HDoHE
8−HDoHE
16,17−EpDPE
21−HDoHE
17−HDoHE
19,20−diHDoPE
4−HDoHE
7−HDoHE
22:6 DHA
13−HDoHE
10,17−diHDoHE
14−HDoHE
12,13−EpOME
PGD1
9,10−EpOME
5−HEPE
PGE3
12−HEPE
9−HEPE
PGD3
20:5 EPA
17,18−diHETE
8−HEPE
18−HEPE
11−HEPE
22:5 DPA(n3)
15−HEPE
8,9−EpETE
14,15−EpETE
11,12−EpETE
17,18−EpETE
LXA5
TXB3
20:4 ETA(n3)
6−keto−PGF1a−17delta
Metabolites
−0.1
0.0
0.1
0.2
values
Legend
Positive
Negative

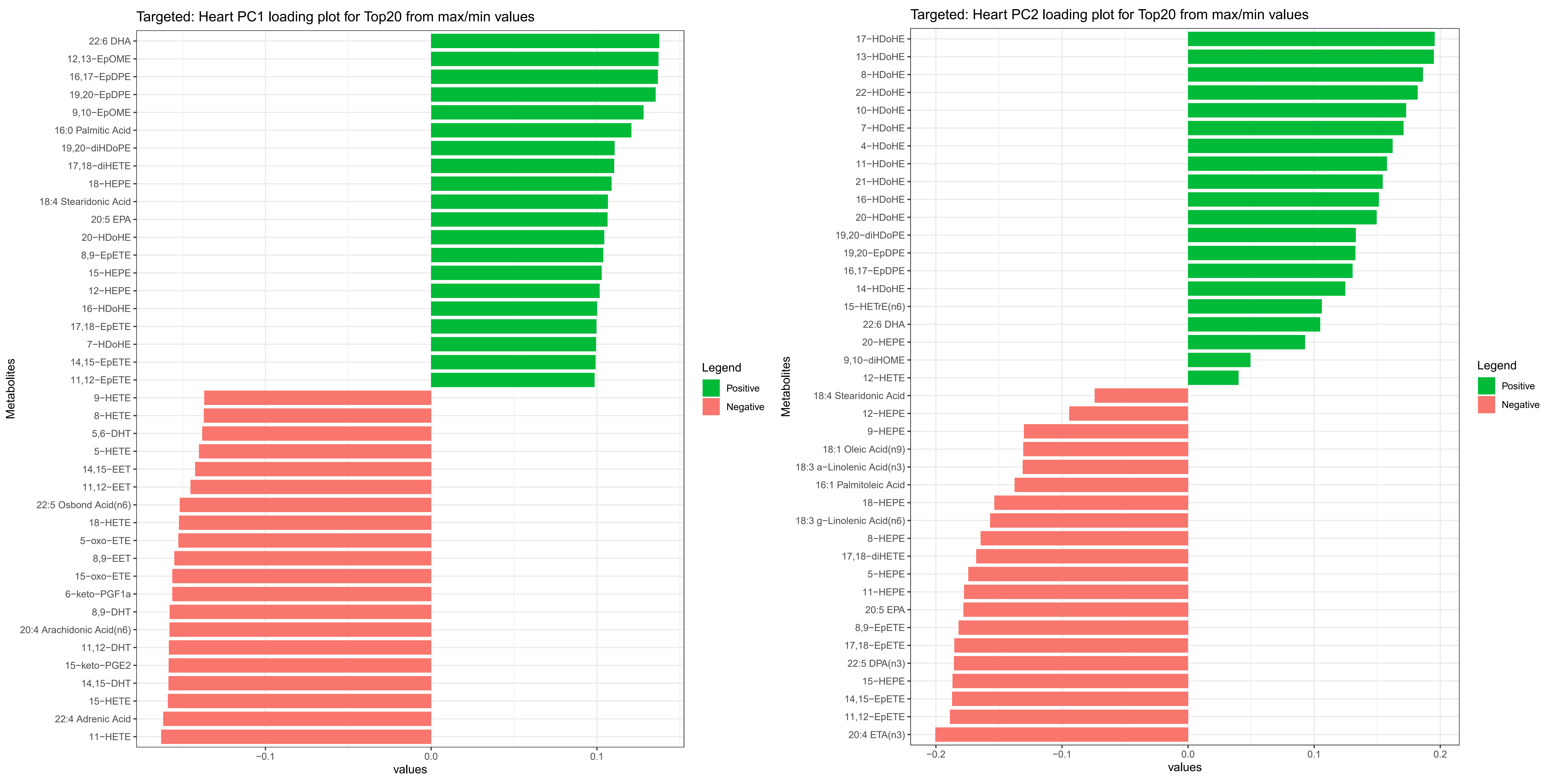

Targeted: Heart PC1 loading plot for Top20 from max/min values
22:6 DHA
12,13−EpOME
16,17−EpDPE
19,20−EpDPE
9,10−EpOME
16:0 Palmitic Acid
19,20−diHDoPE
17,18−diHETE
18−HEPE
18:4 Stearidonic Acid
20:5 EPA
20−HDoHE
8,9−EpETE
15−HEPE
12−HEPE
16−HDoHE
17,18−EpETE
7−HDoHE
14,15−EpETE
11,12−EpETE
9−HETE
8−HETE
5,6−DHT
5−HETE
14,15−EET
11,12−EET
22:5 Osbond Acid(n6)
18−HETE
5−oxo−ETE
8,9−EET
15−oxo−ETE
6−keto−PGF1a
8,9−DHT
20:4 Arachidonic Acid(n6)
11,12−DHT
15−keto−PGE2
14,15−DHT
15−HETE
22:4 Adrenic Acid
11−HETE
Metabolites
−0.1
0.0
0.1
values
Legend
Positive
Negative
Targeted: Heart PC2 loading plot for Top20 from max/min values
17−HDoHE
13−HDoHE
8−HDoHE
22−HDoHE
10−HDoHE
7−HDoHE
4−HDoHE
11−HDoHE
21−HDoHE
16−HDoHE
20−HDoHE
19,20−diHDoPE
19,20−EpDPE
16,17−EpDPE
14−HDoHE
15−HETrE(n6)
22:6 DHA
20−HEPE
9,10−diHOME
12−HETE
18:4 Stearidonic Acid
12−HEPE
9−HEPE
18:1 Oleic Acid(n9)
18:3 a−Linolenic Acid(n3)
16:1 Palmitoleic Acid
18−HEPE
18:3 g−Linolenic Acid(n6)
8−HEPE
17,18−diHETE
5−HEPE
11−HEPE
20:5 EPA
8,9−EpETE
17,18−EpETE
22:5 DPA(n3)
15−HEPE
14,15−EpETE
11,12−EpETE
20:4 ETA(n3)
Metabolites
−0.2
−0.1
0.0
0.1
0.2
values
Legend
Positive
Negative

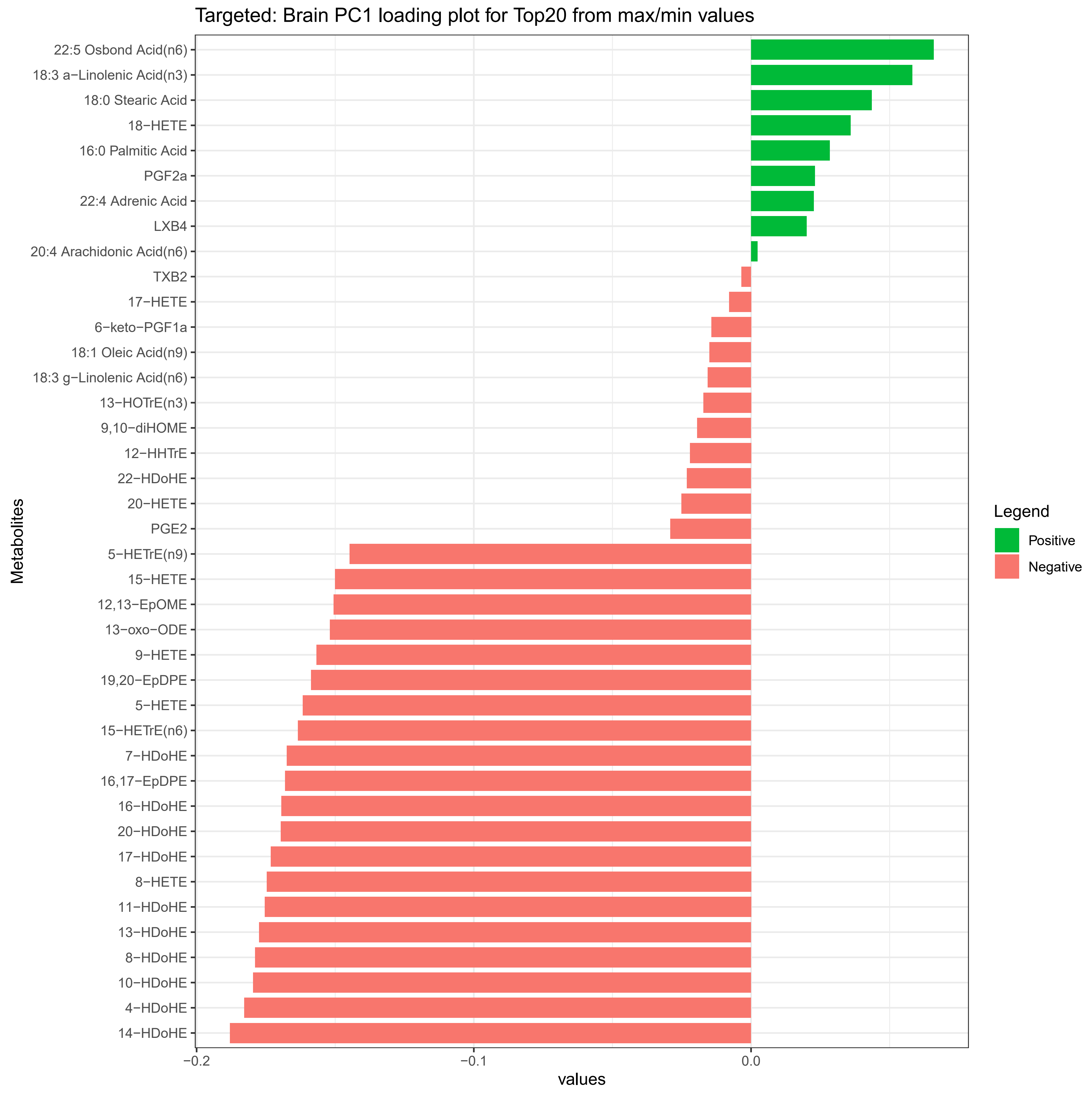
Targeted: Brain PC1 loading plot for Top20 from max/min values
22:5 Osbond Acid(n6)
18:3 a−Linolenic Acid(n3)
18:0 Stearic Acid
18−HETE
16:0 Palmitic Acid
PGF2a
22:4 Adrenic Acid
LXB4
20:4 Arachidonic Acid(n6)
TXB2
17−HETE
6−keto−PGF1a
18:1 Oleic Acid(n9)
18:3 g−Linolenic Acid(n6)
13−HOTrE(n3)
9,10−diHOME
12−HHTrE
22−HDoHE
20−HETE
PGE2
5−HETrE(n9)
15−HETE
12,13−EpOME
13−oxo−ODE
9−HETE
19,20−EpDPE
5−HETE
15−HETrE(n6)
7−HDoHE
16,17−EpDPE
16−HDoHE
20−HDoHE
17−HDoHE
8−HETE
11−HDoHE
13−HDoHE
8−HDoHE
10−HDoHE
4−HDoHE
14−HDoHE
Metabolites
−0.2
−0.1
0.0
values
Legend
Positive
Negative

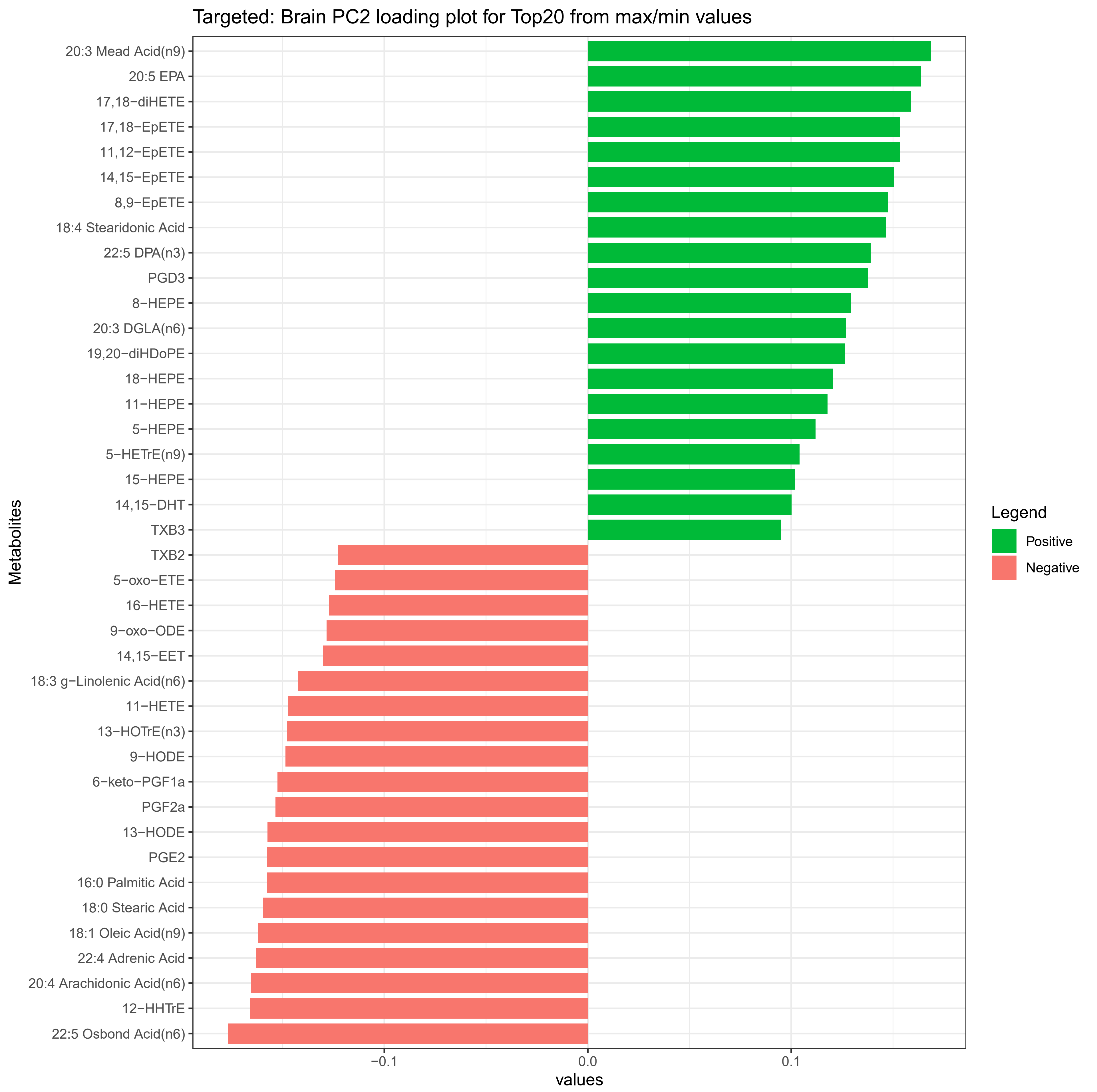
Targeted: Brain PC2 loading plot for Top20 from max/min values
20:3 Mead Acid(n9)
20:5 EPA
17,18−diHETE
17,18−EpETE
11,12−EpETE
14,15−EpETE
8,9−EpETE
18:4 Stearidonic Acid
22:5 DPA(n3)
PGD3
8−HEPE
20:3 DGLA(n6)
19,20−diHDoPE
18−HEPE
11−HEPE
5−HEPE
5−HETrE(n9)
15−HEPE
14,15−DHT
TXB3
TXB2
5−oxo−ETE
16−HETE
9−oxo−ODE
14,15−EET
18:3 g−Linolenic Acid(n6)
11−HETE
13−HOTrE(n3)
9−HODE
6−keto−PGF1a
PGF2a
13−HODE
PGE2
16:0 Palmitic Acid
18:0 Stearic Acid
18:1 Oleic Acid(n9)
22:4 Adrenic Acid
20:4 Arachidonic Acid(n6)
12−HHTrE
22:5 Osbond Acid(n6)
Metabolites
−0.1
0.0
0.1
values
Legend
Positive
Negative
