# Supplementary figures and images for: Characterization of Lipid Profiles after Dietary Intake of Polyunsaturated Fatty Acids Using Integrated Untargeted and Targeted Lipidomics
Source: Metabolites. 2019 Oct 21;9(10):241. doi: 10.3390/metabo9100241 (PMC6836067; doi:10.3390/metabo9100241)

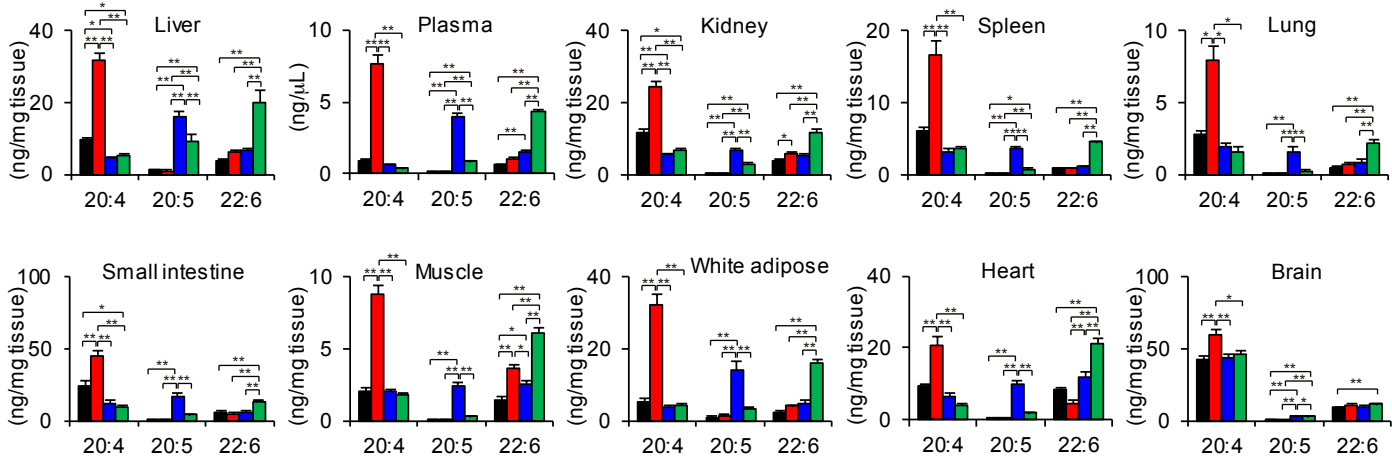

Supplement: Supplementary file 1 [file metabolites-09-00241-s001.zip › Supplementary Files/Supplementary Figure 3 new vs2.pdf]

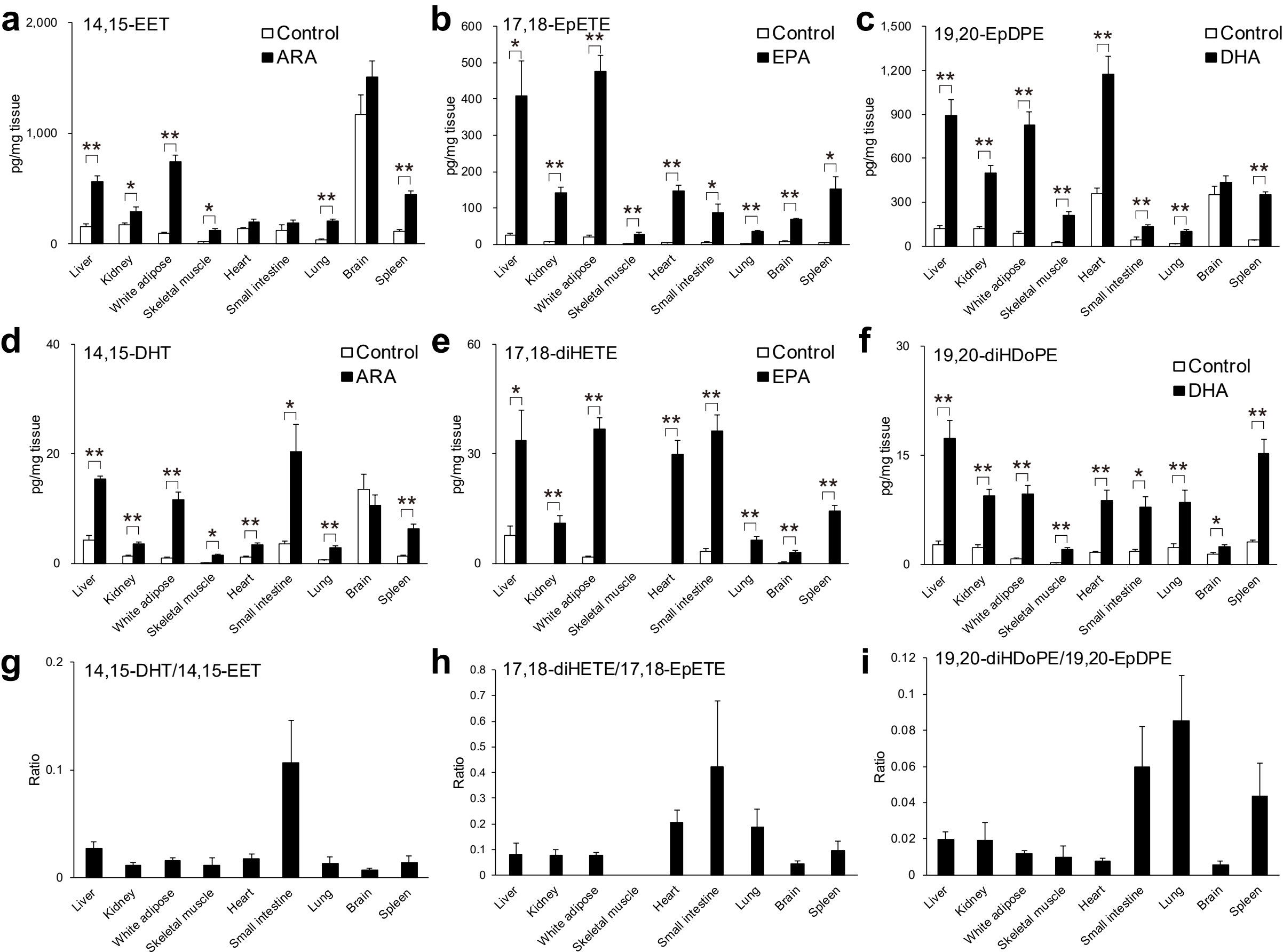

Supplement: Supplementary file 1 [file metabolites-09-00241-s001.zip › Supplementary Files/Supplementary Figure 4 new.pdf]

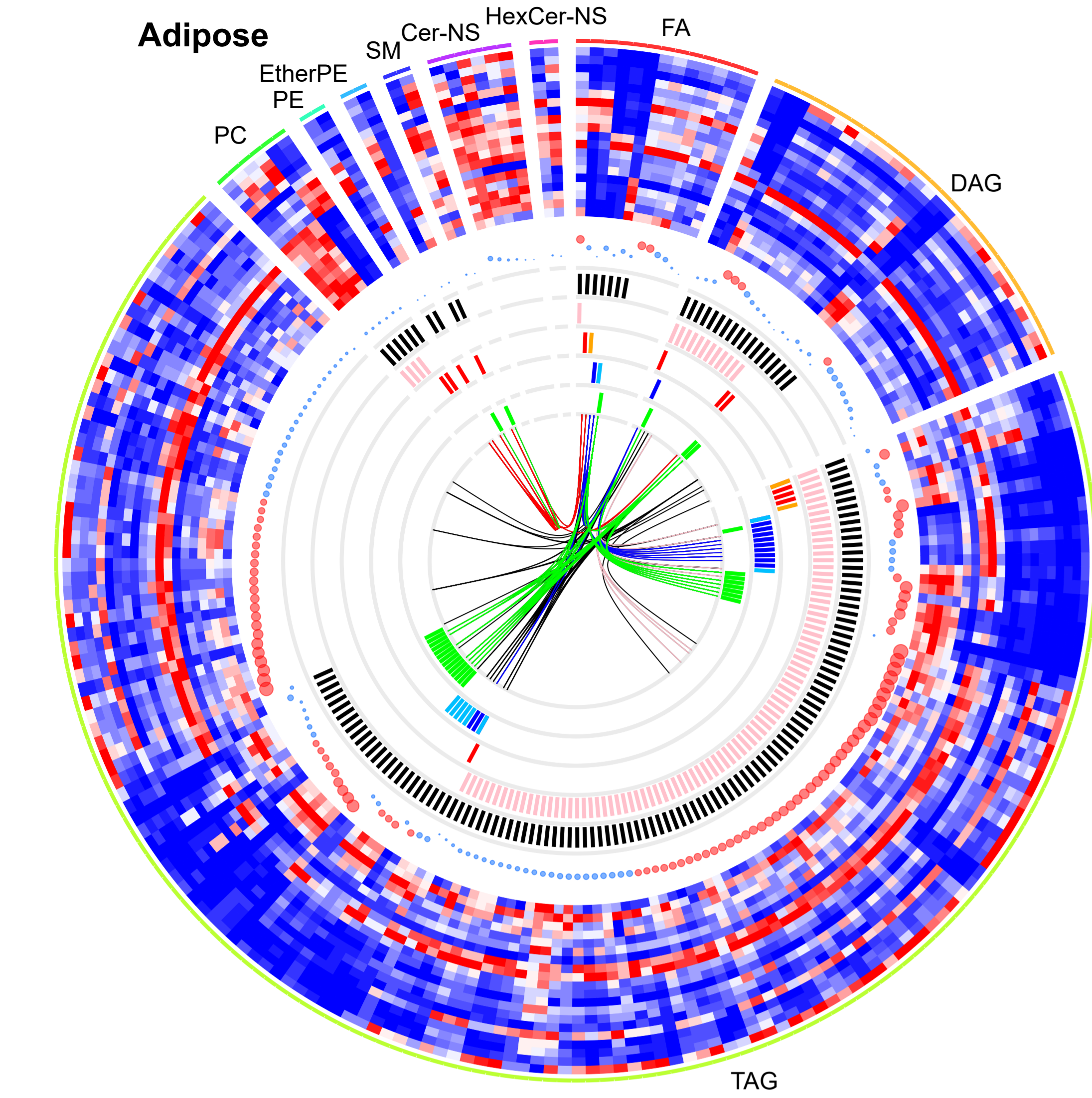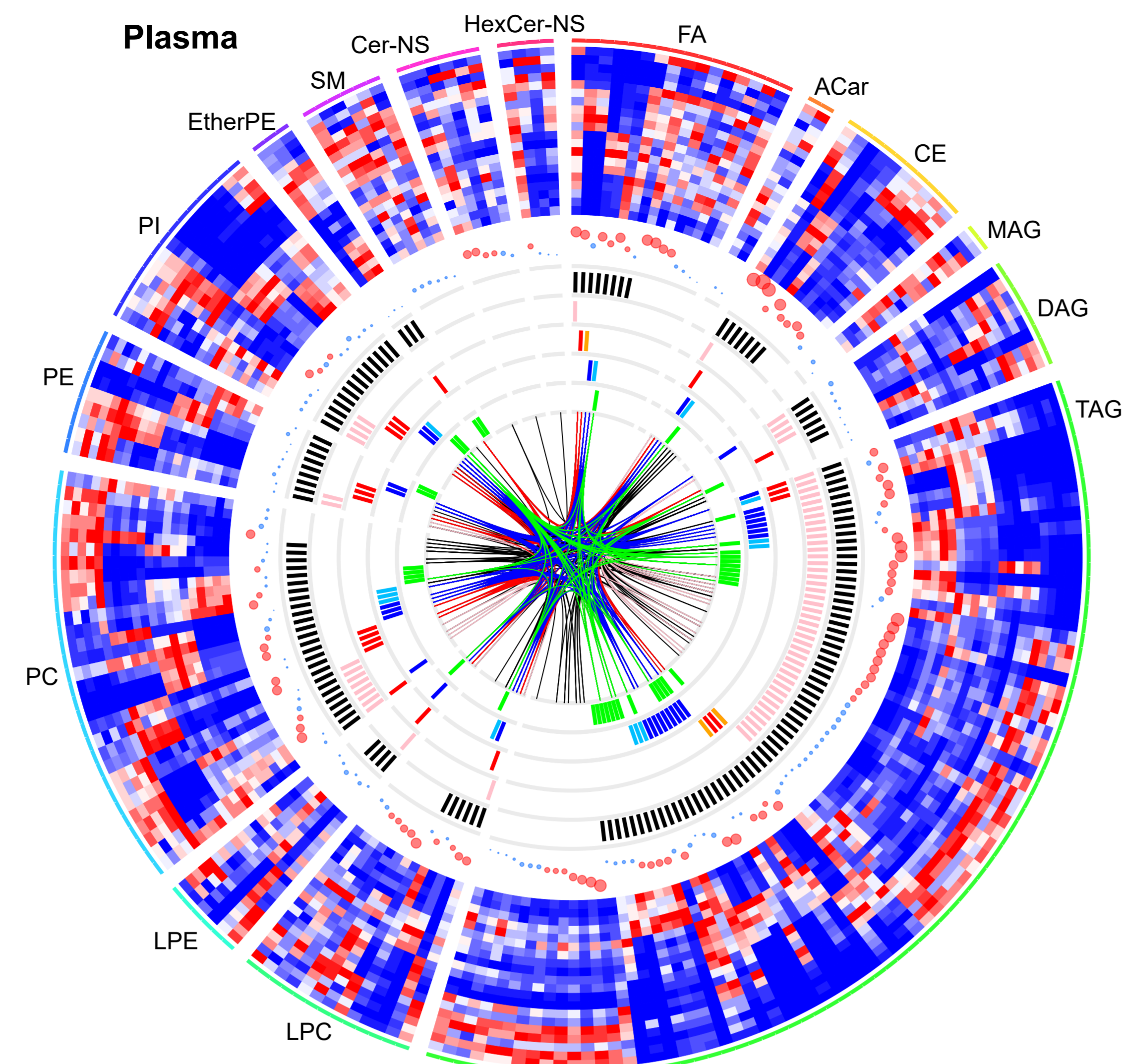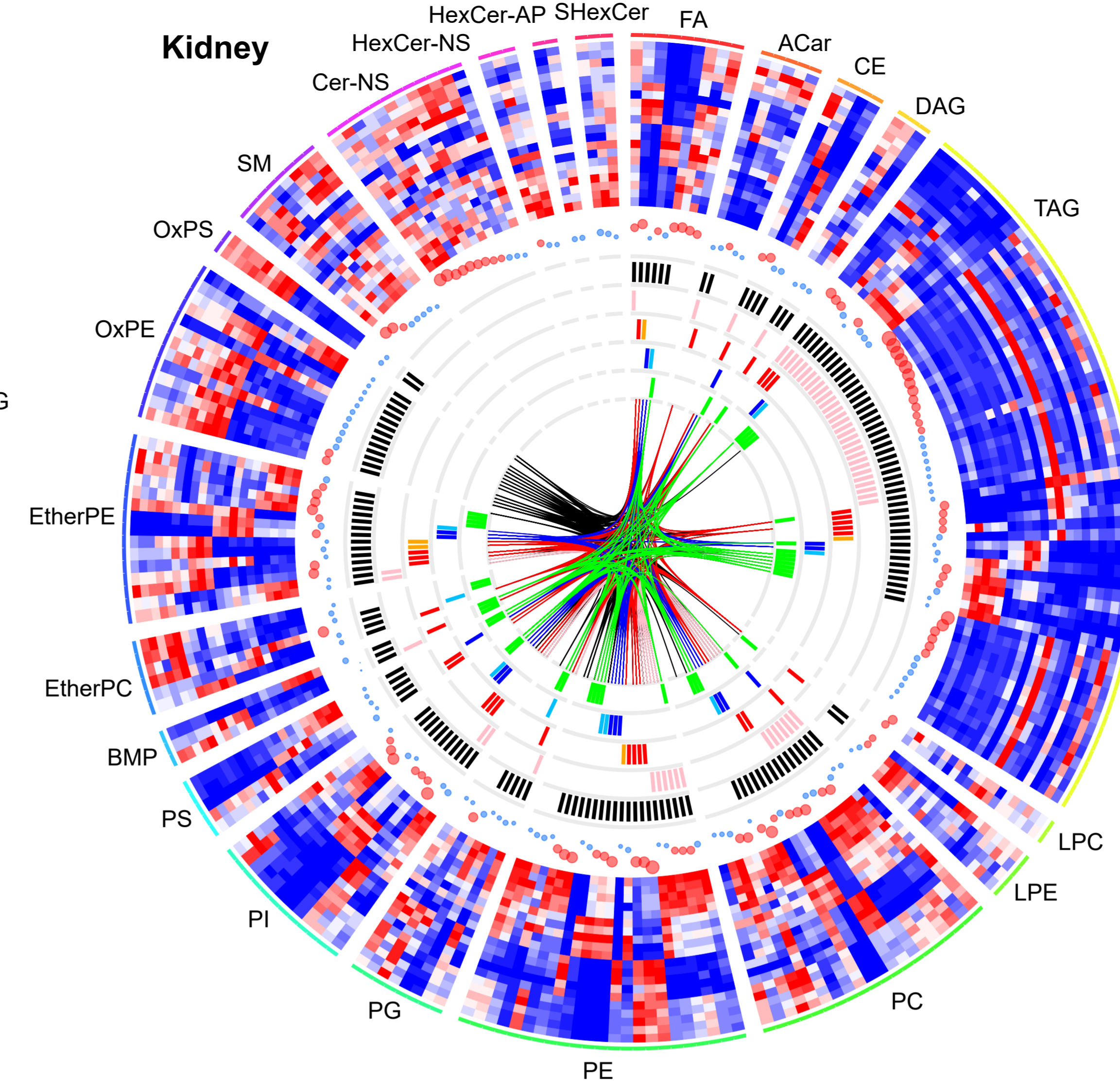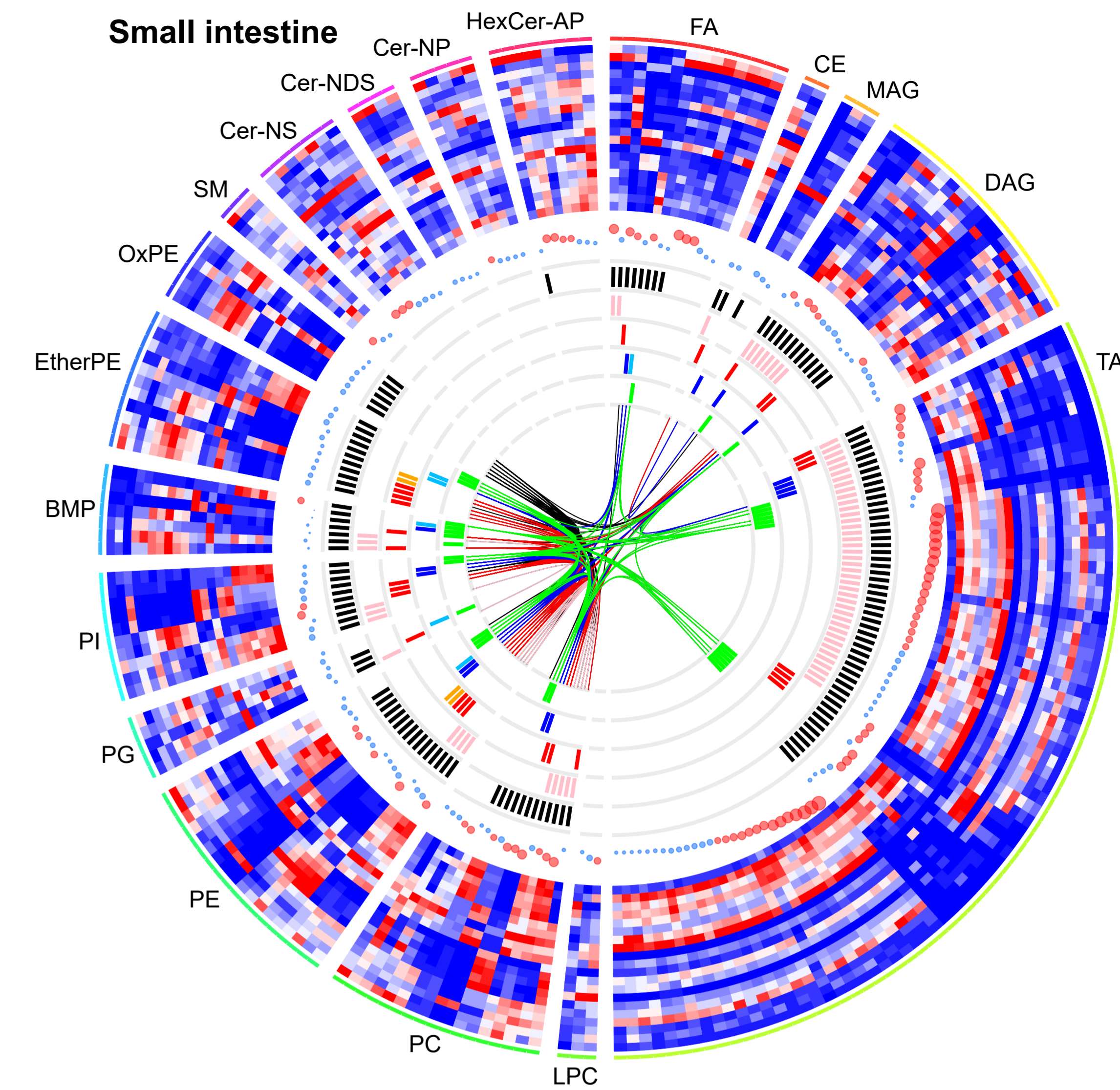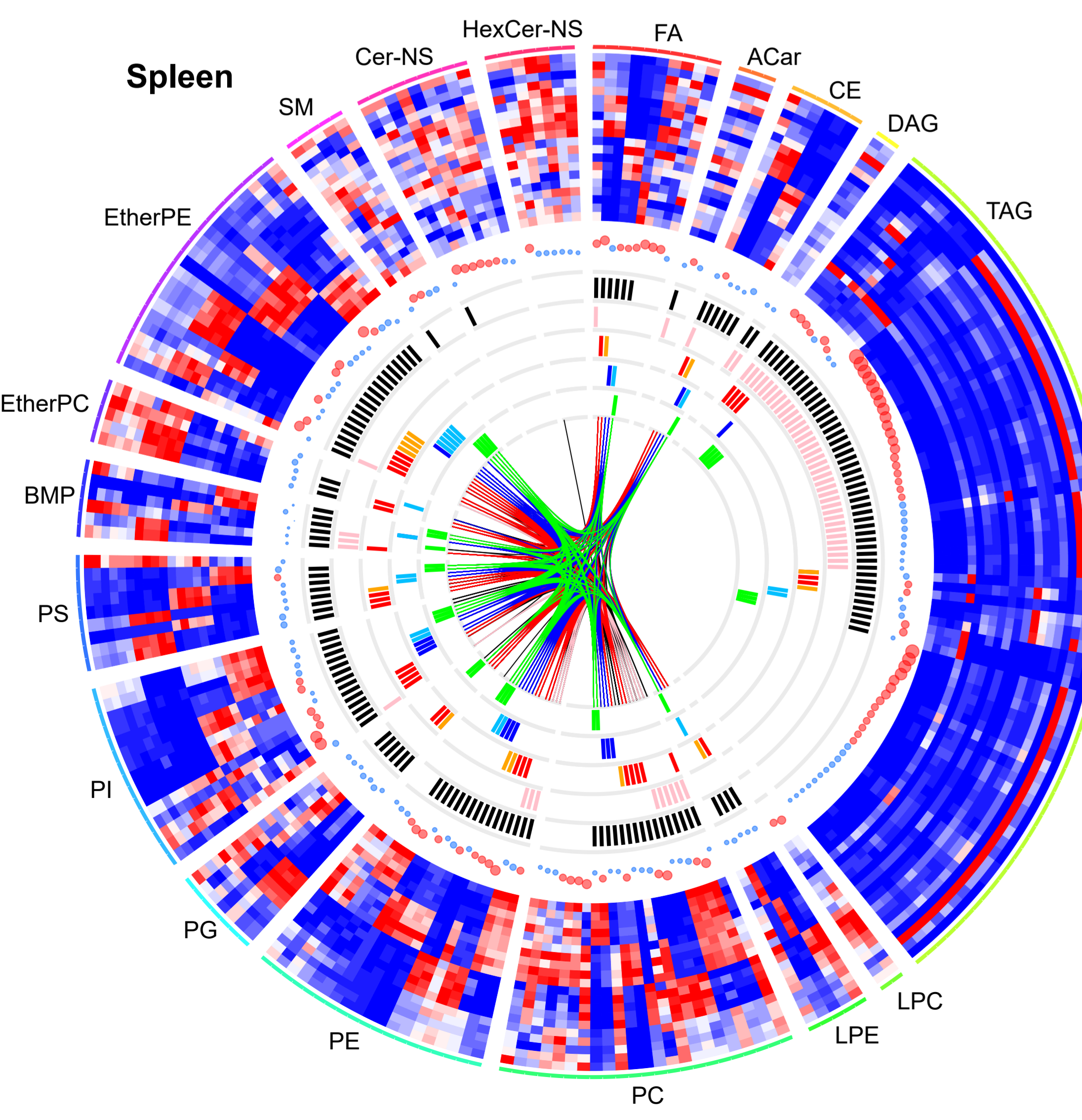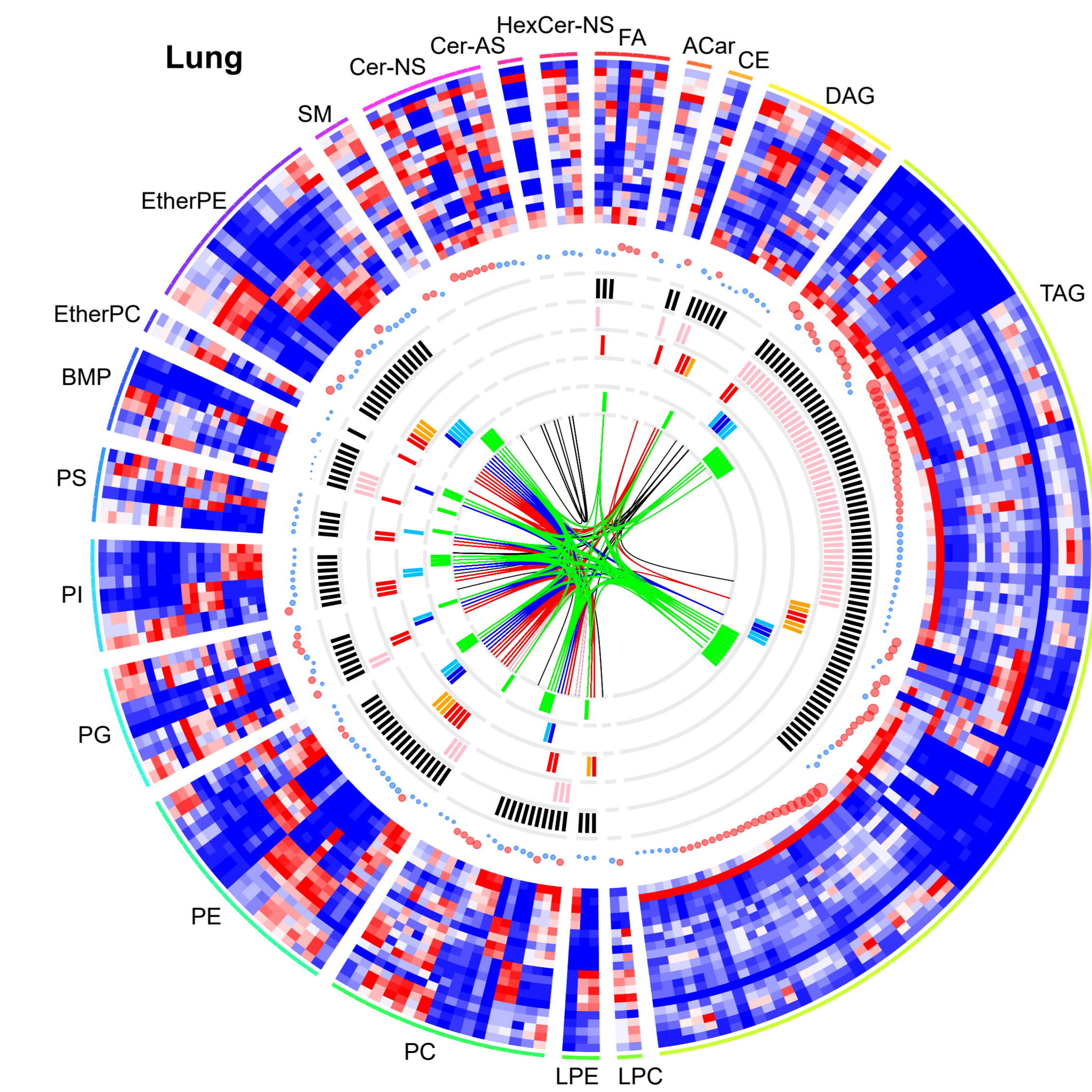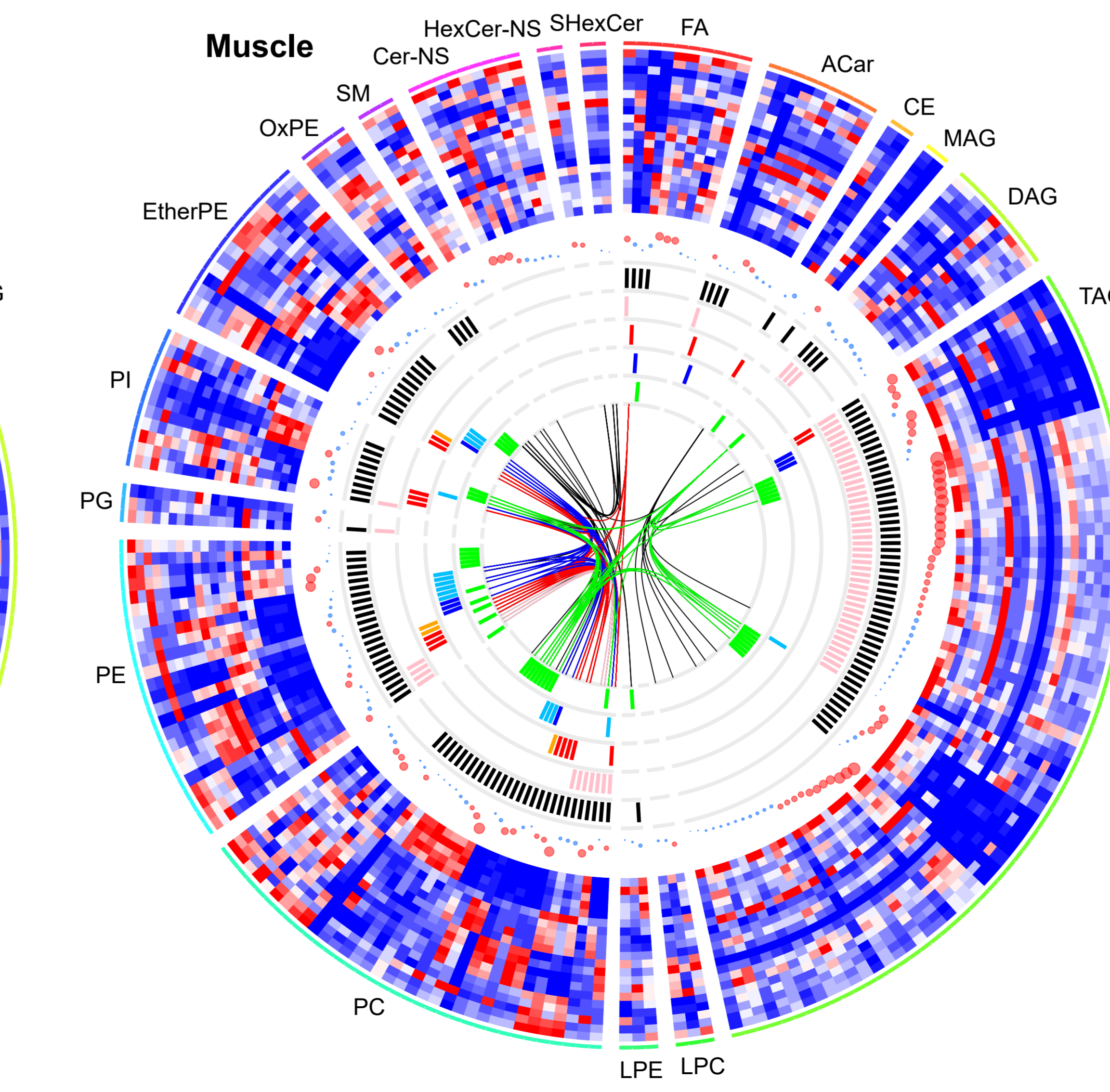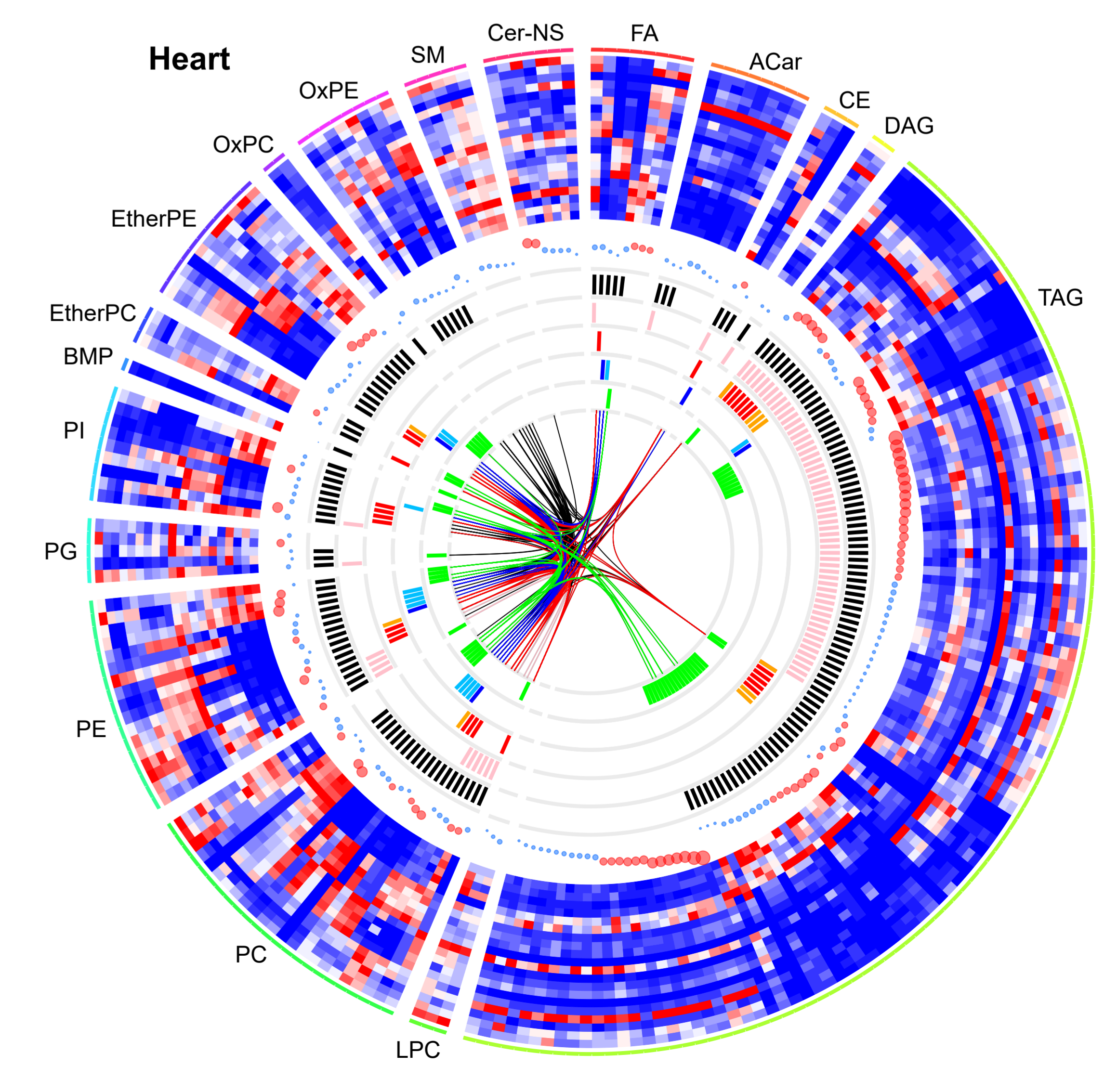

Supplement: Supplementary file 1 [file metabolites-09-00241-s001.zip › Supplementary Files/Supplementary Figure 5.pdf]
